# Half-Sandwich Zirconium and Hafnium Amidoborane Complexes: Precursors of Hydride Derivatives

**DOI:** 10.1021/acs.inorgchem.3c02826

**Published:** 2024-04-03

**Authors:** Maider Greño, Adrián Pérez-Redondo, José Torrijos, Víctor Varela-Izquierdo, Carlos Yélamos

**Affiliations:** Departamento de Química Orgánica y Química Inorgánica, Instituto de Investigación Química “Andrés M. del Río” (IQAR), Universidad de Alcalá, 28805 Alcalá de Henares, Madrid, Spain

## Abstract

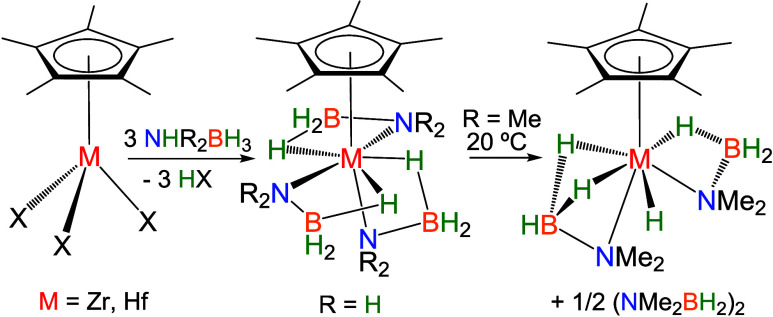

Half-sandwich zirconium(IV) and hafnium(IV) complexes with amidoborane and hydride ligands have been isolated in the stoichiometric reactions of mono(pentamethylcyclopentadienyl)metal alkyl and amido derivatives with the amine–boranes NHR_2_BH_3_ (R_2_ = H_2_, Me_2_, H*t*Bu). Treatment of the tris(trimethylsilylmethyl) complexes [M(η^5^-C_5_Me_5_)(CH_2_SiMe_3_)_3_] with NH_3_BH_3_ (3 equiv) gives the seven-coordinate species [M(η^5^-C_5_Me_5_)(NH_2_BH_3_)_3_] (M = Zr (**1**), Hf (**2**)) with three κ^2^*N,H*-NH_2_BH_3_ ligands. The tris(neophyl) [M(η^5^-C_5_Me_5_)(CH_2_CMe_2_Ph)_3_] or tris(dimethylamido) [M(η^5^-C_5_Me_5_)(NMe_2_)_3_] derivatives react with NHMe_2_BH_3_ (≥3 equiv) to afford bis(dimethylamidoborane) hydride complexes [M(η^5^-C_5_Me_5_)H(NMe_2_BH_3_)_2_] (M = Zr (**3**), Hf (**4**)) via thermally unstable [M(η^5^-C_5_Me_5_)(NMe_2_BH_3_)_3_] species. The reaction of [M(η^5^-C_5_Me_5_)(NMe_2_)_3_] and NH_2_*t*BuBH_3_ (≥4 equiv) affords analogous mixed amidoborane hydride derivatives [M(η^5^-C_5_Me_5_)H(NH*t*BuBH_3_)(NMe_2_BH_3_)] (M = Zr (**5**), Hf (**6**)) with κ^2^*N,H*-NH*t*BuBH_3_ and κ^3^*N,H,H*-NMe_2_BH_3_ ligands. The addition of NHR_2_BH_3_ (≥1 equiv) on the mono(dimethylamido) complexes [M(η^5^-C_5_Me_5_)Cl_2_(NMe_2_)] in hexane leads to the precipitation of the ionic compounds [(NHR_2_)_2_BH_2_][{M(η^5^-C_5_Me_5_)Cl_2_}_2_(μ-H)_3_] (R_2_ = Me_2_, M = Zr (**7**), Hf (**8**); R_2_ = H*t*Bu, M = Zr (**9**), Hf (**10**)). Molecular hydride species [Cl_2_(η^5^-C_5_Me_5_)M(μ-Cl)(μ-H)_2_M(η^5^-C_5_Me_5_)Cl(NH_2_*t*Bu)] (M = Zr (**11**), Hf (**12**)) could be isolated from mixtures of complexes [M(η^5^-C_5_Me_5_)Cl_2_(NMe_2_)] and lower ratios of NH_2_*t*BuBH_3_. The zirconium complex **11** decomposes in solution to give the mononuclear *tert*-butylamido derivative [Zr(η^5^-C_5_Me_5_)Cl_2_(NH*t*Bu)] (**13**) along with other byproducts.

## Abstract 2

Zirconium and hafnium trialkyl derivatives [M(η^5^-C_5_Me_5_)(CH_2_SiMe_3_)_3_] react with ammonia−borane to yield thermally stable tris(amidoborane) complexes [M(η^5^-C_5_Me_5_)(NH_2_BH_3_)_3_] (M = Zr, Hf), whereas analogous treatment of [M(η^5^-C_5_Me_5_)X_3_] (X = CH_2_CMe_2_Ph, NMe_2_) with NHMe_2_BH_3_ at room temperature produces bis(dimethylamidoborane) hydride complexes [M(ηη^5^-C_5_Me_5_)H(NMe_2_BH_3_)_2_] via thermally unstable [M(η^5^-C_5_Me_5_)(NMe_2_BH_3_)_3_] intermediates.

## Introduction

The dehydrogenation/dehydrocoupling of amine–borane adducts NHR^1^R^2^BH_3_ (R^1^, R^2^ = H, alkyl) mediated by metal complexes has been intensively studied over the last two decades.^1^ Early efforts in this research area were motivated by the potential application of these systems for chemical hydrogen storage, but nowadays, there is an increasing research interest in the preparation of polymeric materials with B–N backbones. Among the numerous metal complexes from across the periodic table capable of promoting these reactions under mild conditions, group 4 derivatives have received considerable attention as highly active homogeneous amine–borane dehydrocoupling catalysts. In particular, catalytic systems based on titanium are among the most studied in this field,^2−12^ and they are usually more active than the zirconium and hafnium analogues.^3,5,7,13−19^ The vast majority of this work was performed with metallocene-type complexes [M(η^5^-C_5_R_5_)_2_X_n_], but a few nonmetallocene group 4 systems are also known.^5,18,19^

Mechanistic proposals for dehydrogenation of amine–boranes catalyzed by *s*-block and early transition-metal systems suggest that the metal centers activate the substrates at the N–H bond with the formation of amidoborane complexes as intermediates.^1,20,21^ An extensive chemistry has been developed with *s*-block metal amidoborane compounds by Harder and Hill groups, including their role as catalysts and intermediates in these dehydrocoupling reactions.^22,23^ In contrast, transition-metal complexes containing amidoborane (NR_2_BH_3_)^−^ ligands remain rare in the literature. The first group that structurally characterized transition-metal amidoborane complexes was that of Roesler in 2009. They synthesized zirconium(IV) complexes [Zr(η^5^-C_5_R_5_)_2_(NH_2_BH_3_)X] (R = H, Me; X = H, Cl) by the reaction of [Zr(η^5^-C_5_R_5_)_2_Cl_2_] with [Li(NH_2_BH_3_)].^24^ Later, McGrady and co-workers reported that the analogous treatment of [Ti(η^5^-C_5_H_5_)_2_Cl_2_] with [Li(NH_2_BH_3_)] led to the paramagnetic titanium(III) compound [Ti(η^5^-C_5_H_5_)_2_(NH_2_BH_3_)].^25^ Lancaster and co-workers prepared a series of metallocene-type sandwich zirconium(IV) and hafnium(IV) complexes with amidoborane ligands [NH_2_B(C_6_F_5_)*_n_*H_3–*n*_]^−^ (*n* = 1 or 2) by analogous salt metathesis reactions.^26^ Within a long-standing research line in the dehydrocoupling of amine–boranes catalyzed by group 4 metallocenes, Manners group reported that the reactions of [M(η^5^-C_5_H_5_)_2_Cl_2_] with [Li(NMe_2_BH_3_)] led to the isolation of [Ti(η^5^-C_5_H_5_)_2_(NMe_2_BH_3_)] and [Zr(η^5^-C_5_H_5_)_2_H(NMe_2_BH_3_)].^7^ While the latter zirconium(IV) complex showed very poor activity, the former paramagnetic titanium(III) species is active in dimethylamine–borane dehydrocoupling. Crystals of the analogous titanium(III) methylamidoborane derivative [Ti(η^5^-C_5_Me_5_)_2_(NHMeBH_3_)] were isolated in the dehydropolymerization of NH_2_MeBH_3_ catalyzed by [Ti(η^5^-C_5_Me_5_)_2_Me], but attempts to synthesize this complex via salt metathesis were not successful.^11^ Those group 4 metallocene complexes show the amidoborane ligands bonded to the metal center through the nitrogen and a single hydrogen atom of the BHR_2_ group, namely, κ^2^*N*,*H*-NR_2_BHR_2_ coordination mode, as observed in other recently reported scandium^27^ and cobalt^28^ complexes. In contrast, the group of Waterman isolated crystals of the triamidoamine-supported zirconium(IV) complex [Zr(NN_3_)(NMe_2_BH_3_)] (NN_3_ = N(CH_2_CH_2_NSiMe_3_)_3_) with the dimethylamidoborane ligand coordinating in a tridentate fashion through the nitrogen atom and two hydrides of the BH_3_ group.^19^ This κ^3^*N*,*H*,*H* coordination mode is also documented in group 2 and rare-earth metal amidoborane complexes.^22,23,29^ The M···H–B interactions in amidoborane ligands coordinated to the metal centers resemble the β-agostic M···H–C interactions in metal alkyl complexes and β-hydride elimination results in the formation of aminoboranes (NR_2_BH_2_)_n_ and metal hydride species.^24,25^

As part of a research program devoted to the development of half-sandwich group 4 hydride complexes,^30^ we have been interested in exploring the reactivity of amine–borane adducts with derivatives [M(η^5^-C_5_Me_5_)X_3_]. In particular, our group has reported the isolation of titanium(III) hydride or amidoborane complexes in the reactions of trialkyl derivatives [Ti(η^5^-C_5_Me_5_)R_3_] (R = CH_2_SiMe_3_, Me) with NHR_2_BH_3_ (R_2_ = Me_2_, H_2_, H*t*Bu).^31,32^ The reaction of the zirconium and hafnium analogues [M(η^5^-C_5_Me_5_)(CH_2_SiMe_3_)_3_] with NHMe_2_BH_3_ gave thermally unstable dialkyl(dimethylamidoborane) compounds [M(η^5^-C_5_Me_5_)(CH_2_SiMe_3_)_2_(NMe_2_BH_3_)] without change in the oxidation state of the metal centers.^31^ In this article, we describe the compounds obtained by means of the treatment of several mono(pentamethylcyclopentadienyl) zirconium(IV) and hafnium(IV) alkyl and amido complexes with amine–borane adducts NHR_2_BH_3_ (R_2_ = H_2_, Me_2_, H*t*Bu). The amidoborane derivatives isolated and their decomposition pathways via β-hydride elimination to give hydride complexes are discussed. We have previously communicated our preliminary results on the reaction of [Zr(η^5^-C_5_Me_5_)(NMe_2_)_*n*_Cl_3-n_] and NHR_2_BH_3_ (R_2_ = Me_2_, H*t*Bu).^33^

## Experimental Section

### General Considerations

All manipulations were carried out under an argon atmosphere using Schlenk line or glovebox techniques. Toluene and hexane were distilled from Na/K alloy just before use. Tetrahydrofuran was distilled from purple solutions of sodium benzophenone just prior to use. NMR solvents were dried with Na/K alloy (C_6_D_6_, C_4_D_8_O) or calcium hydride (CDCl_3_) and distilled before use. Oven-dried glassware was repeatedly evacuated with a pumping system (ca. 1 × 10^–3^ Torr) and subsequently filled with inert gas. Ammonia borane (NH_3_BH_3_), *N,N*-dimethylamine–borane (NHMe_2_BH_3_), *N*-*tert*-butylamine–borane (NH_2_*t*BuBH_3_), and [Li(NMe_2_)] (95%) were acquired from Sigma-Aldrich. [Li(CH_2_CMe_2_Ph)] was prepared by the reaction of lithium with PhCMe_2_CH_2_Cl in a fashion similar to the analogous [Li(CH_2_SiMe_3_)].^34^ [M(η^5^-C_5_Me_5_)(CH_2_SiMe_3_)_3_] (M = Zr, Hf),^31^ [M(η^5^-C_5_Me_5_)Cl_3_] (M = Zr, Hf),^35^ [M(η^5^-C_5_Me_5_)(NMe_2_)_3_] (M = Zr,^36^ Hf^37^), and [Zr(η^5^-C_5_Me_5_)Cl_2_(NMe_2_)]^36^ were obtained following the reported procedures. We have already published the synthesis and characterization of derivatives [Zr(η^5^-C_5_Me_5_)H(NMe_2_BH_3_)_2_] (**3**), [(NHMe_2_)_2_BH_2_][{Zr(η^5^-C_5_Me_5_)Cl_2_}_2_(μ-H)_3_] (**7**), and [(NH_2_*t*Bu)_2_BH_2_][{Zr(η^5^-C_5_Me_5_)Cl_2_}_2_(μ-H)_3_] (**9**).^33^ No uncommon hazards are noted.

Samples for powder X-ray diffraction were prepared in a glovebox using capillaries of 0.7 mm, and the diffractograms were registered on an Empyrean Malvern Panalytical diffractometer. Samples for infrared spectroscopy were prepared as KBr pellets, and the spectra were obtained using an FT-IR-Frontier PerkinElmer spectrophotometer, or the IR spectra were recorded as a powder or a tetrahydrofuran solution using an attenuated total reflection (ATR) method on a Bruker FT-IR-α II spectrometer situated in an argon-filled glovebox. ^1^H, ^13^C{^1^H}, ^11^B, ^11^B{^1^H}, and ^1^H–^11^B HMBC NMR spectra were recorded on Varian Mercury-300, Varian Unity-500, or Bruker Avance Neo 400 spectrometers. ^1^H{^11^B} NMR spectra were obtained using a Bruker AV300 Avance spectrometer. Chemical shifts (δ, ppm) in the ^1^H and ^13^C{^1^H} NMR spectra are given relative to residual protons or to carbon of the solvent. Chemical shifts (δ, ppm) in the ^11^B and ^11^B{^1^H} NMR spectra are given relative to BF_3_·OEt_2_ as the external reference. Microanalyses (C, H, N) were performed in a PerkinElmer CHNS/O 2400 or a Leco CHNS-932 microanalyzer. The incomplete combustion of the samples could probably explain the values being consistently low in the elemental analysis of some amidoborane and hydride complexes.

### Synthesis of [Zr(η^5^-C_5_Me_5_)(NH_2_BH_3_)_3_] (**1**)

A mixture of 0.40 g (0.82 mmol) of [Zr(η^5^-C_5_Me_5_)(CH_2_SiMe_3_)_3_], 0.076 g (2.46 mmol) of NH_3_BH_3_, and 30 mL of toluene was stirred at room temperature for 20 h in a 100 mL Schlenk tube to give a colorless solution. After removing the volatile components of the solution under reduced pressure, a white solid was obtained, washed with hexane (2 × 10 mL), and finally vacuum-dried to afford **1** (0.19 g, 73%). IR (KBr, cm^–1^): ν̃ 3409 (s) (NH), 3336 (s) (NH), 2980 (m), 2952 (m), 2911 (s), 2862 (m), 2410 (vs) (BH_term_), 2382 (vs) (BH_term_), 2352 (vs) (BH_term_), 2328 (s) (BH_term_), 1920 (vs) (BH_bridging_), 1855 (s) (BH_bridging_), 1778 (s) (BH_bridging_), 1551 (m), 1517 (s), 1491 (m), 1425 (s), 1401 (s), 1380 (s), 1183 (vs), 1066 (m), 996 (vs), 885 (m), 854 (m), 830 (m), 807 (m), 615 (m). IR (thf, cm^–1^): ν̃ 3401 (w) (NH), 3325 (w) (NH), 2412 (s) (BH_term_), 2324 (m) (BH_term_), 1920 (m br.) (BH_bridging_), 1808 (w br.) (BH_bridging_). ^1^H NMR (500 MHz, C_6_D_6_, 20 °C): δ 1.64 (s, 15H; C_5_Me_5_), 1.44–0.10 (m br., 15H; N*H*_2_B*H*_3_). ^1^H NMR (300 MHz, C_4_D_8_O, 20 °C): δ 1.99 (s, 15H; C_5_Me_5_), 1.96–0.00 (m br., 15H; N*H*_2_B*H*_3_). ^1^H{^11^B} NMR (300 MHz, C_6_D_6_, 24 °C): δ 1.66 (s, 15H; C_5_Me_5_), 1.44–0.00 (m br., 15H; N*H*_2_B*H*_3_). ^13^C{^1^H} NMR (125 MHz, C_6_D_6_, 20 °C): δ 118.4 (*C*_5_Me_5_), 11.2 (C_5_*Me*_5_). ^11^B NMR (128 MHz, C_6_D_6_, 20 °C): δ −18 to −27 (m br.; BH_3_). ^11^B{^1^H} NMR (128 MHz, C_6_D_6_, 20 °C): δ −21.7 (s br.; BH_3_), −22.6 (s br.; BH_3_), −25.1 (s br.; BH_3_). Anal. calcd (%) for C_10_H_30_B_3_N_3_Zr (*M*_w_ = 316.03): C 38.01, H 9.57, N 13.30. Found: C 37.43, H 8.96, N 13.31.

### Synthesis of [Hf(η^5^-C_5_Me_5_)(NH_2_BH_3_)_3_] (**2**)

A mixture of 0.30 g (0.52 mmol) of [Hf(η^5^-C_5_Me_5_)(CH_2_SiMe_3_)_3_], 0.048 g (1.56 mmol) of NH_3_BH_3_, and 30 mL of tetrahydrofuran was stirred at 45 °C for 2 days in a 100 mL ampule (Teflon stopcock) to give a colorless solution. After removing the volatile components, the resulting oil was dissolved in 10 mL of toluene. After filtration and removing the volatile components of the filtrate under reduced pressure, a white solid was isolated, washed with hexane (2 × 10 mL), and finally vacuum-dried to give **2** (0.096 g, 46%). IR (KBr, cm^–1^): ν̃ 3397 (vs) (NH), 3328 (vs) (NH), 2954 (m), 2912 (s), 2862 (m), 2415 (vs) (BH_term_), 2361 (s) (BH_term_), 2336 (vs) (BH_term_), 1936 (vs) (BH_bridging_), 1811 (s) (BH_bridging_), 1521 (s), 1491 (m), 1431 (s), 1382 (s), 1198 (s), 1170 (s), 1006 (vs), 808 (m), 637 (m), 597 (w), 463 (w). IR (thf, cm^–1^): ν̃ 3410 (w) (NH), 3321 (w) (NH), 2414 (s) (BH_term_), 2333 (m) (BH_term_), 1936 (m br.) (BH_bridging_), 1811 (w br.) (BH_bridging_). NMR (500 MHz, C_6_D_6_, 20 °C): δ 1.71 (s, 15H; C_5_Me_5_), 1.50–0.10 (m br., 15H; N*H*_2_B*H*_3_). ^1^H{^11^B} NMR (300 MHz, C_6_D_6_, 24 °C): δ 1.71 (s, 15H; C_5_Me_5_), 1.50–0.00 (m br., 15H; N*H*_2_B*H*_3_). ^13^C{^1^H} NMR (125 MHz, C_6_D_6_, 20 °C): δ 116.3 (*C*_5_Me_5_), 11.1 (C_5_*Me*_5_). ^11^B NMR (96 MHz, C_6_D_6_, 20 °C): δ −18 to −28 (m br.; BH_3_). ^11^B{^1^H} NMR (96 MHz, C_6_D_6_, 20 °C): δ −21.6 (s br.; BH_3_), −22.7 (s br.; BH_3_), −24.6 (s br.; BH_3_). Anal. calcd (%) for C_10_H_30_B_3_HfN_3_ (*M*_w_ = 403.29): C 29.78, H 7.50, N 10.42. Found: C 30.10, H 7.52, N 10.54.

### Synthesis of [Zr(η^5^-C_5_Me_5_)(CH_2_CMe_2_Ph)_3_]

A mixture of 0.50 g (1.50 mmol) of [Zr(η^5^-C_5_Me_5_)Cl_3_], 0.64 g (4.57 mmol) of [Li(CH_2_CMe_2_Ph)], and 40 mL of hexane was stirred at room temperature for 20 h in a 100 mL Schlenk tube to give a gray solid and a colorless solution. After filtration, the volatile components of the solution were removed under reduced pressure to afford [Zr(η^5^-C_5_Me_5_)(CH_2_CMe_2_Ph)_3_] as a white solid (0.56 g, 60%). IR (KBr, cm^–1^): ν̃ 3084 (w), 3054 (w), 3021 (w), 2961 (vs), 2908 (vs), 2862 (s), 1599 (m), 1495 (s), 1443 (s), 1375 (m), 1269 (m), 1155 (w), 1082 (w), 1031 (m), 763 (vs), 699 (vs), 680 (m), 547 (m). ^1^H NMR (500 MHz, C_6_D_6_, 20 °C): δ 7.35 (m, 6H; *o*-C_6_H_5_), 7.24 (m, 6H; *m*-C_6_H_5_), 7.10 (m, 3H; *p*-C_6_H_5_), 1.64 (s, 15H; C_5_Me_5_), 1.43 (s, 18H; CH_2_C*Me*_2_Ph), 0.89 (s, 6H; C*H*_2_CMe_2_Ph). ^13^C{^1^H} NMR (125 MHz, C_6_D_6_, 20 °C): δ 152.9, 128.3, 126.0, 125.5 (C_6_H_5_), 119.1 (*C*_5_Me_5_), 94.6 (*C*H_2_CMe_2_Ph), 42.5 (CH_2_*C*Me_2_Ph), 34.7 (CH_2_C*Me*_2_Ph), 11.8 (C_5_*Me*_5_). Anal. calcd (%) for C_40_H_54_Zr (*M*_w_ = 626.09): C 76.74, H 8.69. Found: C 76.57, H 8.79.

### Synthesis of [Hf(η^5^-C_5_Me_5_)(CH_2_CMe_2_Ph)_3_]

Similarly to the preparation of [Zr(η^5^-C_5_Me_5_)(CH_2_CMe_2_Ph)_3_], the treatment of 0.50 g (1.19 mmol) of [Hf(η^5^-C_5_Me_5_)Cl_3_] with 0.51 g (3.64 mmol) of [Li(CH_2_CMe_2_Ph)] in 40 mL of hexane gave [Hf(η^5^-C_5_Me_5_)(CH_2_CMe_2_Ph)_3_] as a white solid (0.66 g, 78%). IR (KBr, cm^–1^): ν̃ 3086 (w), 3056 (w), 3021 (w), 2956 (vs), 2914 (vs), 2866 (s), 1600 (m), 1496 (s), 1444 (s), 1374 (m), 1361 (m), 1269 (m), 1166 (m), 1031 (m), 903 (w), 780 (s), 764 (vs), 700 (vs), 547 (m), 508 (w). ^1^H NMR (500 MHz, C_6_D_6_, 20 °C): δ 7.36 (m, 6H; *o*-C_6_H_5_), 7.25 (m, 6H; *m*-C_6_H_5_), 7.10 (m, 3H; *p*-C_6_H_5_), 1.68 (s, 15H; C_5_Me_5_), 1.43 (s, 18H; CH_2_C*Me*_2_Ph), 0.55 (s, 6H; C*H*_2_CMe_2_Ph). ^13^C{^1^H} NMR (125 MHz, C_6_D_6_, 20 °C): δ 153.4, 128.3, 126.1, 125.5 (C_6_H_5_), 118.9 (*C*_5_Me_5_), 104.0 (*C*H_2_CMe_2_Ph), 43.1 (CH_2_*C*Me_2_Ph), 35.3 (CH_2_C*Me*_2_Ph), 11.6 (C_5_*Me*_5_). Anal. calcd (%) for C_40_H_54_Hf (*M*_w_ = 713.36): C 67.35, H 7.63. Found: C 67.07, H 7.60.

### Synthesis of [Hf(η^5^-C_5_Me_5_)H(NMe_2_BH_3_)_2_] (**4**)

A mixture of 0.30 g (0.67 mmol) of [Hf(η^5^-C_5_Me_5_)(NMe_2_)_3_], 0.24 g (4.07 mmol) of NHMe_2_BH_3_, and 40 mL of hexane was stirred at room temperature for 16 h in a 100 mL Schlenk tube to give a colorless solution. After filtration, the volatile components of the solution were removed under reduced pressure to afford **4** as a white solid (0.15 g, 52%). IR (KBr, cm^–1^): ν̃ 3045 (m), 2989 (m), 2950 (s), 2899 (vs), 2832 (m), 2806 (m), 2795 (m), 2782 (m), 2475 (s) (BH_term_), 2432 (vs) (BH_term_), 2405 (s) (BH_term_), 2326 (m) (BH_term_), 2070 (s) (BH_bridging_), 2017 (w) (BH_bridging_), 1871 (s) (BH_bridging_), 1611 (vs) (HfH), 1459 (s), 1380 (s), 1306 (s), 1252 (s), 1177 (s), 1041 (m), 997 (vs), 934 (m), 721 (m), 640 (m). ^1^H NMR (300 MHz, C_6_D_6_, 20 °C): δ 11.39 (s br., 1H; Hf–H), 2.44 (s br., 6H; N*Me*MeBH_3_), 2.40 (s br., 6H; NMe*Me*BH_3_), 2.03 (s, 15H; C_5_Me_5_), 1.32 (q br., ^1^*J*(H,B) = 90.6 Hz, 6H; NMe_2_B*H*_3_). ^13^C{^1^H} NMR (75 MHz, C_6_D_6_, 20 °C): δ 118.7 (*C*_5_Me_5_), 51.8 (N*Me*MeBH_3_), 50.9 (NMe*Me*BH_3_), 12.5 (C_5_*Me*_5_). ^11^B NMR (128 MHz, C_6_D_6_, 20 °C): δ −7.3 (m br.; BH_3_). Anal. calcd (%) for C_14_H_34_B_2_HfN_2_ (*M*_w_ = 430.55): C 39.06, H 7.96, N 6.51. Found: C 38.45, H 7.73, N 7.14.

### Synthesis of [Zr(η^5^-C_5_Me_5_)H(NH*t*BuBH_3_)(NMe_2_BH_3_)] (**5**)

A mixture of 0.30 g (0.84 mmol) of [Zr(η^5^-C_5_Me_5_)(NMe_2_)_3_], 0.45 g (5.17 mmol) of NH_2_*t*BuBH_3_, and 40 mL of hexane was stirred at room temperature for 16 h in a 100 mL Schlenk tube to give a colorless solution. After filtration, this solution was concentrated in volume to 5 mL and cooled to −35 °C for 3 days to isolate 0.086 g (28%) of colorless crystals, which were characterized as **5**. IR (KBr, cm^–1^): ν̃ 3306 (w) (NH), 2956 (vs), 2907 (vs), 2873 (m), 2836 (m), 2787 (w), 2427 (vs) (BH_term_), 2388 (m) (BH_term_), 2316 (m) (BH_term_), 2058 (s) (BH_bridging_), 1996 (m) (BH_bridging_), 1926 (s) (BH_bridging_), 1891 (m) (BH_bridging_), 1548 (vs) (ZrH), 1461 (m), 1376 (s), 1358 (s), 1292 (m), 1249 (m), 1210 (s), 1173 (s), 1040 (m), 987 (vs), 932 (w), 800 (m), 694 (m), 616 (m), 473 (w), 442 (w). ^1^H NMR (500 MHz, C_6_D_6_, 20 °C): δ 6.91 (s br., 1H; Zr–H), 2.40 (s br., 3H; N*Me*MeBH_3_), 2.33 (s br., 3H; NMe*Me*BH_3_), 1.90 (s, 15H; C_5_Me_5_), 1.31 (s, 9H; CMe_3_); the N*Ht*Bu and B*H*_3_ resonance signals were not observed. ^13^C{^1^H} NMR (125 MHz, C_6_D_6_, 20 °C): δ 119.4 (*C*_5_Me_5_), 54.0 (*C*Me_3_), 52.0 (N*Me*MeBH_3_), 49.9 (NMe*Me*BH_3_), 31.4 (C*Me*_3_), 12.1 (C_5_*Me*_5_). ^11^B NMR (128 MHz, C_6_D_6_, 20 °C): δ 2.7 (q, ^1^*J*(B,H) = 91.8 Hz; NMe_2_BH_3_), −26.6 (q, ^1^*J*(B,H) = 90.8 Hz; NH*t*BuBH_3_). Anal. calcd (%) for C_16_H_38_B_2_N_2_Zr (*M*_w_ = 371.34): C 51.75, H 10.31, N 7.54. Found: C 52.20, H 10.58, N 7.74.

### Synthesis of [Hf(η^5^-C_5_Me_5_)H(NH*t*BuBH_3_)(NMe_2_BH_3_)] (**6**)

Similarly to the preparation of **5**, the treatment of 0.30 g (0.67 mmol) of [Hf(η^5^-C_5_Me_5_)(NMe_2_)_3_] with 0.36 g (4.16 mmol) of NH_2_*t*BuBH_3_ in 40 mL of hexane gave 0.067 g (22%) of colorless crystals of **6**. IR (KBr, cm^–1^): ν̃ 3308 (w) (NH), 2974 (s), 2957 (vs), 2910 (vs), 2875 (s), 2837 (m), 2788 (w), 2454 (vs) (BH_term_), 2435 (vs) (BH_term_), 2391 (s) (BH_term_), 2324 (s) (BH_term_), 2071 (s) (BH_bridging_), 2009 (m) (BH_bridging_), 1941 (s) (BH_bridging_), 1898 (m) (BH_bridging_), 1600 (vs) (HfH), 1463 (s), 1377 (s), 1359 (s), 1304 (s), 1249 (m), 1210 (s), 1175 (vs), 1042 (s), 1005 (vs), 989 (vs), 932 (w), 810 (m), 730 (w), 714 (w), 644 (m), 474 (w), 441 (w). ^1^H NMR (300 MHz, C_6_D_6_, 20 °C): δ 11.74 (s br., 1H; Hf–H), 2.44 (s br., 3H; N*Me*MeBH_3_), 2.40 (s br., 3H; NMe*Me*BH_3_), 1.97 (s, 15H; C_5_Me_5_), 1.30 (s, 9H; CMe_3_); the N*Ht*Bu and B*H*_3_ resonance signals were not observed. ^13^C{^1^H} NMR (75 MHz, C_6_D_6_, 20 °C): δ 117.5 (*C*_5_Me_5_), 54.0 (*C*Me_3_), 52.1 (N*Me*MeBH_3_), 50.1 (NMe*Me*BH_3_), 31.4 (C*Me*_3_), 11.8 (C_5_*Me*_5_). ^11^B NMR (128 MHz, C_6_D_6_, 20 °C): δ 2.7 (q, ^1^*J*(B,H) = 92.7 Hz; NMe_2_BH_3_), −26.2 (q, ^1^*J*(B,H) = 88.4 Hz; NH*t*BuBH_3_). Anal. calcd (%) for C_16_H_38_B_2_HfN_2_ (*M*_w_ = 458.60): C 41.90, H 8.35, N 6.11. Found: C 41.57, H 8.22, N 5.85.

### Synthesis of [Hf(η^5^-C_5_Me_5_)Cl_2_(NMe_2_)]

A mixture of 1.00 g (2.38 mmol) of [Hf(η^5^-C_5_Me_5_)Cl_3_], 0.13 g (2.55 mmol) of [Li(NMe_2_)], 10 mL of toluene, and 50 mL of hexane was stirred at room temperature for 24 h in a 100 mL Schlenk tube. After filtering onto a glass frit and removing the volatile components of the solution, a white solid was obtained. Then, it was extracted with 40 mL of hexane and the resulting colorless solution was filtered and concentrated in volume to 5 mL to give a white precipitate (0.36 g) of [Hf(η^5^-C_5_Me_5_)Cl_2_(NMe_2_)], which was isolated by means of filtration. A second crop (0.20 g) of colorless crystals was isolated by cooling the hexane filtrate at −35 °C for 2 days. The combined yield of [Hf(η^5^-C_5_Me_5_)Cl_2_(NMe_2_)] was 0.56 g (55%). IR (KBr, cm^–1^): ν̃ 3019 (w), 2956 (m), 2914 (vs), 2869 (s), 2785 (m), 1483 (m), 1456 (s), 1430 (s), 1390 (m), 1381 (s), 1261 (s), 1143 (s), 1058 (m), 1027 (m), 912 (vs), 805 (m), 787 (m), 654 (w), 617 (w), 551 (w), 414 (w). ^1^H NMR (500 MHz, C_6_D_6_, 20 °C): δ 2.92 (s, 6H; NMe_2_), 1.92 (s, 15H; C_5_Me_5_). ^13^C{^1^H} NMR (125 MHz, C_6_D_6_, 20 °C): δ 122.1 (*C*_5_Me_5_), 42.2 (NMe_2_), 11.3 (C_5_*Me*_5_). Anal. calcd (%) for C_12_H_21_Cl_2_HfN (*M*_w_ = 428.70): C 33.62, H 4.94, N 3.27. Found: C 33.99, H 4.87, N 3.71.

### Synthesis of [(NHMe_2_)_2_BH_2_][{Hf(η^5^-C_5_Me_5_)Cl_2_}_2_(μ-H)_3_] (**8**)

A mixture of 0.30 g (0.70 mmol) of [Hf(η^5^-C_5_Me_5_)Cl_2_(NMe_2_)], 0.043 g (0.73 mmol) of NHMe_2_BH_3_, and 30 mL of hexane was stirred at room temperature for 24 h in a 100 mL Schlenk tube to give a white solid and a colorless solution. The solid was isolated by filtration onto a glass frit and vacuum-dried to afford **8** (0.20 g, 63%). IR (KBr, cm^–1^): ν̃ 3200 (s) (NH), 3009 (w), 2957 (m), 2909 (s), 2457 (m) (BH), 1480 (vs) (HfH), 1379 (m), 1191 (s), 1031 (w), 916 (w), 875 (w), 824 (m). ^1^H NMR (300 MHz, C_6_D_6_, 20 °C): δ 9.48 (s br., 3H; Hf–H–Hf), 5.23 (s br., 2H; N*H*Me_2_), 2.35 (s, 30H; C_5_Me_5_), 2.03 (d, ^3^*J*(H,H) = 5.4 Hz, 12H; NH*Me*_2_); the B*H*_2_ resonance signal was not observed. ^1^H NMR (300 MHz, CDCl_3_, 20 °C): δ 9.00 (s br., 3H; Hf–H–Hf), 5.39 (s br., 2H; N*H*Me_2_), 2.61 (d, ^3^*J*(H,H) = 5.7 Hz, 12H; NH*Me*_2_), 2.17 (s, 30H; C_5_Me_5_); the B*H*_2_ resonance signal was not observed. ^13^C{^1^H} NMR (75 MHz, CDCl_3_, 20 °C): δ 121.9 (*C*_5_Me_5_), 43.5 (NHMe_2_), 12.3 (C_5_*Me*_5_). ^11^B NMR (128 MHz, CDCl_3_, 20 °C): δ −0.9 (m br., BH_2_). Anal. calcd (%) for C_24_H_49_BCl_4_Hf_2_N_2_ (*M*_w_ = 875.27): C 32.93, H 5.64, N 3.20. Found: C 32.92, H 5.44, N 3.40.

### Synthesis of [(NH_2_*t*Bu)_2_BH_2_][{Hf(η^5^-C_5_Me_5_)Cl_2_}_2_(μ-H)_3_] (**10**)

A mixture of 0.40 g (0.93 mmol) of [Hf(η^5^-C_5_Me_5_)Cl_2_(NMe_2_)], 0.12 g (1.38 mmol) of NH_2_*t*BuBH_3_, and 30 mL of hexane was stirred at room temperature for 24 h in a 100 mL Schlenk tube to give a white solid and a colorless solution. The solid was isolated by means of filtration and washed with hexane (5 mL) to afford **10** (0.33 g, 76%). IR (KBr, cm^–1^): ν̃ 3188 (s) (NH), 3117 (m) (NH), 2977 (m), 2907 (m), 2856 (w), 2486 (m) (BH), 1587 (m) (NH_2_tBu), 1479 (vs) (HfH), 1407 (w), 1377 (s), 1348 (vs), 1201 (vs), 1159 (m), 1029 (m), 886 (m), 821 (vs), 728 (s), 649 (w), 593 (w), 527 (w), 464 (w), 433 (w). ^1^H NMR (400 MHz, C_6_D_6_, 20 °C): δ 8.88 (s br., 3H; Hf–H–Hf), 4.94 (s br., 4H; N*H*_2_CMe_3_), 2.35 (s, 30H; C_5_Me_5_), 0.97 (s, 18H; NH_2_C*Me*_3_); the B*H*_2_ resonance signal was not observed. ^1^H NMR (400 MHz, CDCl_3_, 20 °C): δ 8.66 (s br., 3H; Hf–H–Hf), 4.69 (s br., 4H; N*H*_2_CMe_3_), 2.19 (s, 30H; C_5_Me_5_), 1.29 (s, 18H; NH_2_C*Me*_3_); the B*H*_2_ resonance signal was not observed. ^13^C{^1^H} NMR (100 MHz, C_6_D_6_, 20 °C): δ 122.4 (*C*_5_Me_5_), 54.2 (NH_2_*C*Me_3_), 28.1 (NH_2_C*Me*_3_), 12.7 (C_5_*Me*_5_). ^11^B NMR (128 MHz, C_6_D_6_, 20 °C): δ −13.1 (m br., BH_2_). Anal. calcd (%) for C_28_H_57_BCl_4_Hf_2_N_2_ (*M*_w_ = 931.38): C 36.11, H 6.17, N 3.01. Found: C 36.67, H 5.66, N 2.64.

### Synthesis of [(NH_2_*t*Bu)_2_BH_2_][{Zr(η^5^-C_5_Me_5_)Cl_2_}_2_(μ-H)_3_] (**9**) and [Cl_2_(η^5^-C_5_Me_5_)Zr(μ-Cl)(μ-H)_2_Zr(η^5^-C_5_Me_5_)Cl(NH_2_*t*Bu)] (**11**)

A mixture of 0.40 g (1.22 mmol) of [Zr(η^5^-C_5_Me_5_)Cl_2_(NMe_2_)], 0.11 g (1.26 mmol) of NH_2_*t*BuBH_3_, and 30 mL of hexane was stirred at room temperature for 24 h in a 100 mL Schlenk tube to give a white solid and a colorless solution. The solid was isolated by means of filtration and vacuum-dried to afford **9** (0.24 g, 52%).^33^ From the hexane filtrate, a small fraction of colorless X-ray quality crystals of [Cl_2_(η^5^-C_5_Me_5_)Zr(μ-Cl)(μ-H)_2_Zr(η^5^-C_5_Me_5_)Cl(NH_2_*t*Bu)] (**11**) were grown after 24 h at room temperature.

NMR data for **11**: ^1^H NMR (500 MHz, C_6_D_6_, 20 °C): δ 5.08 (s br., 2H; N*H*_2_CMe_3_), 4.64 (d, ^2^*J*(H,H) = 6.0 Hz, 1H; Zr–H–Zr), 4.50 (d, ^2^*J*(H,H) = 6.0 Hz, 1H; Zr–H–Zr), 2.14 (s, 15H; C_5_Me_5_), 1.87 (s, 15H; C_5_Me_5_), 1.13 (s, 9H; NH_2_C*Me*_3_). ^13^C{^1^H} NMR (125 MHz, C_6_D_6_, 20 °C): δ 126.2 (*C*_5_Me_5_), 125.9 (*C*_5_Me_5_), 53.9 (NH_2_*C*Me_3_), 30.7 (NH_2_C*Me*_3_), 12.6 (C_5_*Me*_5_), 12.2 (C_5_*Me*_5_). Crystals of **11** were contaminated with an unidentified product (≈10% by ^1^H NMR spectroscopy), and the compound could not be further characterized.

### Synthesis of [Cl_2_(η^5^-C_5_Me_5_)Hf(μ-Cl)(μ-H)_2_Hf(η^5^-C_5_Me_5_)Cl(NH_2_*t*Bu)] (**12**)

A mixture of 0.40 g (0.93 mmol) of [Hf(η^5^-C_5_Me_5_)Cl_2_(NMe_2_)], 0.073 g (0.82 mmol) of NH_2_*t*BuBH_3_, and 10 mL of hexane was stirred at room temperature for 2 h in a 100 mL Schlenk tube to give a white solid and a colorless solution. The solid was isolated by means of filtration and vacuum-dried to afford **12** (0.13 g, 37%). IR (KBr, cm^–1^): ν̃ 3294 (w) (NH), 3170 (w) (NH), 2973 (w), 2908 (w), 1563 (w) (NH_2_tBu), 1489 (s) (HfH), 1426 (m), 1399 (m), 1372 (s), 1286 (m), 1196 (w), 1151 (s), 1069 (w), 1026 (m), 928 (w), 896 (m), 875 (s), 820 (m), 782 (w), 731 (m), 620 (m), 596 (w), 419 (m). ^1^H NMR (500 MHz, C_6_D_6_, 20 °C): δ 9.33 (d, ^2^*J*(H,H) = 4.5 Hz, 1H; Hf–H–Hf), 8.96 (d, ^2^*J*(H,H) = 4.5 Hz, 1H; Hf–H–Hf), 4.90 (s br., 2H; N*H*_2_CMe_3_), 2.22 (s, 15H; C_5_Me_5_), 1.95 (s, 15H; C_5_Me_5_), 1.12 (s, 9H; NH_2_C*Me*_3_). ^13^C{^1^H} NMR (125 MHz, C_6_D_6_, 20 °C): δ 123.7 (*C*_5_Me_5_), 123.5 (*C*_5_Me_5_), 54.2 (NH_2_*C*Me_3_), 30.5 (NH_2_C*Me*_3_), 12.3 (C_5_*Me*_5_), 11.9 (C_5_*Me*_5_). Anal. calcd (%) for C_24_H_43_Cl_4_Hf_2_N (*M*_w_ = 844.39): C 34.14, H 5.13, N 1.66. Found: C 34.14, H 5.05, N 1.86.

### Synthesis of [Zr(η^5^-C_5_Me_5_)Cl_2_(NH*t*Bu)] (**13**)

A mixture of 0.20 g (0.29 mmol) of **7**, 0.22 g (2.92 mmol) of NH_2_*t*Bu, and 30 mL of toluene was stirred at 70 °C for 3 days in a 100 mL ampule (Teflon stopcock). After removing the volatile components, the resulting solid was dissolved in 5 mL of hexane and filtered onto a glass frit. This hexane filtrate was cooled at −35 °C, and after 14 days, 0.046 g (22%) of colorless crystals of **13** could be isolated. IR (ATR, cm^–1^): ν̃ 3322 (m) (NH), 2968 (m), 2912 (m), 2869 (m), 1489 (w), 1451 (m), 1426 (m), 1379 (m), 1362 (m), 1332 (m), 1201 (s), 1149 (m), 1027 (m), 944 (s), 917 (m), 772 (s), 634 (m), 601 (vs), 556 (w), 455 (m). ^1^H NMR (500 MHz, C_6_D_6_, 20 °C): δ 6.10 (s br., 1H; N*H*CMe_3_), 1.84 (s, 15H; C_5_Me_5_), 1.30 (s, 9H; NHC*Me*_3_). ^13^C{^1^H} NMR (125 MHz, C_6_D_6_, 20 °C): δ 123.6 (*C*_5_Me_5_), 55.4 (NH*C*Me_3_), 32.6 (NHC*Me*_3_), 11.7 (C_5_*Me*_5_). Anal. calcd (%) for C_14_H_25_Cl_2_NZr (*M*_w_ = 369.49): C 45.51, H 6.82, N 3.79. Found: C 45.32, H 6.84, N 3.98.

### X-ray Crystal Structure Determinations

Colorless crystals of **1** and **12**·0.5C_7_H_8_ were isolated from toluene solutions of the compounds at −35 °C. Colorless crystals of [Hf(η^5^-C_5_Me_5_)(CH_2_CMe_2_Ph)_3_], **4**–**6**, and **13** were grown from hexane solutions at −35 °C. Colorless crystals of **10**·CH_2_Cl_2_ were obtained from a dichloromethane solution at −15 °C. Colorless crystals of **11** were grown from a hexane solution at room temperature. The crystals were removed from the Schlenk tubes and covered with a layer of a viscous perfluoropolyether (FomblinY). A suitable crystal was selected with the aid of a microscope, mounted on a cryoloop, and immediately placed in the low-temperature nitrogen stream of the diffractometer. The intensity data sets were collected at 150 K on a Bruker-Nonius KappaCCD diffractometer (**1**, [Hf(η^5^-C_5_Me_5_)(CH_2_CMe_2_Ph)_3_], **4**–**6**, **11**, and **13**) or on a Bruker AXS D8 Venture diffractometer (**10** and **12**). Both diffractometers were equipped with an Oxford Cryostream 700 unit.

Crystallographic data for complexes are presented in Tables S1 and S2. The structures were solved, using the WINGX package,^38^ by direct methods (**1**, **4**, and **13**) (SHELXS-2013)^39^ or intrinsic phasing methods (the rest) (SHELXT)^40^ and refined by least-squares against F^2^ (SHELXL-2014/7).^39^ Crystals of **1** showed disorder for atoms N(3) and B(3) of one amidoborane ligand. This disorder was conventionally treated by using the PART tool and allowing free refinement of the occupancy factors with the FVAR command of the SHELXL program. The final values were 50% for both positions. All non-hydrogen atoms were anisotropically refined. Hydrogen atoms of methyl fragments and the disordered amidoborane group were positioned geometrically and refined using a riding model. The FREE instruction was applied to the atoms N(3), B(3), N(3)’, and B(3)’ to avoid problems on these calculated hydrogen atoms. On the other hand, hydrogen atoms of nondisordered amidoborane ligands (bound to N(1), N(2), B(1), and B(2)) were located in the difference Fourier map and isotropically refined, except H(2)c whose isotropic displacement parameter after the last refinement cycles was not appropriate, so its *U*_iso_ was forced to be 0.05. Moreover, DELU restraints were applied on B(3)’ and N(3)’ atoms.

In the crystallographic studies of [Hf(η^5^-C_5_Me_5_)(CH_2_CMe_2_Ph)_3_] and **4**–**6**, all non-hydrogen atoms were anisotropically refined. All hydrogen atoms were positioned geometrically and refined by using a riding model, except those bound to boron, zirconium, or hafnium atoms, which were found in the difference Fourier map and refined isotropically.

Compound **10** crystallized with a molecule of dichloromethane in the *Fddd* space group. All of the non-hydrogen atoms were anisotropically refined. The hydrogen atoms bound to B(1) (H(10)) and Hf(1) (H(1) and H(2)) were located in the difference Fourier map and refined isotropically. The rest of the hydrogen atoms were positioned geometrically and refined by using a riding model.

Crystals of **11** contained two independent molecules in the asymmetric unit with no substantial differences. Moreover, these crystals presented disorder for the carbon atoms of the pentamethylcyclopentadienyl ligand linked to Zr(4) (C(41)–C(50)), which was also treated conventionally, affording occupancy factors of 56 and 44%. All non-hydrogen atoms were anisotropically refined, while all of the hydrogen atoms were positioned geometrically and refined using a riding model, except those bridging the zirconium atoms (H(1), H(2), H(3), and H(4)). These hydrogen atoms were located in the Fourier map and isotropically refined. Additionally, DELU and SIMU restraints were employed with carbon atoms of the disordered C_5_Me_5_ group.

Complex **12** crystallized with a half-molecule of toluene, which was found in the difference Fourier map. However, it was not possible to obtain a chemically sensible model for it, so Squeeze^41^ procedure was used to remove its contribution to the structure factors. Furthermore, this crystal showed disorder for the carbon atoms C(21)–C(30) of the pentamethylcyclopentadienyl ring linked to Hf(2). This disorder was also treated by using the PART tool with final values of 79 and 21%. All non-hydrogen atoms were anisotropically refined, except the carbon atoms related to the minor position of the disordered C_5_Me_5_ ligand (C(21)’–C(30)’), which were isotropically refined. The hydrogen atoms of the methyl groups were positioned geometrically and refined by using a riding model. Additionally, the XHYDEX tool was employed to locate hydride groups bound to the hafnium atoms. Thus, these hydrogen atoms, namely, H(1) and H(2), and also those linked to nitrogen (H(1)a and H(1)b), were found in the difference Fourier map and refined isotropically. Moreover, C(21)–C(30) atoms of the disordered ring were restrained with DELU and SIMU commands, whereas carbon atoms C(21)’–C(30)’ of the minor position for the disordered C_5_Me_5_ moiety were restrained with SADI instructions. SADI restraints were also applied to the distances N–H.

Finally, in the crystallographic study of **13**, all non-hydrogen atoms were anisotropically refined. The hydrogen atoms of methyl groups were placed geometrically and refined using a riding model. The hydrogen atom bound to nitrogen (H(1)) was found in the Fourier map and isotropically refined.

## Results and Discussion

### Reactions of Trialkyl and Tris(dialkylamido) Complexes with Amine–Boranes

**Scheme 1:**
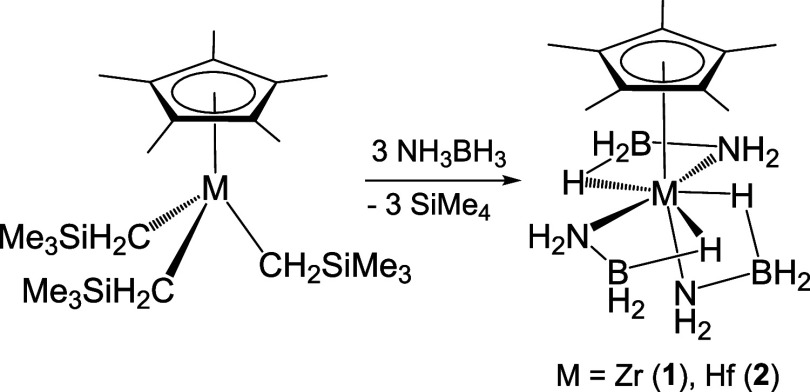
Reactions of [M(η^5^-C_5_Me_5_)(CH_2_SiMe_3_)_3_] with NH_3_BH_3_

The treatment of [M(η^5^-C_5_Me_5_)(CH_2_SiMe_3_)_3_] (M = Zr, Hf) with 3 equiv of ammonia–borane (NH_3_BH_3_) afforded the tris(amidoborane) complexes [M(η^5^-C_5_Me_5_)(NH_2_BH_3_)_3_] (M = Zr (**1**), Hf (**2**)) (Scheme 1). The zirconium compound **1** was readily prepared in toluene at room temperature. However, the synthesis of the analogue hafnium species **2** required heating at 45 °C in tetrahydrofuran for the complete consumption of the trialkyl precursor. Complexes **1** and **2** were isolated in 73 and 46% yields as white solids that exhibit good solubility in benzene and toluene but poorly dissolve in hexane. The compounds displayed significant thermal stability in benzene-*d*_6_ solutions but slowly decomposed at temperatures higher than 60 °C to give dihydrogen (δ = 4.46 ppm) along with a complicated mixture of products according to ^1^H NMR spectroscopy. Noteworthily, we have previously reported that the analogous treatment of complexes [M(η^5^-C_5_Me_5_)(CH_2_SiMe_3_)_3_] with the bulkier *N,N*-dimethylamine–borane (3 equiv) occasioned the release of one equivalent of SiMe_4_ and the formation of the thermally unstable mono(amidoborane) derivatives [M(η^5^-C_5_Me_5_)(CH_2_SiMe_3_)_2_(NMe_2_BH_3_)] (M = Zr, Hf).^31^

**Figure 1:**
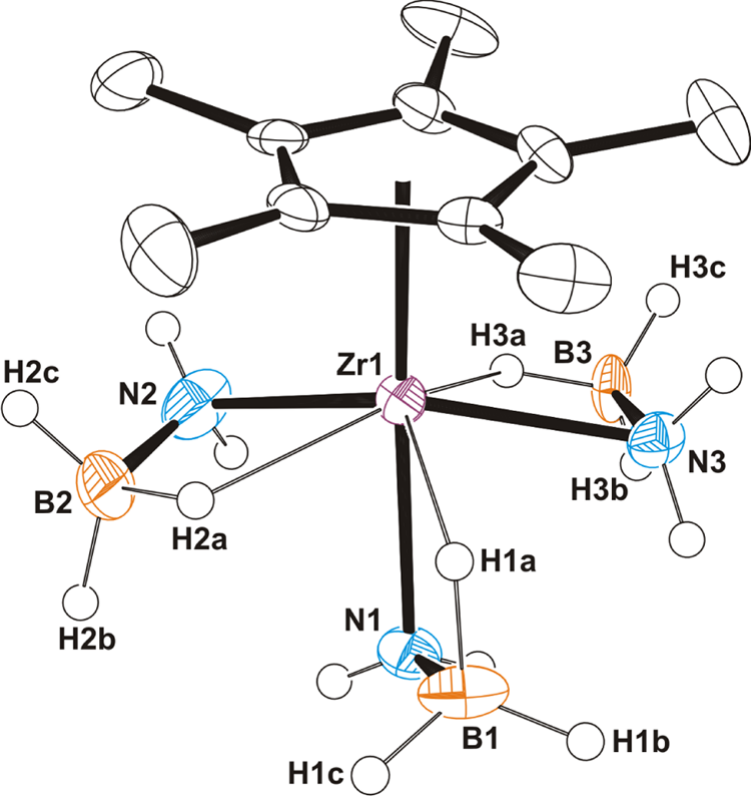
ORTEP diagram (50% probability level) of **1**. Hydrogen atoms of methyl groups are omitted for clarity. Selected lengths (Å) and angles (deg): Zr(1)–N(1) 2.276(5), Zr(1)–N(2) 2.240(5), Zr(1)–N(3) 2.291(9), Zr(1)···B(1) 2.665(8), Zr(1)···B(2) 2.664(8), Zr(1)···B(3) 2.70(4), N(1)–Zr(1)–N(2) 83.4(2), N(1)–Zr(1)–N(3) 77.5(3), N(2)–Zr(1)–N(3) 135.6(3), Zr(1)–N(1)–B(1) 86.5(3), Zr(1)–N(2)–B(2) 87.5(4), and Zr(1)–N(3)–B(3) 85.8(10).

Colorless single crystals of the zirconium compound **1** for an X-ray diffraction analysis were obtained from a toluene solution at −35 °C (Figure 1). The crystal structure shows three amidoborane ligands acting in a bidentate fashion through the nitrogen atom and a single hydride of the BH_3_ group. If the centroid of the η^5^-C_5_Me_5_ ligand is considered, the coordination environment around the zirconium atom could be described as distorted pentagonal bipyramidal. In this geometry, the N(1) atom and the pentamethylcyclopentadienyl ring are at the axial sites, while the remaining two nitrogen atoms (N(2) and N(3)) along with H(1)a, H(2)a, and H(3)a hydrogen atoms occupy the equatorial sites. The Zr–N bond lengths vary from 2.240(5) to 2.291(9) Å, being in the usual range for zirconium–nitrogen distances in related κ^2^*N,H*-amidoborane complexes.^7,24,26b,33^ The zirconium···boron distances of av. 2.68(2) Å and the Zr–N–B angles of av. 86.6(9)° in complex **1** are consistent with the Zr···H–B interactions and are compared well with those found in complexes [Zr(η^5^-C_5_H_5_)_2_(NH_2_BH_3_)X] containing κ^2^*N,H*-NH_2_BH_3_ ligands.^24^ An X-ray powder diffraction study for compound **1** was performed to confirm that the single-crystal structure is representative of the bulk sample. The experimental diffractogram of the crystalline material is similar to that calculated in the single-crystal analysis (Figures S1 and S2). Therefore, the crystal structure shown in Figure 1 seems to represent the overall material of **1**, although the presence of amorphous species cannot be ruled out.

The IR (KBr) spectra of derivatives **1** and **2** show two strong absorptions in the ν_NH_ region, between 3409 and 3328 cm^–1^, for the amidoborane NH_2_BH_3_ ligands.^24,25,28^ In addition, the IR spectra reveal strong bands in the range from 2415 to 2328 cm^–1^ and several absorptions between 1936 and 1778 cm^–1^, which are characteristics of the terminal and bridging B–H bonds, respectively. The ^1^H NMR spectra of compounds **1** and **2** in benzene-d_6_ at room temperature display one sharp singlet for the η^5^-C_5_Me_5_ ligands and multiple very broad resonances between 1.50 and 0.00 ppm for the three amidoborane NH_2_BH_3_ ligands. The ^1^H{^11^B} NMR spectra also reveal broad resonances, indicating that the signals corresponding to the NH_2_ and BH_3_ protons of inequivalent NH_2_BH_3_ groups overlap in the range of 1.50–0.00 ppm. While the ^11^B NMR spectra at room temperature feature broad multiplets in the range from −18 to −28 ppm, the ^11^B{^1^H} NMR spectra reveal three broad singlet resonances for inequivalent BH_3_ groups. These data suggest a dynamic behavior in solution involving slow exchange of the amidoborane ligands on the NMR time scale. Indeed, the ^11^B{^1^H} NMR spectra of complexes **1** and **2** in benzene-*d*_6_ at 60 °C display one broad singlet. In addition to this site interconversion of the amidoborane groups at the seven-coordinate complexes, an exchange of BH_3_ resonances within each NH_2_BH_3_ ligand, similar to that studied by Roesler in complex [Zr(η^5^-C_5_H_5_)_2_H(NH_2_BH_3_)],^24^ could explain the complicated ^1^H and ^1^H{^11^B} NMR spectra of complexes **1** and **2**.

**Scheme 2:**
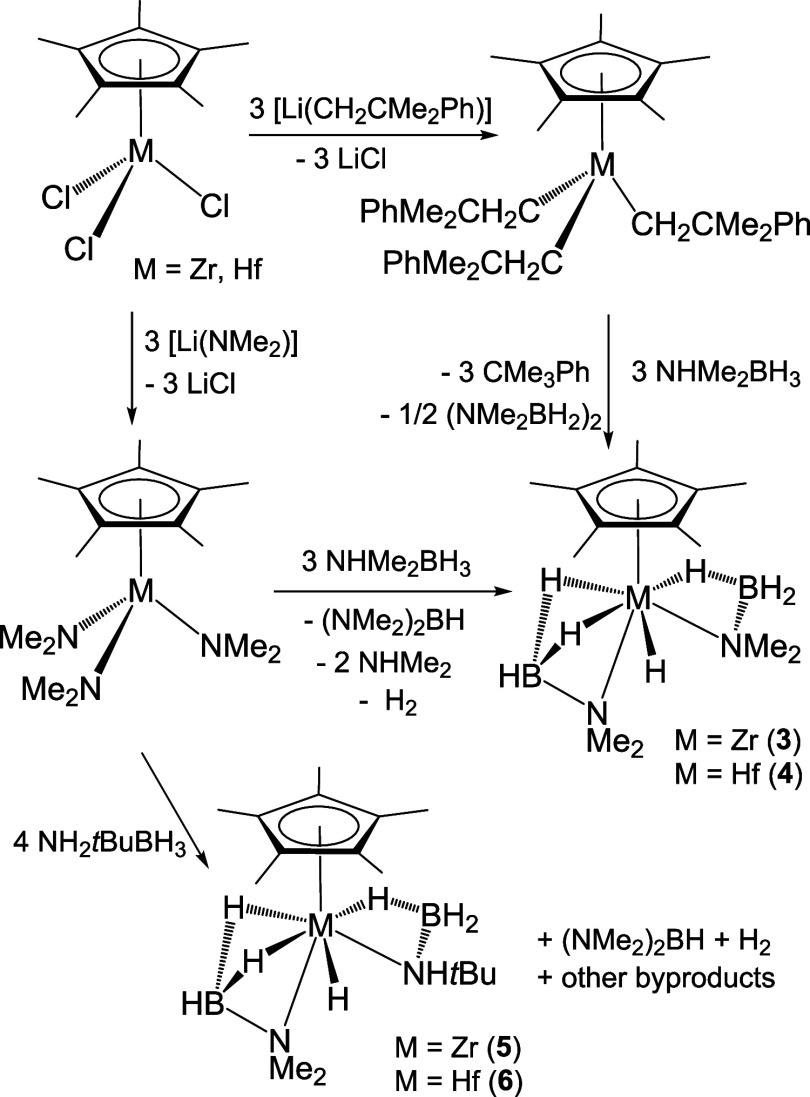
Reactions of Tris(neophyl) and Tris(dimethylamido) Complexes with NHR_2_BH_3_ (R_2_ = Me_2_, H*t*Bu)

We have also studied the reaction of the half-sandwich tris(neophyl) and tris(dimethylamido) zirconium and hafnium derivatives with *N,N*-dimethylamine–borane (Scheme 2). The tris(amido) precursors were prepared according to reported procedures,^36,37^ while complexes [M(η^5^-C_5_Me_5_)(CH_2_CMe_2_Ph)_3_] (M = Zr, Hf) were obtained similarly through the treatment of the trihalides [M(η^5^-C_5_Me_5_)Cl_3_] with 3 equiv of [Li(CH_2_CMe_2_Ph)] in hexane at room temperature. The hitherto unknown trialkyl derivatives [M(η^5^-C_5_Me_5_)(CH_2_CMe_2_Ph)_3_] were characterized by spectroscopic and analytical methods. The crystal structure of the hafnium complex was also determined by a single-crystal diffraction experiment. The molecular structure of [Hf(η^5^-C_5_Me_5_)(CH_2_CMe_2_Ph)_3_] exhibits the usual three-legged piano-stool arrangement found in group 4 half-sandwich complexes (Figure S3).

Treatment of the tris(neophyl) complexes [M(η^5^-C_5_Me_5_)(CH_2_CMe_2_Ph)_3_] with *N*,*N*-dimethylamine–borane (3 equiv) in toluene at room temperature led to the bis(dimethylamidoborane) hydride derivatives [M(η^5^-C_5_Me_5_)H(NMe_2_BH_3_)_2_] (M = Zr (**3**), Hf (**4**)) (Scheme 2). However, compounds **3** and **4** were always isolated with significant amounts of the alkane CMe_3_Ph, which is not volatile and has a solubility in usual organic solvents similar to that of the group 4 compounds precluding purification by fractional crystallization. Fortunately, the reaction of the tris(dimethylamido) derivatives [M(η^5^-C_5_Me_5_)(NMe_2_)_3_] with 6 equiv of NHMe_2_BH_3_ in hexane at room temperature produced complexes **3** and **4** in a pure form and good yield (80 and 52%, respectively). The addition of stoichiometric 3 equiv of NHMe_2_BH_3_ on the tris(amido) reagents gave complexes **3** and **4** in lower yields, presumably by the formation of other metal complexes as byproducts. In particular, the treatment of the zirconium tris(dimethylamido) complex with 3 equiv of NHMe_2_BH_3_ allowed the isolation of the trinuclear polyhydride [{Zr(η^5^-C_5_Me_5_)}_3_(μ_3_-H)(μ-H)_3_(μ-CH_2_NMe)_2_(NMe_2_BH_3_)].^33^ In addition, the reactions of the tris(dimethylamido) derivatives [M(η^5^-C_5_Me_5_)(NMe_2_)_3_] with *N*-*tert*-butylamine–borane (≥4 equiv) in hexane at room temperature afforded colorless solutions from which crystals of mixed bis(amidoborane) hydride complexes [M(η^5^-C_5_Me_5_)H(NH*t*BuBH_3_)(NMe_2_BH_3_)] (M = Zr (**5**), Hf (**6**)) were grown at −35 °C.

**Figure 2:**
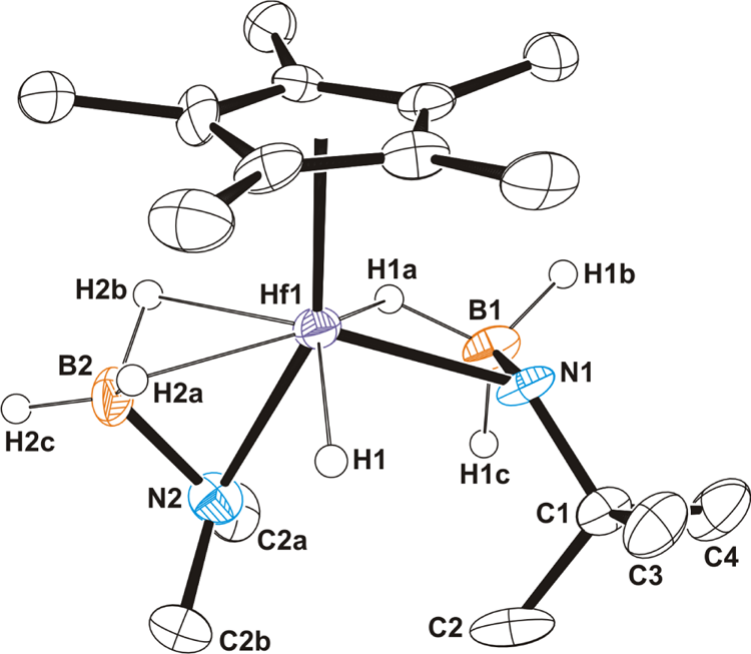
ORTEP diagram (50% probability level) of **6**. Hydrogen atoms bound to nitrogen and carbon atoms are omitted for clarity.

Compounds **3**–**6** were characterized by spectroscopic and analytical methods, and single-crystal X-ray diffraction analysis. Hafnium complexes **4** (Figure S4) and **6** (Figure 2) are isostructural with the analogous zirconium derivatives **3**(33) and **5** (Figure S5). Selected distances and angles of the four structures are compared in Table 1. Each molecular structure shows two chelating amidoborane ligands, which exhibit different coordination modes, namely, κ^2^*N,H*-NR_2_BH_3_ (R_2_= Me_2_ or H*t*Bu)^32^ and κ^3^*N,H,H*-NMe_2_BH_3_. One terminal hydrogen atom and the pentamethylcyclopentadienyl ligand complete the coordination sphere of the group 4 atom, so, if the centroid of the η^5^-C_5_Me_5_ is considered, a seven-coordinate geometry about the metal center can be defined. The κ^2^*N,H*-NR_2_BH_3_ ligand binds to the metal with M–N bond lengths (2.242(8)–2.306(3) Å) shorter than those associated with the κ^3^*N,H,H*-NMe_2_BH_3_ groups (2.336(4)–2.370(4) Å).^19^ The M···B separations (2.651(12)–2.680(5) Å) taking part in M···H–B interactions in the κ^2^*N,H*-NR_2_BH_3_ ligands are longer than those determined for the κ^3^*N,H,H*-NMe_2_BH_3_ moieties (2.344(6)–2.381(7) Å), which exhibit two M···H–B interactions with the same metal center. The M(1)–H(1) bond lengths are in the typical range for terminal hydride ligands bonded to those metals.^42,43^

**Table 1:** Selected Data for Complexes [M(η^5^-C_5_Me_5_)H(NMe_2_BH_3_)(NR_2_BH_3_)] (**3**–**6**)

	M = Zr, R_2_ = Me_2_ (**3**)	M = Hf, R_2_ = Me_2_ (**4**)	M = Zr, R_2_ = H*t*Bu (**5**)	M = Hf, R_2_ = H*t*Bu (**6**)
Lengths (Å) and Angles (deg) in the X-ray Crystal Structures				
M(1)–N(1)	2.306(3)	2.277(4)	2.282(4)	2.242(8)
M(1)–N(2)	2.353(3)	2.336(4)	2.370(4)	2.341(8)
M(1)–H(1)	1.83(4)	1.86(6)	1.87(6)	1.92(11)
M(1)···B(1)	2.680(5)	2.656(7)	2.669(6)	2.651(12)
M(1)···B(2)	2.359(5)	2.344(6)	2.381(7)	2.364(11)
N(1)–M(1)–N(2)	90.5(1)	90.0(2)	99.8(2)	99.0(3)
M(1)–N(1)–B(1)	85.6(2)	85.5(3)	86.1(3)	86.5(6)
M(1)–N(2)–B(2)	71.3(2)	71.2(3)	71.6(3)	71.8(5)
IR (KBr, cm^–1^)				
ν(M–H)	1560	1611	1548	1600
ν(N–H)			3306	3308
^1^H NMR (C_6_D_6_, 20 °C, δ)				
M–H	6.76	11.39	6.91	11.74
BH_3_ (^1^*J*_HB_ in Hz)	0.76 (88.5)	1.32 (90.6)	not observed	not observed
^11^B NMR (C_6_D_6_, 20 °C, δ)				
BH_3_ (^1^*J*_BH_ in Hz)	–7.3 (89.7)	–7.3 (br.)	2.7 (91.8), –26.6 (90.8)	2.7 (92.7), –26.2 (88.4)

The IR spectra (KBr) of complexes **3**–**6** reveal one strong band for the ν_MH_ stretching vibrations between 1548 and 1611 cm^–1^ (Table 1).^24,42,43^ The ^1^H NMR spectra of **3**–**6** in benzene-d_6_ at room temperature exhibit sharp singlets assigned to the η^5^-C_5_Me_5_ rings and one low-field broad resonance for the hydride ligands at chemical shifts (Table 1) similar to those found in other zirconium and hafnium complexes with terminal M–H groups.^42,43^ Additionally, the ^11^B NMR spectra of **3** and **4** display one resonance signal at δ = −7.3 ppm, indicating that the dimethylamidoborane ligands are equivalent in solution, whereas two quartet resonances were observed in the ^11^B NMR spectra of **5** and **6** in agreement with the equivalence of all hydrogen atoms in each BH_3_ group on the NMR time scale. According to HMBC ^1^H–^11^B NMR experiments on solutions of **5** and **6**, the well-defined downfield signals at δ = 2.7 ppm correspond to the NMe_2_BH_3_ ligands, whereas the broader quartet resonances at δ = −26.6 and −26.2 ppm are due to the NH*t*BuBH_3_ moieties.

To gain insight into the mechanism of the formation of complexes **3**–**6**, several NMR tube experiments have been performed. Thus, the reaction of [Zr(η^5^-C_5_Me_5_)(CH_2_CMe_2_Ph)_3_] with 3 equiv of NHMe_2_BH_3_ in benzene-d_6_ at room temperature was examined by ^1^H and ^11^B NMR spectroscopies. Total consumption of the reagents was observed after 24 h, and the spectra revealed the clean formation of complex **3** along with the byproducts CMe_3_Ph and (NMe_2_BH_2_)_2_. The observed byproducts are consistent with the complete substitution of the neophyl ligands in the trialkyl precursor to give the alkane CMe_3_Ph and the tris(dimethylamidoborane) complex [Zr(η^5^-C_5_Me_5_)(NMe_2_BH_3_)_3_] similar to the isolated compounds **1** and **2**. However, this sterically encumbered intermediate was not detected in the NMR spectra because it readily undergoes a β-hydride elimination in one amidoborane ligand in order to generate the bis(dimethylamidoborane) hydride complex **3** along with dimethylaminoborane, which dimerizes to the cyclic compound (NMe_2_BH_2_)_2_. Similarly, spectra taken on the reaction of the zirconium tris(dimethylamido) derivative with NHMe_2_BH_3_ in benzene-d_6_ showed complete consumption of the reagents after 5 min at room temperature. The spectra revealed resonance signals for **3** along with those for NHMe_2_, H_2_, and (NMe_2_)_2_BH. The observed byproducts are also consistent with the formation of the [Zr(η^5^-C_5_Me_5_)(NMe_2_BH_3_)_3_] intermediate, which readily decomposes to form complex **3** and dimethylaminoborane. Nevertheless, on this occasion, we could not detect the cyclic dimer (NMe_2_BH_2_)_2_ in the NMR spectra, and the observed bis(dimethylamino)borane and H_2_ byproducts probably resulted from the reaction of NHMe_2_BH_3_ and dimethylamine.^44^

**Scheme 3:**
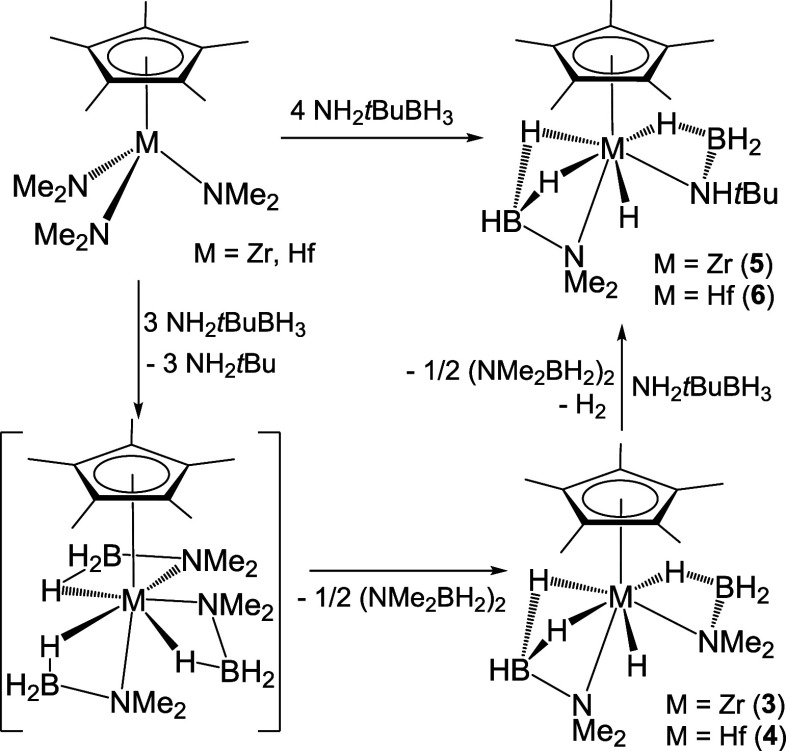
Plausible Proposal for the Formation of Complexes **5** and **6**

On the other hand, while complexes **5** and **6** were isolated in low yields (28 and 22%, respectively) after crystallization in hexane, analysis of the crude product of the reaction [M(η^5^-C_5_Me_5_)(NMe_2_)_3_] with NH_2_*t*BuBH_3_ in toluene by ^1^H NMR spectroscopy in benzene-d_6_ revealed the presence of other minor byproducts. The reaction course of [Zr(η^5^-C_5_Me_5_)(NMe_2_)_3_] with 3 equiv of NH_2_*t*BuBH_3_ in benzene-d_6_ at room temperature was monitored by ^1^H NMR spectroscopy. After 10 min, the spectrum revealed a complicated mixture of products in solution. This mixture includes the zirconium compounds **3** and **5**, H_2_, (NMe_2_)_2_BH, NHMe_2_, NH_2_*t*Bu, and other unidentified products. Spectra taken after 16 h showed the complete consumption of compound **3** and the amines NHMe_2_ and NH_2_*t*Bu. Thus, the potential transformation of the bis(dimethylamidoborane) hydride derivative **3** to complex **5** in benzene-d_6_ was inspected by a few NMR tube experiments at room temperature. Treatment of complex **3** with *tert*-butylamine affords a complicated mixture of products, but resonances for **5** were not detected in the ^1^H NMR spectra. However, complex **3** cleanly reacted with one equivalent of NH_2_*t*BuBH_3_ to give compound **5** along with (NMe_2_BH_2_)_2_ and H_2_ after 24 h at room temperature. A plausible pathway that can explain the formation of complexes [M(η^5^-C_5_Me_5_)H(NH*t*BuBH_3_)(NMe_2_BH_3_)] is shown in Scheme 3. In this proposal, the tris(dimethyl)amido precursors react with 3 equiv of *N*-*tert*-butylamine–borane to give NH_2_*t*Bu and the tris(dimethylamidoborane) [M(η^5^-C_5_Me_5_)(NMe_2_BH_3_)_3_] intermediates. As mentioned above, these intermediates are thermally unstable and undergo rapid β-hydride elimination in one amidoborane ligand to form complexes **3** and **4**. These compounds could subsequently react with one equivalent of NH_2_*t*BuBH_3_ to give complexes **5** and **6** along with NHMe_2_BH_3_, which suffer dehydrogenation to produce the observed cyclic dimer (NMe_2_BH_2_)_2_ and H_2_.

### Reactions of Monoamido Complexes with Amine–Boranes

**Scheme 4:**
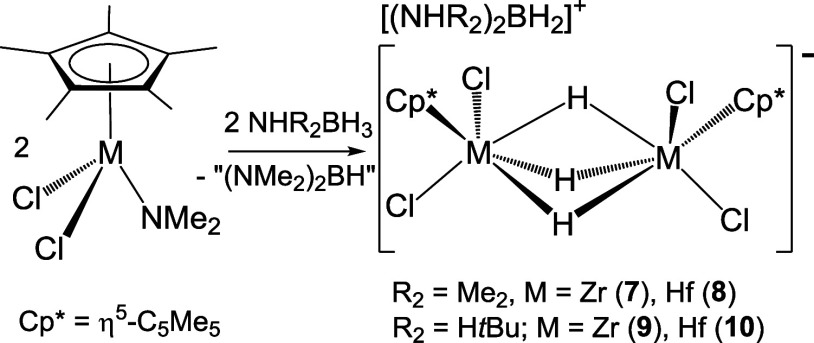
Synthesis of Ionic Compounds **7**–**10**

The addition of NHR_2_BH_3_ on the zirconium and hafnium mono(dimethylamido) derivatives [M(η^5^-C_5_Me_5_)Cl_2_(NMe_2_)] in hexane at room temperature produced the ionic compounds [(NHR_2_)_2_BH_2_][{M(η^5^-C_5_Me_5_)Cl_2_}_2_(μ-H)_3_] (R_2_ = Me_2_, M = Zr (**7**), Hf (**8**); R_2_ = H*t*Bu, M = Zr (**9**), Hf (**10**)) as white precipitates (Scheme 4). Whereas compounds **7**–**9** were obtained in high purity from the reactions with one equivalent of NHR_2_BH_3_, the synthesis of the analogue hafnium compound **10** required a higher ratio (1.5 equiv) of NH_2_*t*BuBH_3_. Compounds **7**–**10** were isolated in 52–76% yields as white powders, which dissolve in chlorinated and aromatic hydrocarbon solvents. However, according to ^1^H NMR spectroscopy, they slowly decompose in solution.

**Figure 3:**
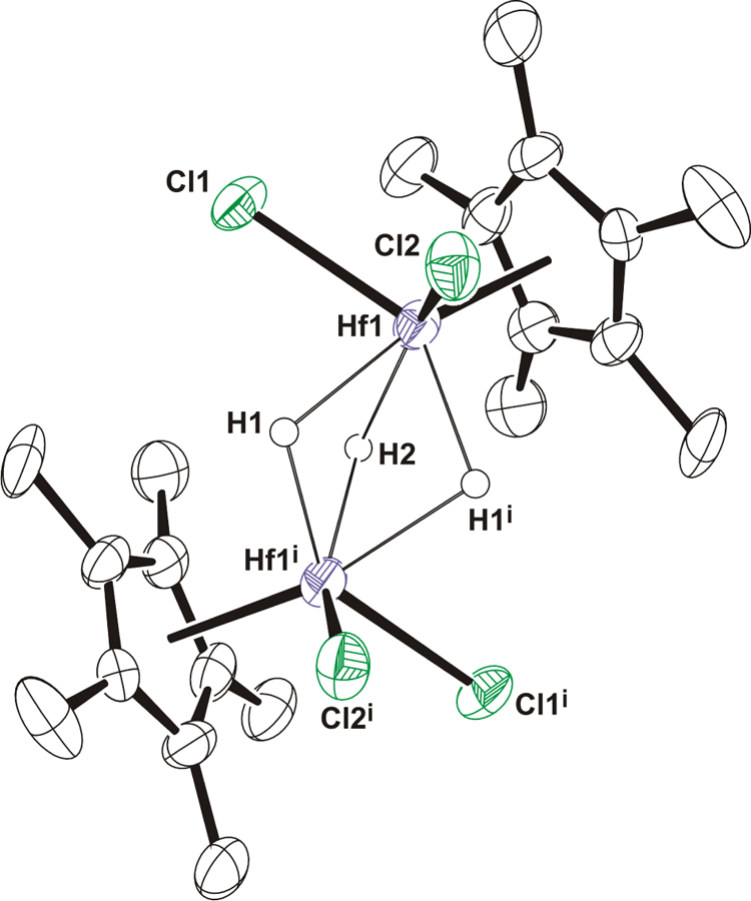
ORTEP diagram (50% probability level) of the anionic fragment of **10**. Hydrogen atoms of the methyl groups are omitted for clarity. Symmetry code: (i) −*x* + 7/4, −*y* + 3/4, *z*.

Complexes **7**–**10** were characterized by analytical and spectroscopic methods. In addition, crystal structures of **9**·CH_2_Cl_2_ and **10**·CH_2_Cl_2_ were determined by X-ray diffraction analysis on suitable crystals obtained from dichloromethane solutions at −15 °C. Hafnium complex **10** (Figure S6) is isostructural with the reported zirconium derivative **9**,^33^ and selected distances and angles for both are compared in Table 2. The solid-state structures contain dimetallic trihydride anionic moieties [{M(η^5^-C_5_Me_5_)Cl_2_}_2_(μ-H)_3_]^−^ and boronium cations [(NH_2_*t*Bu)_2_BH_2_]^+^ associated by means of N–H···Cl hydrogen bonds (Figure S7 and Table S4). The crystal structure for the anion in **10** is presented in Figure 3. In the anionic fragments of **9** and **10**, two {M(η^5^-C_5_Me_5_)Cl_2_} moieties are linked by three bridging hydrogen atoms, generating a distorted octahedral geometry about each zirconium or hafnium atom if the centroid of the η^5^-C_5_Me_5_ is considered. The M···M separations are comparable to those found for other dinuclear zirconium and hafnium derivatives that exhibit bridging hydride groups.^45^

**Table 2:** Selected Data for Complexes [(NHR_2_)_2_BH_2_][{M(η^5^-C_5_Me_5_)Cl_2_}_2_(μ-H)_3_] (**7**–**10**)

	M = Zr, R_2_ = Me_2_ (**7**)	M = Hf, R_2_ = Me_2_ (**8**)	M = Zr, R_2_ = H*t*Bu (**9**)	M = Hf, R_2_ = H*t*Bu (**10**)
Lengths (Å) and Angles (deg) in the X-ray Crystal Structures				
B(1)–N(1)			1.595(5)	1.624(19)
M(1)–H			1.80(4)–1.91(3)	1.86(14)–2.13(12)
M(1)–Cl(1)			2.470(1)	2.448(4)
M(1)–Cl(2)			2.458(2)	2.429(4)
M(1)···M(1)			3.106(3)	3.048(1)
N(1)–B(1)–N(1)			105.6(4)	104.3(17)
Cl(1)–M(1)–Cl(2)			98.8(1)	97.6(1)
H–M(1)–H			63(2)	63(5)–67(3)
IR (KBr, cm^–1^)				
ν(M–H–M)	1457	1480	1463	1479
ν(N–H)	3188	3200	3191, 3114	3188, 3117
ν(B–H)	2456	2457	2484	2486
^1^H NMR (CDCl_3_, 20 °C, δ)				
M–H–M	3.96 (s, br.)	9.00 (s, br.)	3.84 (s, br.)	8.66 (s, br.)
NHR_2_ (^3^*J*_HH_ in Hz)	5.46 (s, br.), 2.61 (d, 6.0)	5.39 (s, br.), 2.61 (d, 5.7)	4.76 (s, br.), 1.28 (s)	4.69 (s, br.), 1.29 (s)
^11^B NMR (CDCl_3_ or C_6_D_6_^a^, 20 °C, δ)				
[NHR_2_BH_2_]^+^	–0.7 (br.)	–0.9 (br.)	–13.1 (br.)	–13.1 (br.)^a^

The IR spectra (KBr) of complexes **7**–**10** show absorptions for the ν_NH_ and ν_BH_ vibrations of the cations [(NHR_2_)_2_BH_2_]^+^.^46^ In addition, one very strong band in the IR spectra, between 1480 and 1457 cm^–1^, is tentatively attributed to the M–H stretching of the M–H–M moieties.^45^ The ^1^H NMR spectra of complexes **7**–**10** in chloroform-d_1_ exhibit one sharp singlet for the pentamethylcyclopentadienyl ligands and one broad signal for the three bridging hydride groups of the [{M(η^5^-C_5_Me_5_)Cl_2_}_2_(μ-H)_3_]^−^ anion. Additionally, these spectra display the expectable resonance signals assignable to the NH and CH_3_ protons of the boronium cations.^46a,47^ Furthermore, the ^11^B NMR spectra in chloroform-d_1_ or benzene-d_6_ show broad resonance signals, which are comparable with those determined for other salts containing the same cationic fragments.^47b,48^

**Scheme 5:**
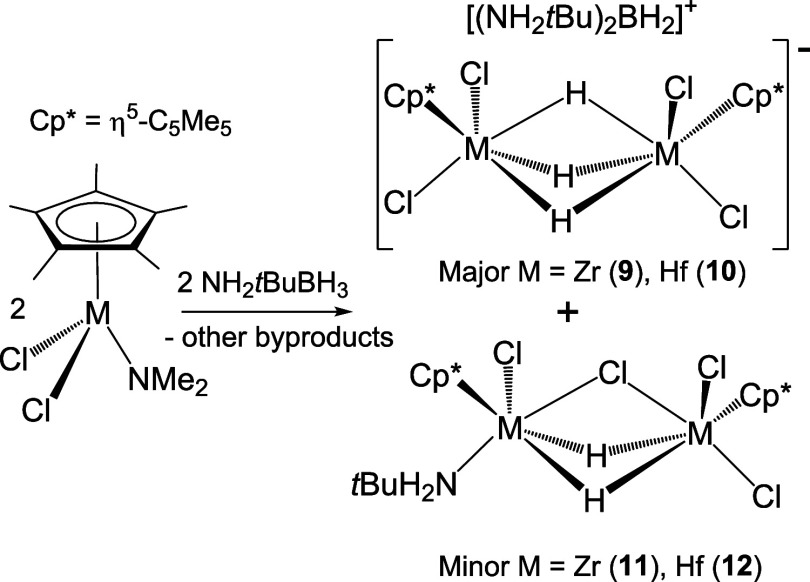
Synthesis of Complexes **11** and **12**

While compounds **7**–**10** could be isolated in a pure form due to their poor solubility in hexane, other minor group 4 byproducts remained in solution. For instance, from the hexane solution obtained in the synthesis of **9** by the treatment of [Zr(η^5^-C_5_Me_5_)Cl_2_(NMe_2_)] with NH_2_*t*BuBH_3_, a small fraction of X-ray quality crystals of the dinuclear complex [Cl_2_(η^5^-C_5_Me_5_)Zr(μ-Cl)(μ-H)_2_Zr(η^5^-C_5_Me_5_)Cl(NH_2_*t*Bu)] (**11**) was produced (Scheme 5). The ^1^H and ^13^C{^1^H} NMR spectra of the isolated colorless crystals of **11** also reveal the presence of an unidentified compound in solution (≈ 10% by ^1^H NMR spectroscopy), precluding the characterization of **11** by other techniques. Fortunately, the treatment of a concentrated hexane solution of [Hf(η^5^-C_5_Me_5_)Cl_2_(NMe_2_)] with 0.9 equiv of NH_2_*t*BuBH_3_ for 2 h led to the precipitation of the hafnium analogue [Cl_2_(η^5^-C_5_Me_5_)Hf(μ-Cl)(μ-H)_2_Hf(η^5^-C_5_Me_5_)Cl(NH_2_*t*Bu)] (**12**) in a pure form, although in low yield (37%). Thus, complex **12** was characterized by analytical and spectroscopic methods. Moreover, colorless crystals of **12**·0.5C_7_H_8_, isolated from a toluene solution at −35 °C, were suitable for an X-ray diffraction analysis. The ^1^H and ^13^C{^1^H} NMR spectra of **11** and **12** in benzene-d_6_ at room temperature show resonances attributable to two inequivalent pentamethylcyclopentadienyl groups and those assignable to one *tert*-butylamine ligand per dinuclear complex. In addition, the ^1^H NMR spectra display two doublets at δ = 4.64 (^2^*J*(H,H) = 6.0 Hz) and 4.50 ppm (^2^*J*(H,H) = 6.0 Hz) for **11** and at δ = 9.33 (^2^*J*(H,H) = 4.5 Hz) and 8.96 ppm (^2^*J*(H,H) = 4.5 Hz) for **12** attributable to two nonequivalent bridging hydride ligands. The IR spectrum (KBr) of **12** reveals two weak absorptions for ν_NH_ vibrations of the *tert*-butylamine ligand at 3294 and 3170 cm^–1^ and one band assignable to the NH_2_*t*Bu bending mode at 1563 cm^–1^. The spectrum also shows one strong absorption for the Hf–H stretching of the Hf–H–Hf fragments at 1489 cm^–1^.^45^

**Figure 4:**
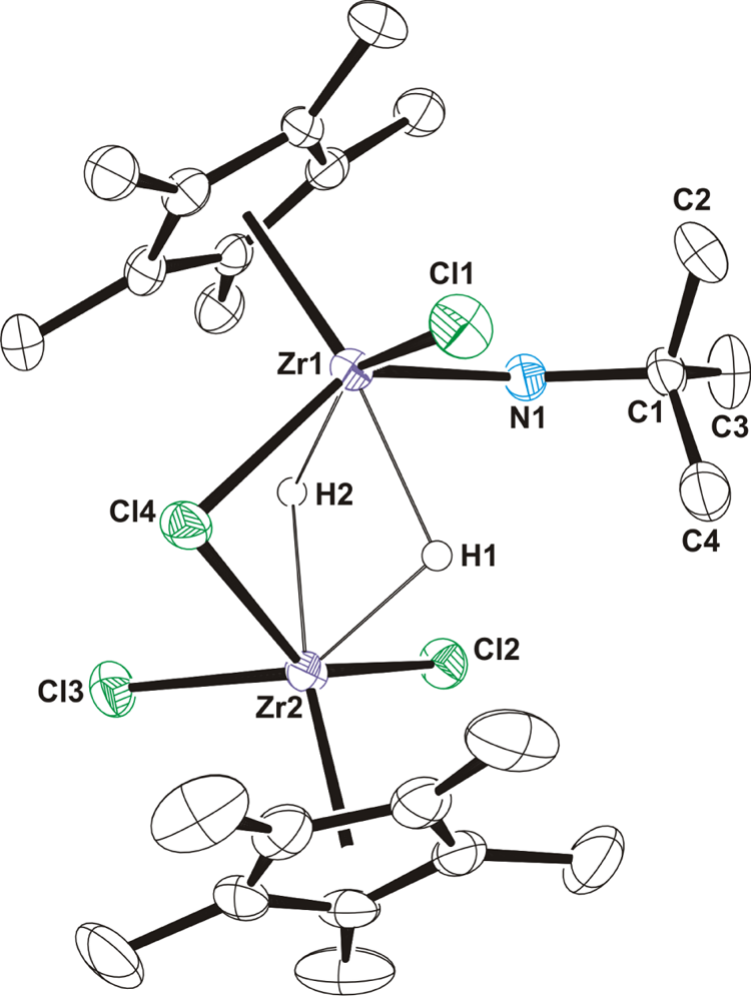
ORTEP diagram (50% probability level) of one of the enantiomers of **11**. Hydrogen atoms bound to carbon and nitrogen atoms are omitted for clarity. Selected lengths (Å) and angles (deg) for the two independent molecules: Zr–H 1.88(5)–2.14(6), Zr–Cl_bridging_ 2.564(2)–2.604(2), Zr–Cl_terminal_ 2.436(2)–2.472(2), Zr–N 2.339(5) and 2.342(5), Zr···Zr 3.217(1) and 3.219(1), H–Zr–H 65(2) and 67(2), H–Zr–Cl_bridging_ 67(2)–78(2), Zr–Cl_bridging_–Zr 77.0(1) and 77.2(1), and Zr–N–C 128.5(4) and 129.1(3).

Whereas complex **12** precipitated as a solvate with a half-molecule of toluene, **11** crystallized as a solvent-free compound containing two enantiomers in the asymmetric unit. The structural arrangement of both enantiomers in **11** is similar, so the structure of one of them is shown in Figure 4. The structures of the second enantiomer of **11** and the hafnium analogue **12** are presented in Figures S8 and S9, respectively. Molecules of **11** and **12** consist of one {M(η^5^-C_5_Me_5_)Cl_2_} and one {M(η^5^-C_5_Me_5_)Cl(NH_2_*t*Bu)} units held together by two hydrogen and one chlorine bridging atoms. Thus, each metal center adopts a distorted octahedral disposition with the η^5^-C_5_Me_5_ group and one hydride ligand occupying *trans* positions. In each dinuclear molecule, the two bridging hydrides show unsymmetrical coordination between the two metal centers, with M–H distances in the range of 1.88(5)–2.14(6) Å in **11** and 1.91(7)–2.03(7) Å in **12**. The M···M separations of 3.218(1) Å (average) in **11** and 3.172(1) Å in **12** are longer than those found in the dinuclear anionic fragment [{M(η^5^-C_5_Me_5_)Cl_2_}_2_(μ-H)_3_]^−^ of compounds **9** (3.106(3) Å)^33^ and **10** (3.048(1) Å). These M···M distances are comparable to those found in other zirconium and hafnium complexes with three bridging hydride ligands,^45^ but for instance the Zr···Zr distance in **11** is shorter than that of 3.460(1) Å in the doubly hydride-bridged dimer [{Zr(η^5^-C_5_H_4_Me)_2_H(μ-H)}_2_].^42a^

**Scheme 6:**
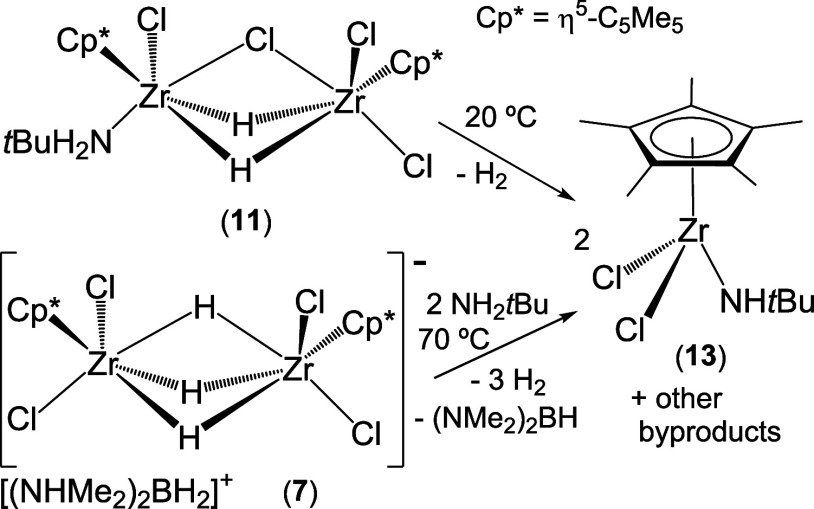
Synthesis of the Zirconium Complex **13**

Several attempts to isolate **11** in a pure form failed because the addition of lower molar ratios of NH_2_*t*BuBH_3_ on [Zr(η^5^-C_5_Me_5_)Cl_2_(NMe_2_)] always gave a mixture of complexes **9** and **11**, which exhibit similar solubility in common solvents. The higher stability of reactive intermediates for hafnium when compared with that for zirconium can be attributed to the higher hafnium-ligand bond dissociation energy.^49^ Indeed, unlike hafnium complex **12**, which is thermally stable in benzene-*d*_6_ solution at room temperature for several days, the zirconium analogue **11** decomposes within a few hours to give the mononuclear *tert*-butylamido complex [Zr(η^5^-C_5_Me_5_)Cl_2_(NH*t*Bu)] (**13**) along with molecular hydrogen and other unidentified species according to NMR spectroscopy (Scheme 6). The formation of compound **13** was also observed in the reaction of salts **7** and **9** with excess NH_2_*t*Bu in benzene-d_6_. Indeed, complex **13** could be obtained as colorless crystals in a 22% yield by treatment of **7** with excess NH_2_*t*Bu in toluene at 70 °C for 3 days (Scheme 6).

**Figure 5:**
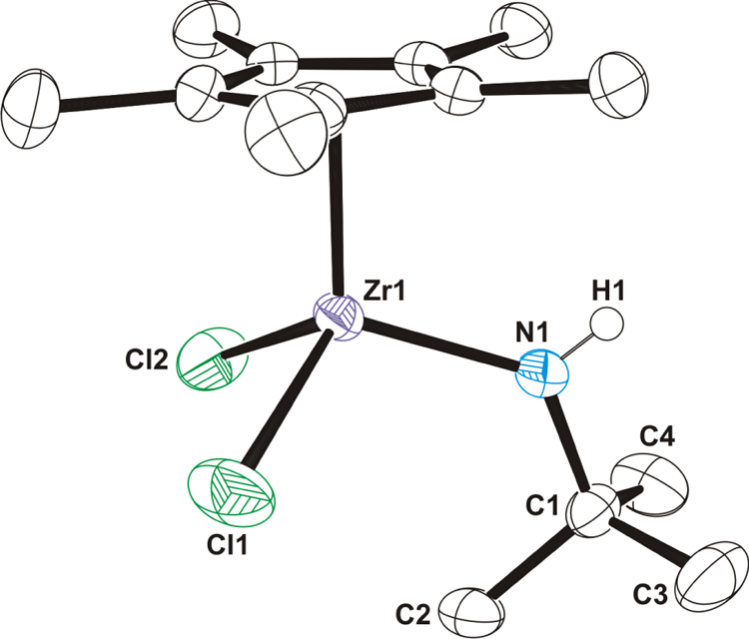
ORTEP diagram (50% probability level) of **13**. Hydrogen atoms of the methyl groups are omitted for clarity. Selected lengths (Å) and angles (deg): Zr(1)–N(1) 2.007(3), Zr(1)–Cl(1) 2.410(1), Zr(1)–Cl(2) 2.412(1), N(1)–Zr(1)–Cl(1) 108.3(1), N(1)–Zr(1)–Cl(2) 107.8(1), Cl(1)–Zr(1)–Cl(2) 105.1(1), and Zr(1)–N(1)–C(1) 128.5(2).

The crystal structure of **13** shows a three-legged piano-stool arrangement, which is typical for group 4 half-sandwich complexes (Figure 5). While the zirconium–nitrogen bond distance of 2.007(3) Å in **13** is similar to those reported for compound [Zr(η^5^-C_5_Me_5_)(NH*t*Bu)_3_] (2.002(10) and 2.015(11) Å),^50^ the Zr–N–C angle of 128.5(2)° is narrower than those determined in this tris(*tert*-butylamido) complex (158.3(8) and 162.3(10)°) due to the lower steric congestion about zirconium in **13**. The crystal structure of **13** is fully comparable to that determined previously for the analogous *tert*-butylamido titanium complex [Ti(η^5^-C_5_Me_5_)Cl_2_(NH*t*Bu)], which shows a Ti–N–C angle of 133.1(3)°.^51^ The NH*t*Bu ligand of **13** is characterized by a medium-intensity absorption at 3322 cm^–1^ for the ν_NH_ vibration in the IR spectrum (KBr) and a broad resonance at δ = 6.10 for the NH proton in the ^1^H NMR spectrum.

**Scheme 7:**
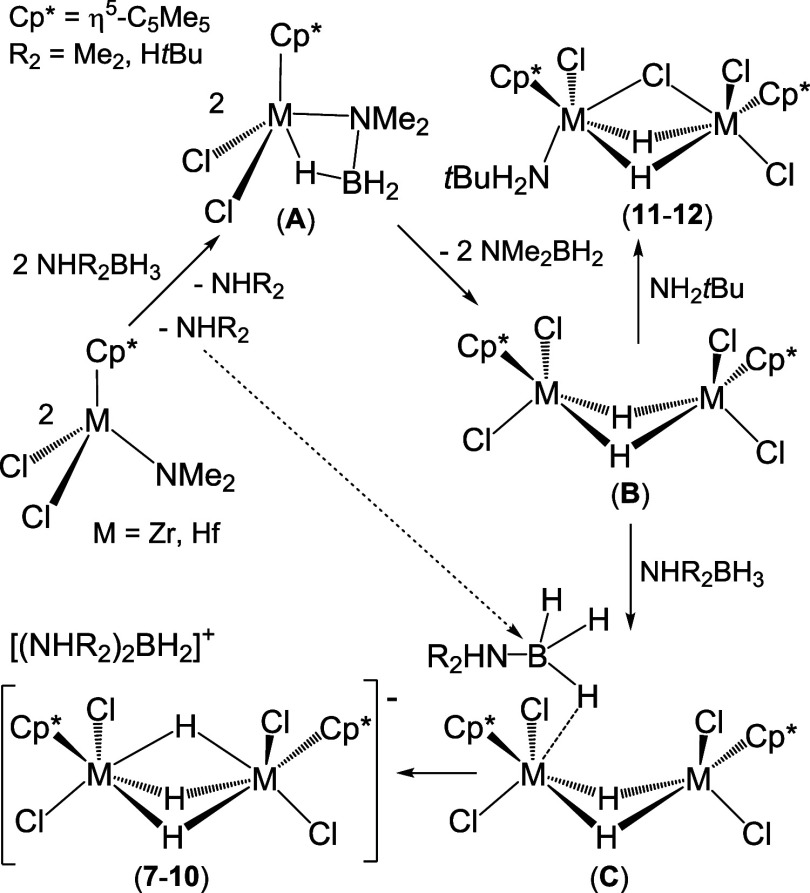
Plausible Proposal for the Formation of Ionic Compounds **7**–**10**

A plausible proposal for the formation of ionic compounds [(NHR_2_)_2_BH_2_][{M(η^5^-C_5_Me_5_)Cl_2_}_2_(μ-H)_3_] (**7**–**10**) is shown in Scheme 7. We speculate that the mono(dimethylamido) precursors [M(η^5^-C_5_Me_5_)Cl_2_(NMe_2_)] react with amine–boranes NHR_2_BH_3_ (R_2_ = Me_2_, H*t*Bu) to produce amines NHR_2_ and the amidoborane complexes [M(η^5^-C_5_Me_5_)Cl_2_(NMe_2_BH_3_)] (**A**). These intermediates readily undergo β-hydride elimination to give dimethylaminoborane and the hydride complexes [M(η^5^-C_5_Me_5_)Cl_2_H], which dimerize to form [{M(η^5^-C_5_Me_5_)Cl_2_}_2_(μ-H)_2_] (**B)**. The highly electrophilic group 4 species **B** would lead to hydride abstraction from an additional NHR_2_BH_3_ molecule in **C**, assisted by free amine NHR_2_, to produce the boronium cation [(NHR_2_)_2_BH_2_]^+^ and the dimetallic trihydride anion [{M(η^5^-C_5_Me_5_)Cl_2_}_2_(μ-H)_3_]^−^ of complexes **7**–**10**.^13,47b,52^ Alternatively, the reaction of intermediates **B** with NH_2_*t*Bu would result in the formation of the molecular complexes **11** and **12**.

## Conclusions

In contrast to previous studies on the reactions of amine–borane adducts NHR_2_BH_3_ with titanium(IV) complexes [Ti(η^5^-C_5_Me_5_)X_3_] that afforded paramagnetic titanium(III) species, the treatment with the zirconium and hafnium analogues leads to a variety of diamagnetic amidoborane and hydride derivatives with no change in the +4 oxidation state of the metal center. Whereas the tris(amidoborane) compounds [M(η^5^-C_5_Me_5_)(NH_2_BH_3_)_3_] are thermally stable in solution, the sterically encumbered analogous [M(η^5^-C_5_Me_5_)(NMe_2_BH_3_)_3_] immediately undergoes β-hydride elimination in one of the NMe_2_BH_3_ ligands to generate dimethylaminoborane and [M(η^5^-C_5_Me_5_)H(NMe_2_BH_3_)_2_]. The latter mononuclear hydride complexes display significant thermal stability in solution but readily react with *N*-*tert*-butylamine–borane to afford mixed bis(amidoborane) hydride derivatives [M(η^5^-C_5_Me_5_)H(NH*t*BuBH_3_)(NMe_2_BH_3_)]. In contrast to the aforementioned molecular species, ionic compounds [(NHR_2_)_2_BH_2_][{M(η^5^-C_5_Me_5_)Cl_2_}_2_(μ-H)_3_] containing boronium cations and dinuclear trihydride anions are formed by the addition of NHR_2_BH_3_ (R_2_ = Me_2_, H*t*Bu) on the mono(dimethylamido) complexes [M(η^5^-C_5_Me_5_)Cl_2_(NMe_2_)]. These reactions presumably involve mono(amidoborane) intermediates [M(η^5^-C_5_Me_5_)Cl_2_(NMe_2_BH_3_)], which rapidly undergo β-hydride elimination in the amidoborane ligand to generate hydride complexes [{M(η^5^-C_5_Me_5_)Cl_2_(μ-H)}_2_]. These dinuclear dihydride-bridged species have the potential to react with more NHR_2_BH_3_ via B–H bond activation, assisted by free amine NHR_2_, leading to trihydride [{M(η^5^-C_5_Me_5_)Cl_2_}_2_(μ-H)_3_]^−^ anions stabilized with the boronium cations [(NHR_2_)_2_BH_2_]^+^. Alternatively, the proposed dinuclear intermediates [{M(η^5^-C_5_Me_5_)Cl_2_(μ-H)}_2_] can be trapped with *tert*-butylamine to give more stable dinuclear complexes [Cl_2_(η^5^-C_5_Me_5_)M(μ-Cl)(μ-H)_2_M(η^5^-C_5_Me_5_)Cl(NH_2_*t*Bu)]. The insights gained from the well-defined species (i.e., amidoborane and hydride complexes, boronium cations stabilized with metal hydride anions) isolated in these stoichiometric reactions are expected to be useful in the understanding of metal-catalyzed dehydrogenation reactions of amine–boranes.

## Supporting Information Available

Experimental crystallographic data of complexes; powder X-ray diffractograms for compound **1**; perspective views and selected lengths and angles of the crystal structure of additional complexes; and selected IR and NMR spectra (PDF)
