# Supplementary material for: Half-Sandwich Zirconium and Hafnium Amidoborane Complexes: Precursors of Hydride Derivatives
Source: Inorg Chem. 2024 Apr 3;63(15):6576–88. doi: 10.1021/acs.inorgchem.3c02826 (PMC11022180; doi:10.1021/acs.inorgchem.3c02826)
Supplement: Supplementary file 1 — ic3c02826_si_001.pdf [file ic3c02826_si_001.pdf]

# Supporting Information for:

## Half-Sandwich Zirconium and Hafnium Amidoborane Complexes: Precursors of Hydride Derivatives

Maider Greño, Adrián Pérez-Redondo,* José Torrijos, Víctor Varela-Izquierdo, and Carlos Yélamos*

Departamento de Química Orgánica y Química Inorgánica, Instituto de Investigación Química "Andrés M. del Río" (IQAR), Universidad de Alcalá. 28805 Alcalá de Henares-Madrid (Spain). E-mail: carlos.yelamos@uah.es. E-mail: adrian.perez@uah.es.

## Contents:

- Experimental crystallographic data of complexes **1**, [Hf($\eta^5$-$C_5Me_5$)($CH_2CMe_2Ph$)$_3$], **4**, **5**, **6**, **10**, **11**, **12** and **13**.
- Experimental and simulated powder X-ray diffractograms for compound **1**.
- Perspective view of the crystal structure of complexes [Hf($\eta^5$-$C_5Me_5$)($CH_2CMe_2Ph$)$_3$], **4**, **5**, **10**, **11** and **12**.
- Tables for selected lengths and angles of the crystal structures of [Hf($\eta^5$-$C_5Me_5$)($CH_2CMe_2Ph$)$_3$], **10**, **11** and **12**.
- Selected IR spectra.
- Selected $^1$H, $^{13}$C{$^1$H} and $^{11}$B NMR spectra.

**Table S1**. Experimental Data for the X-ray Diffraction Studies on **1**, [Hf($\eta^5$-$C_5Me_5$)($CH_2CMe_2Ph$)$_3$], **4**, and **5**.

| | **1** | [Hf($\eta^5$-$C_5Me_5$)($CH_2CMe_2Ph$)$_3$] | **4** | **5** |
|---|---|---|---|---|
| Formula | $C_{10}H_{30}B_3N_3Zr$ | $C_{40}H_{54}Hf$ | $C_{14}H_{34}B_2HfN_2$ | $C_{16}H_{38}B_2N_2Zr$ |
| $M_r$ | 316.02 | 713.32 | 430.54 | 371.32 |
| $T$ [K] | 150(2) | 150(2) | 150(2) | 150(2) |
| $\lambda$ [Å] | 0.71073 | 0.71073 | 0.71073 | 0.71073 |
| crystal system | Monoclinic | Triclinic | Monoclinic | Monoclinic |
| space group | $P2_1/c$ | $P$–1 | $P2_1/n$ | $P2_1/c$ |
| $a$ [Å]; α [º] | 12.466(3) | 10.345(1); 106.78(1) | 8.598(2) | 14.720(2) |
| $b$ [Å]; β [º] | 9.227(2); 102.1(1) | 10.866(1); 92.67(1) | 26.128(4); 111.7(1) | 8.001(1); 95.83(1) |
| $c$ [Å]; γ [º] | 14.139(4) | 17.422(1); 113.38(1) | 8.657(2) | 17.104(3) |
| $V$ [Å$^3$] | 1590.2(7) | 1691.8(2) | 1806.7(7) | 2003.9(5) |
| Z | 4 | 2 | 4 | 4 |
| $\rho_{calcd}$ [g cm$^{-3}$] | 1.320 | 1.400 | 1.583 | 1.231 |
| $\mu_{MoK\alpha}$ [mm$^{-1}$] | 0.675 | 3.108 | 5.764 | 0.545 |
| *F(000)* | 664 | 732 | 856 | 792 |
| crystal size [mm$^3$] | 0.11 × 0.12 × 0.19 | 0.16 × 0.18 × 0.19 | 0.14 × 0.14 × 0.17 | 0.09 × 0.19 × 0.23 |
| $\theta$ range [deg] | 3.07 to 27.50 | 3.16 to 27.50 | 3.12 to 27.50 | 3.08 to 27.50 |
| index ranges | –16 to 16, –11 to 11, –18 to 18 | –13 to 13, –14 to 14, –22 to 22 | –11 to 11, –33 to 33, –11 to 11 | –19 to 19, –10 to 10, –22 to 22 |
| Reflections collected | 34719 | 38254 | 55214 | 40818 |
| Unique data | 3643 [$R_{int}$ = 0.089] | 7608 [$R_{int}$ = 0.055] | 4147 [$R_{int}$ = 0.073] | 4608 [$R_{int}$ = 0.092] |
| obsd data [I>2σ(I)] | 2975 | 6978 | 3130 | 3589 |
| Goodness-of-fit on F$^2$ | 1.408 | 1.104 | 1.158 | 1.227 |
| final R$^a$ indices [I>2σ(I)] | R1 = 0.061, wR2 = 0.131 | R1 = 0.027, wR2 = 0.062 | R1 = 0.035, wR2 = 0.063 | R1 = 0.060, wR2 = 0.139 |
| $R^a$ indices (all data) | R1 = 0.081, wR2 = 0.138 | R1 = 0.033, wR2 = 0.065 | R1 = 0.054, wR2 = 0.070 | R1 = 0.086, wR2 = 0.150 |
| largest diff. peak/hole [e Å$^{-3}$] | 0.986/–0.579 | 3.282/–1.949 | 3.505/–1.449 | 1.587/–0.867 |

$^a$ $R1 = \Sigma||F_0|-|F_c|| / [\Sigma|F_0|]$, $wR2 = \{[\Sigma w(F_0^2-F_c^2)^2] / [\Sigma w(F_0^2)^2]\}^{1/2}$

**Table S2**. Experimental Data for the X-ray Diffraction Studies on **6**, **10**, **11**, **12**, and **13**.

| | **6** | **10**·$CH_2Cl_2$ | **11** | **12**·0.5$C_7H_8$ | **13** |
|---|---|---|---|---|---|
| Formula | $C_{16}H_{38}B_2HfN_2$ | $C_{29}H_{59}BCl_6Hf_2N_2$ | $C_{24}H_{43}Cl_4NZr_2$ | $C_{27.5}H_{47}Cl_4Hf_2N$ | $C_{14}H_{25}Cl_2NZr$ |
| $M_r$ | 458.59 | 1016.27 | 669.83 | 890.44 | 369.47 |
| $T$ [K] | 150(2) | 150(2) | 150(2) | 150(2) | 150(2) |
| $\lambda$ [Å] | 0.71073 | 0.71073 | 0.71073 | 0.71073 | 0.71073 |
| crystal system | Monoclinic | Orthorhombic | Triclinic | Triclinic | Monoclinic |
| space group | $P2_1/c$ | *Fddd* | $P$–1 | $P$–1 | $P2_1/c$ |
| $a$ [Å]; α [º] | 14.703(1) | 16.097(2) | 11.386(1); 100.44(1) | 11.335(1); 112.56(1) | 8.852(1) |
| $b$ [Å]; β [º] | 8.007(1); 95.85(1) | 20.659(3) | 14.031(2); 94.79(1) | 11.361(1); 102.58(1) | 15.944(1); 103.79(1) |
| $c$ [Å]; γ [º] | 17.059(1) | 48.340(7) | 19.175(1); 93.02(1) | 14.158(1); 97.76(1) | 12.751(1) |
| $V$ [Å$^3$] | 1997.9(2) | 16075(4) | 2995.0(5) | 1594.2(2) | 1747.7(3) |
| Z | 4 | 16 | 4 | 2 | 4 |
| $\rho_{calcd}$ [g cm$^{-3}$] | 1.525 | 1.680 | 1.486 | 1.855 | 1.404 |
| $\mu_{MoK\alpha}$ [mm$^{-1}$] | 5.218 | 5.582 | 1.065 | 6.860 | 0.921 |
| *F(000)* | 920 | 7968 | 1368 | 862 | 760 |
| crystal size [mm$^3$] | 0.12 × 0.18 × 0.18 | 0.07 × 0.08 × 0.24 | 0.18 × 0.29 × 0.31 | 0.11 × 0.16 × 0.20 | 0.13 × 0.18 × 0.28 |
| $\theta$ range [deg] | 3.08 to 27.49 | 2.67 to 26.43 | 3.05 to 27.50 | 1.90 to 27.56 | 3.04 to 27.50 |
| index ranges | –19 to 19, –10 to 10, –22 to 22 | –20 to 20, –20 to 25, –60 to 60 | –14 to 14, –18 to 18, –24 to 24 | –14 to 14, –14 to 14, –18 to 18 | –11 to 11, –20 to 20, –16 to 16 |
| Reflections collected | 42491 | 35967 | 92624 | 86794 | 36168 |
| Unique data | 4592 [$R_{int}$ = 0.129] | 4127 [$R_{int}$ = 0.156] | 13569 [$R_{int}$ = 0.106] | 7334 [$R_{int}$ = 0.037] | 4021 [$R_{int}$ = 0.065] |
| obsd data [I>2σ(I)] | 3467 | 2719 | 8887 | 6656 | 3086 |
| Goodness-of-fit on $F^2$ | 1.180 | 1.093 | 1.101 | 1.248 | 1.162 |
| final R[a] indices [I>2σ(I)] | R1 = 0.055, wR2 = 0.113 | R1 = 0.079, wR2 = 0.177 | R1 = 0.056, wR2 = 0.116 | R1 = 0.021, wR2 = 0.075 | R1 = 0.042, wR2 = 0.097 |
| *R*[a] indices (all data) | R1 = 0.087, wR2 = 0.128 | R1 = 0.124, wR2 = 0.200 | R1 = 0.114, wR2 = 0.149 | R1 = 0.026, wR2 = 0.090 | R1 = 0.066, wR2 = 0.113 |
| largest diff. peak/hole [e Å$^{-3}$] | 5.378/–2.754 | 5.528/–1.743 | 1.782/–1.054 | 0.841/–1.663 | 1.516/–0.739 |

[a] $R1 = \Sigma||F_0|-|F_c|| / [\Sigma|F_0|]$, $wR2 = \{[\Sigma w(F_0^2-F_c^2)^2] / [\Sigma w(F_0^2)^2]\}^{1/2}$

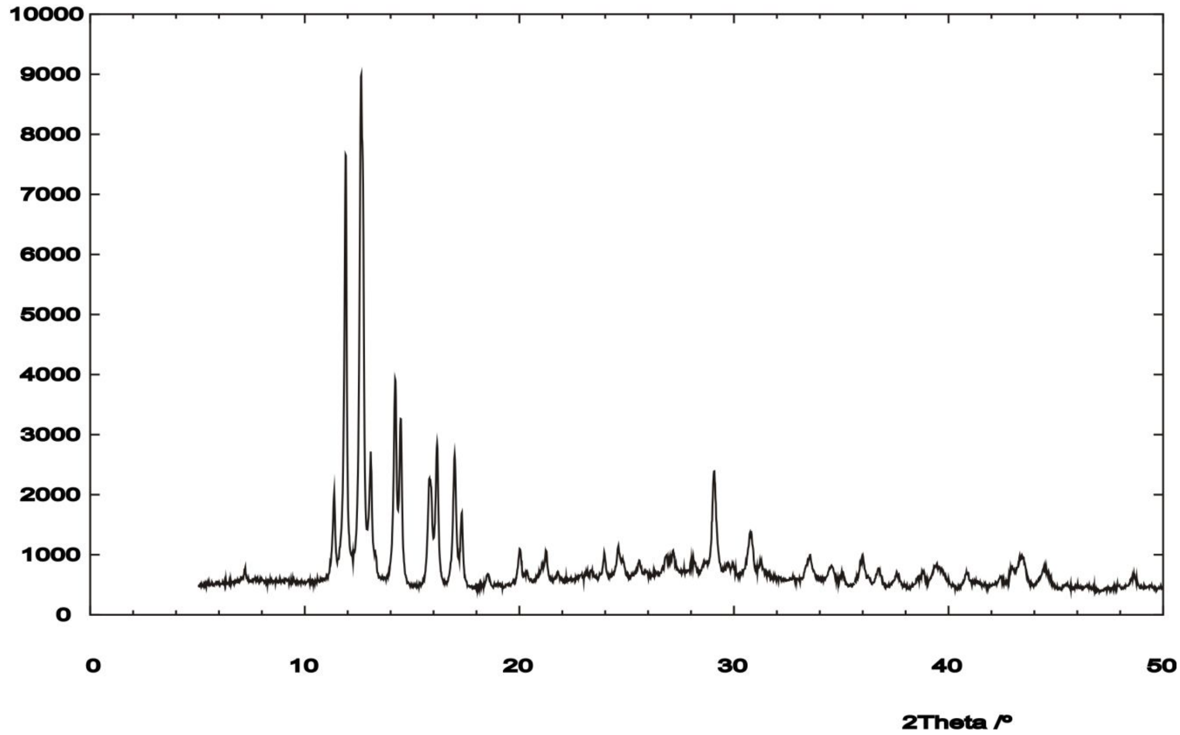


**Figure S1.** Experimental powder X-ray diffractogram for compound **1**.

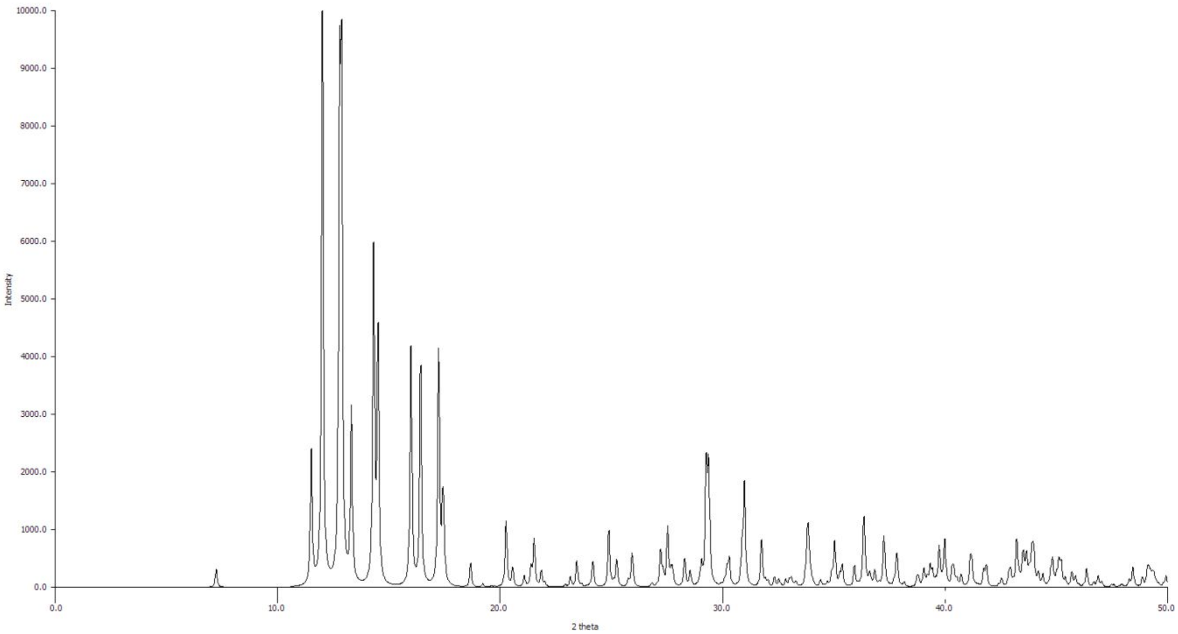


**Figure S2.** Simulated powder X-ray diffractogram obtained from the single crystal X-ray determination for compound **1**.

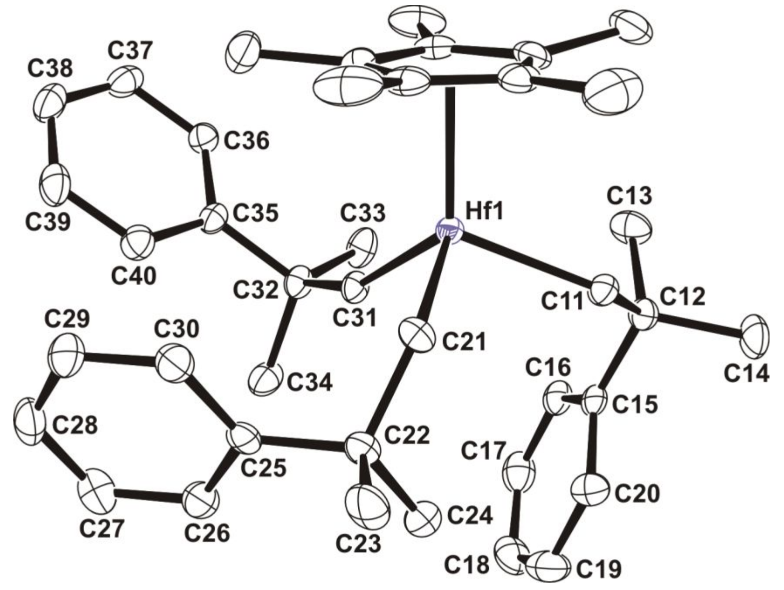


**Figure S3.** Perspective view of [Hf($\eta^5$-$C_5Me_5$)($CH_2CMe_2Ph$)$_3$] with thermal ellipsoids at the 50% probability level. Hydrogen atoms are omitted for clarity.

**Table S3.** Selected Lengths (Å) and Angles (deg) for [Hf($\eta^5$-$C_5Me_5$)($CH_2CMe_2Ph$)$_3$].

| | | | |
|---|---|---|---|
| Hf(1)–C(11) | 2.248(3) | Hf(1)–C(21) | 2.267(3) |
| Hf(1)–C(31) | 2.148(3) | | |
| C(11)–Hf(1)–C(21) | 98.6(1) | C(11)–Hf(1)–C(31) | 104.9(1) |
| C(21)–Hf(1)–C(31) | 104.7(1) | Hf(1)–C(11)–C(12) | 131.8(2) |
| Hf(1)–C(21)–C(22) | 128.7(2) | Hf(1)–C(31)–C(32) | 156.2(2) |
| C(11)–C(12)–C(15) | 111.7(3) | C(21)–C(22)–C(25) | 110.8(3) |
| C(31)–C(32)–C(35) | 109.3(3) | | |

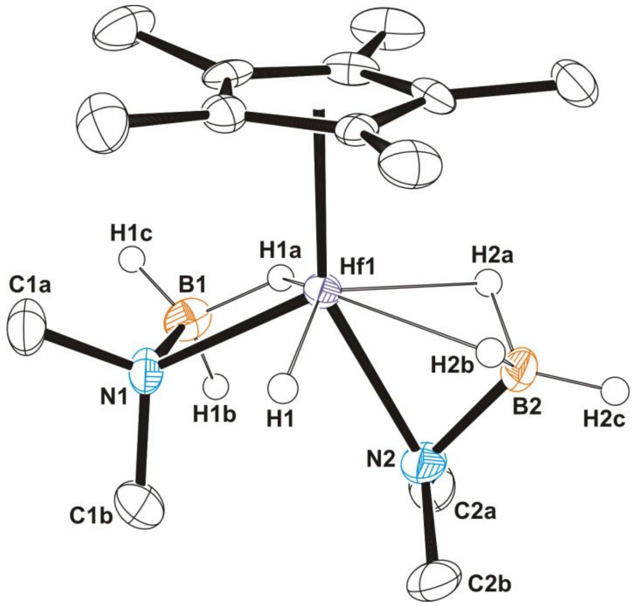


**Figure S4.** Perspective view of complex **4** (thermal ellipsoids at the 50% probability level). Hydrogen atoms of the methyl groups are omitted for clarity.

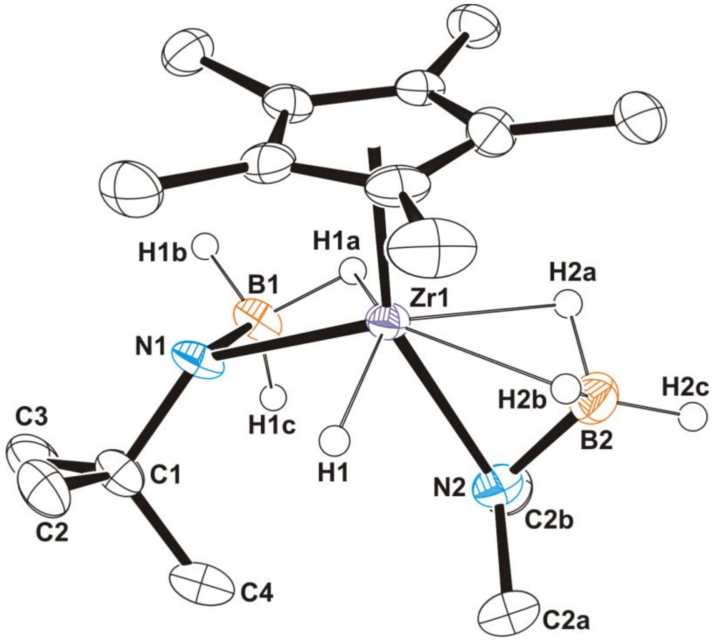


**Figure S5.** Perspective view of **5** with thermal ellipsoids at the 50% probability level. Hydrogen atoms bound to nitrogen and carbon atoms are omitted for clarity.

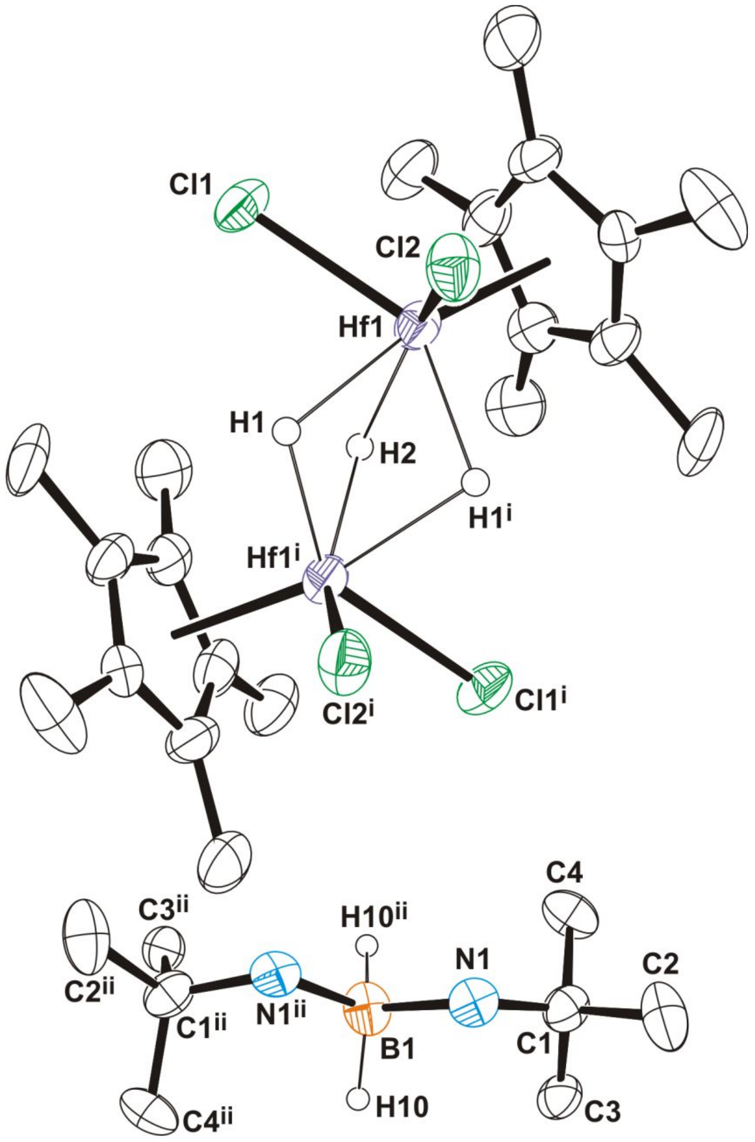


**Figure S6.** Perspective view with thermal ellipsoids at the 50% probability level of **10**·$CH_2Cl_2$. Dichloromethane solvent molecule and hydrogen atoms bound to carbon or nitrogen atoms are omitted for clarity. Symmetry code: (i) –x + 7/4, –y + 3/4, z; (ii) –x + 5/4, y, –z + 5/4.

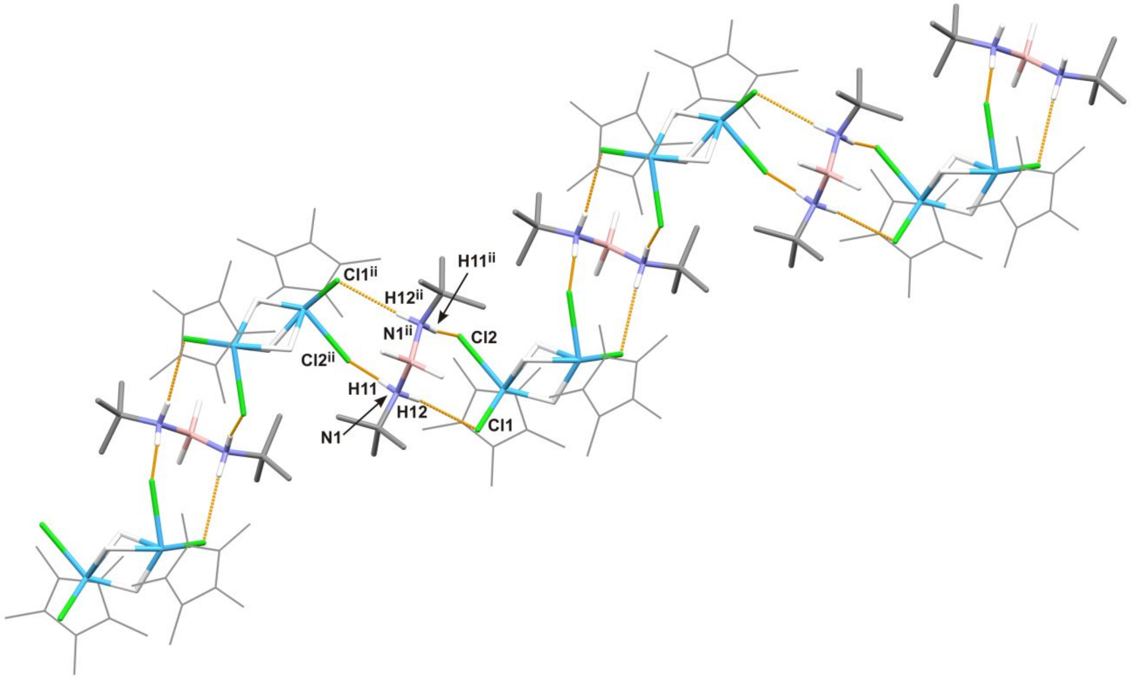


**Figure S7.** Anion and cation fragments organized in a helical chain by hydrogen bonding interactions in the solid-state structure of compound **10**. Dichloromethane solvent molecules and hydrogen atoms of the methyl groups are not shown for clarity. Symmetry code: (ii) –x + 5/4, y, –z + 5/4.

**Table S4.** Relevant hydrogen bonds for compound **10**.[a]

| D–H···A | D···A/Å | H···A/Å | D–H···A/º |
|---|---|---|---|
| N(1)–H(12)···Cl(1) | 3.334(12) | 2.46(1) | 162(1) |
| N(1)–H(11)···Cl(2)ii | 3.266(12) | 2.39(1) | 163(1) |

[a]Symmetry transformation: (ii) –x + 5/4, y, –z + 5/4. A = acceptor; D = donor.

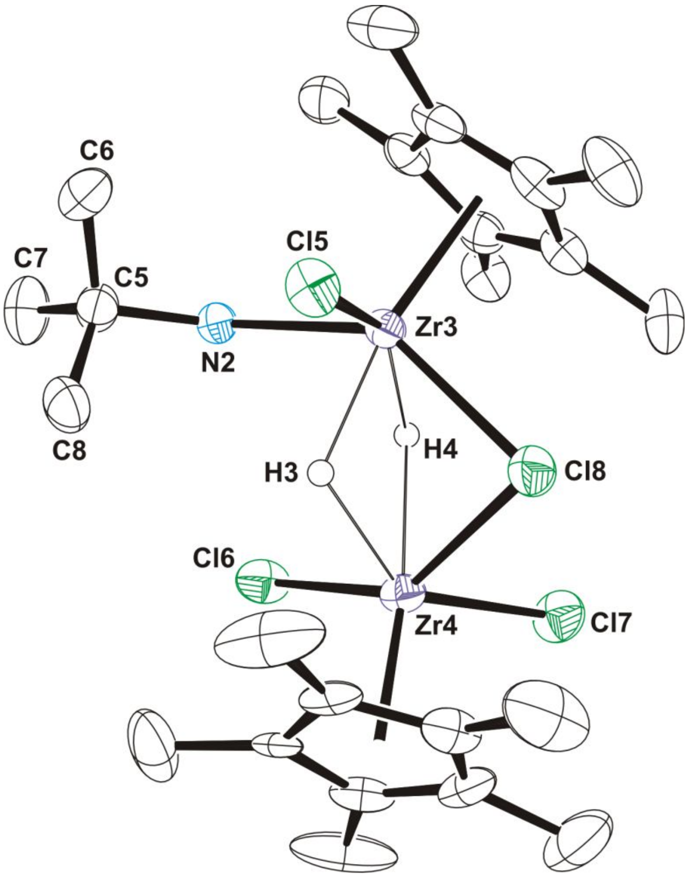


**Figure S8.** Perspective view with thermal ellipsoids at the 50% probability level of the second enantiomer of **11**. Hydrogen atoms bound to carbon and nitrogen atoms are omitted for clarity.

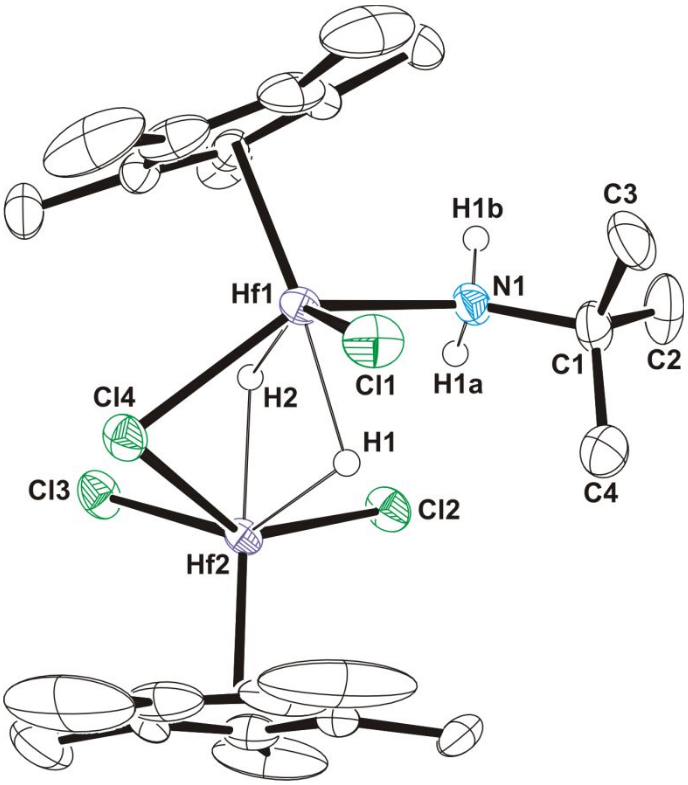


**Figure S9.** Perspective view with thermal ellipsoids at the 50% probability level of **12**. Toluene solvent molecule and hydrogen atoms of the methyl groups are omitted for clarity.

**Table S5.** Selected Lengths (Å) and Angles (deg) for **11**.

| Enantiomer 1 | | Enantiomer 2 | |
|---|---|---|---|
| Zr(1)–H(1) | 2.11(6) | Zr(3)–H(3) | 1.88(5) |
| Zr(1)–H(2) | 1.98(6) | Zr(3)–H(4) | 2.02(6) |
| Zr(1)–N(1) | 2.342(5) | Zr(3)–N(2) | 2.339(5) |
| Zr(1)–Cl(1) | 2.442(2) | Zr(3)–Cl(5) | 2.442(2) |
| Zr(1)–Cl(4) | 2.570(1) | Zr(3)–Cl(8) | 2.564(2) |
| Zr(2)–H(1) | 1.93(7) | Zr(4)–H(3) | 1.93(5) |
| Zr(2)–H(2) | 2.05(6) | Zr(4)–H(4) | 2.14(6) |
| Zr(2)–Cl(2) | 2.472(2) | Zr(4)–Cl(6) | 2.466(2) |
| Zr(2)–Cl(3) | 2.439(2) | Zr(4)–Cl(7) | 2.436(2) |
| Zr(2)–Cl(4) | 2.588(2) | Zr(4)–Cl(8) | 2.604(2) |
| Zr(1)···Zr(2) | 3.219(1) | Zr(3)···Zr(4) | 3.217(1) |
| H(1)–Zr(1)–H(2) | 65(2) | H(3)–Zr(3)–H(4) | 65(2) |
| H(1)–Zr(1)–N(1) | 71(2) | H(3)–Zr(3)–N(2) | 75(2) |
| H(1)–Zr(1)–Cl(1) | 85(2) | H(3)–Zr(3)–Cl(5) | 84(2) |
| H(1)–Zr(1)–Cl(4) | 72(2) | H(3)–Zr(3)–Cl(8) | 68(2) |
| H(2)–Zr(1)–N(1) | 79(2) | H(4)–Zr(3)–N(2) | 77(2) |
| H(2)–Zr(1)–Cl(1) | 149(2) | H(4)–Zr(3)–Cl(5) | 149(2) |
| H(2)–Zr(1)–Cl(4) | 73(2) | H(4)–Zr(3)–Cl(8) | 78(2) |
| Cl(1)–Zr(1)–Cl(4) | 88.8(1) | Cl(5)–Zr(3)–Cl(8) | 88.2(1) |
| Cl(1)–Zr(1)–N(1) | 101.4(1) | Cl(5)–Zr(3)–N(2) | 99.0(1) |
| Cl(4)–Zr(1)–N(1) | 140.7(1) | Cl(8)–Zr(3)–N(2) | 142.0(1) |
| H(1)–Zr(2)–H(2) | 67(2) | H(3)–Zr(4)–H(4) | 62(2) |
| H(1)–Zr(2)–Cl(2) | 81(2) | H(3)–Zr(4)–Cl(6) | 86(2) |
| H(1)–Zr(2)–Cl(3) | 146(2) | H(3)–Zr(4)–Cl(7) | 139(2) |
| H(1)–Zr(2)–Cl(4) | 74(2) | H(3)–Zr(4)–Cl(8) | 67(2) |
| H(2)–Zr(2)–Cl(2) | 74(2) | H(4)–Zr(4)–Cl(6) | 71(2) |
| H(2)–Zr(2)–Cl(3) | 80(2) | H(4)–Zr(4)–Cl(7) | 81(2) |
| H(2)–Zr(2)–Cl(4) | 72(2) | H(4)–Zr(4)–Cl(8) | 76(2) |
| Cl(2)–Zr(2)–Cl(3) | 96.6(1) | Cl(6)–Zr(4)–Cl(7) | 99.1(1) |
| Cl(2)–Zr(2)–Cl(4) | 143.4(1) | Cl(6)–Zr(4)–Cl(8) | 143.7(1) |
| Cl(3)–Zr(2)–Cl(4) | 89.4(1) | Cl(7)–Zr(4)–Cl(8) | 88.6(1) |
| Zr(1)–Cl(4)–Zr(2) | 77.2(1) | Zr(3)–Cl(8)–Zr(4) | 77.0(1) |
| Zr(1)–N(1)–C(1) | 129.1(3) | Zr(3)–N(2)–C(5) | 128.5(4) |

**Table S6.** Selected Lengths (Å) and Angles (deg) for **12**.

| | | | |
|---|---|---|---|
| Hf(1)–H(1) | 1.91(7) | Hf(1)–H(2) | 2.03(7) |
| Hf(1)–N(1) | 2.314(4) | Hf(1)–Cl(1) | 2.415(1) |
| Hf(1)–Cl(4) | 2.538(1) | Hf(2)–H(1) | 1.95(7) |
| Hf(2)–H(2) | 1.94(6) | Hf(2)–Cl(2) | 2.453(1) |
| Hf(2)–Cl(3) | 2.409(1) | Hf(2)–Cl(4) | 2.585(1) |
| Hf(1)···Hf(2) | 3.172(1) | | |
| H(1)–Hf(1)–H(2) | 66(3) | H(1)–Hf(1)–N(1) | 73(2) |
| H(1)–Hf(1)–Cl(1) | 81(2) | H(1)–Hf(1)–Cl(4) | 71(2) |
| H(2)–Hf(1)–N(1) | 82(2) | H(2)–Hf(1)–Cl(1) | 146(2) |
| H(2)–Hf(1)–Cl(4) | 70(2) | Cl(1)–Hf(1)–Cl(4) | 90.0(1) |
| Cl(1)–Hf(1)–N(1) | 99.0(1) | Cl(4)–Hf(1)–N(1) | 141.3(1) |
| H(1)–Hf(2)–H(2) | 67(3) | H(1)–Hf(2)–Cl(2) | 85(2) |
| H(1)–Hf(2)–Cl(3) | 141(2) | H(1)–Hf(2)–Cl(4) | 69(2) |
| H(2)–Hf(2)–Cl(2) | 76(2) | H(2)–Hf(2)–Cl(3) | 76(2) |
| H(2)–Hf(2)–Cl(4) | 70(2) | Cl(2)–Hf(2)–Cl(3) | 96.2(1) |
| Cl(2)–Hf(2)–Cl(4) | 143.9(1) | Cl(3)–Hf(2)–Cl(4) | 88.7(1) |
| Hf(1)–Cl(4)–Hf(2) | 76.5(1) | Hf(1)–N(1)–C(1) | 130.4(3) |

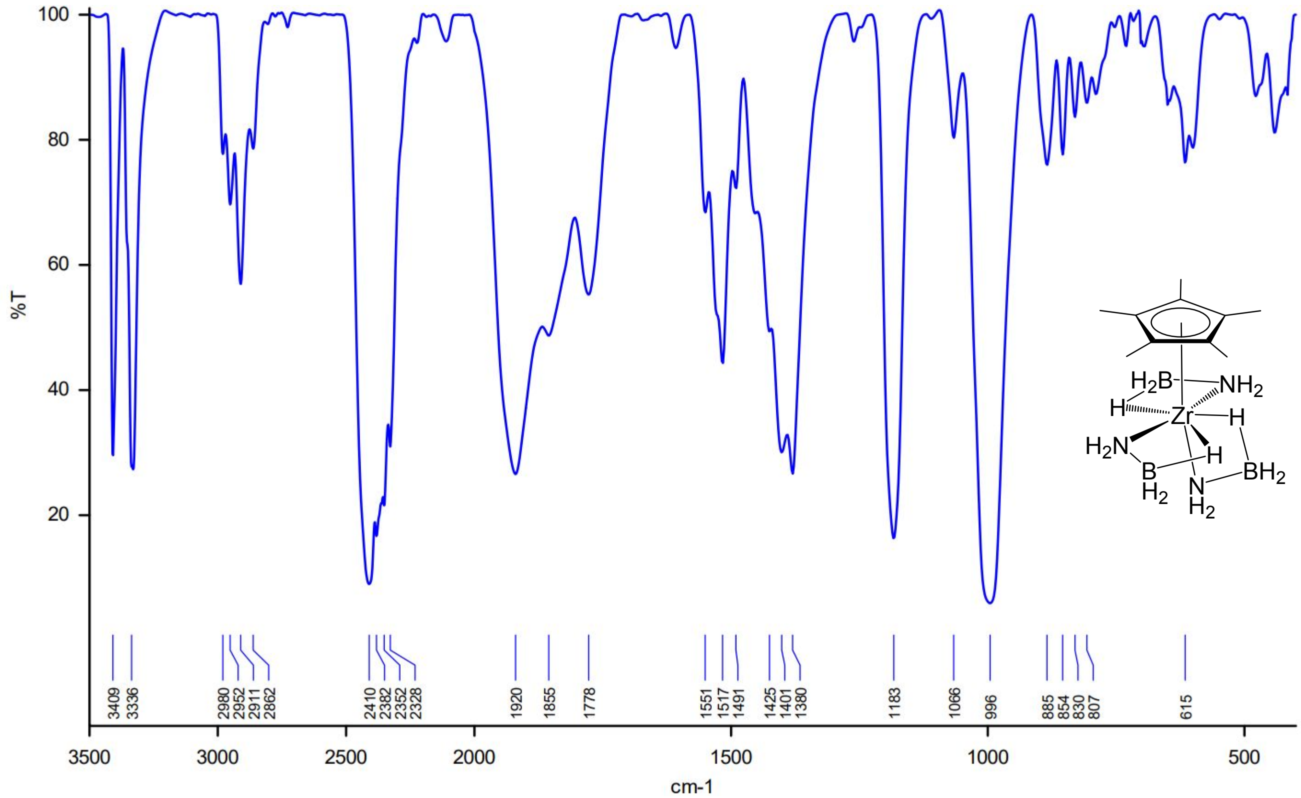


**Figure S10.** IR spectrum (KBr) of [Zr($\eta^5$-$C_5Me_5$)($NH_2BH_3$)$_3$] (**1**).

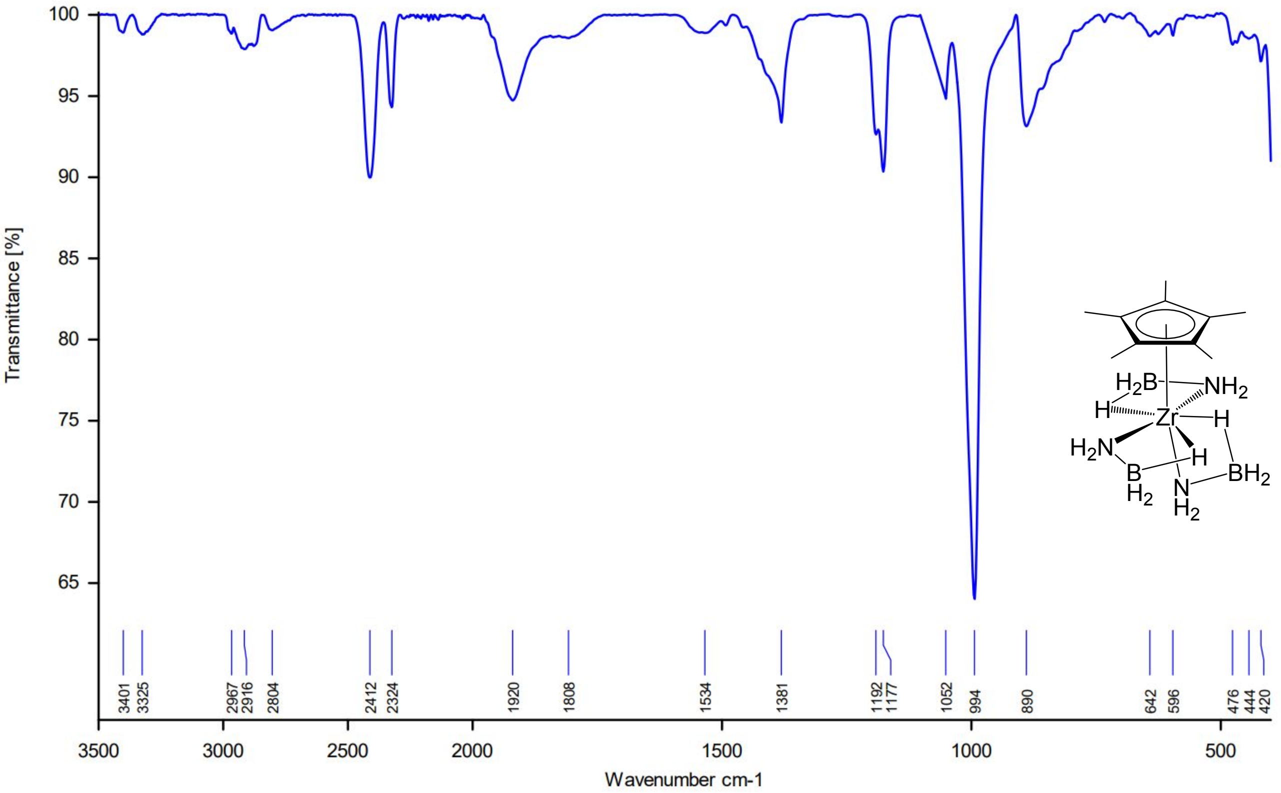


**Figure S11.** IR spectrum (thf) of [Zr($\eta^5$-$C_5Me_5$)($NH_2BH_3$)$_3$] (**1**).

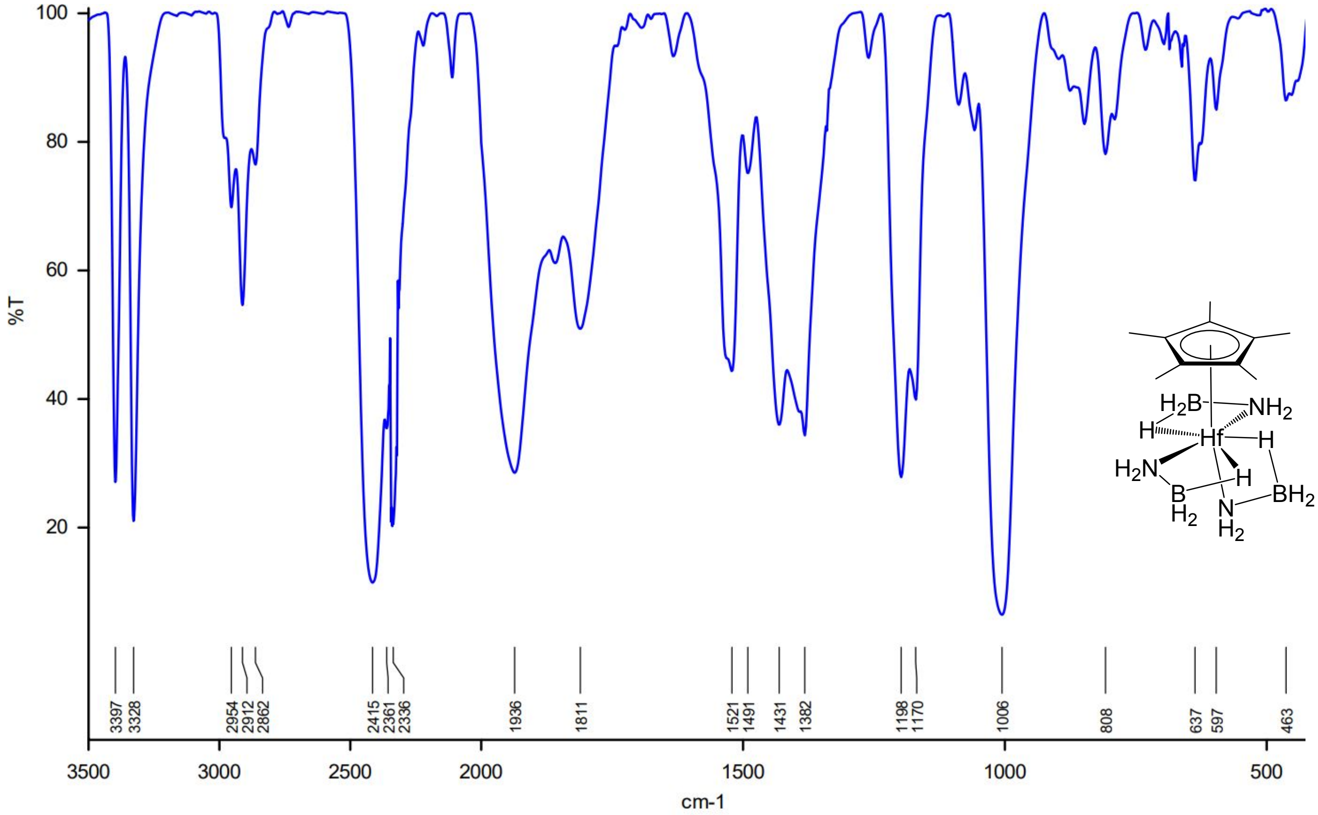


**Figure S12.** IR spectrum (KBr) of [Hf($\eta^5$-$C_5Me_5$)($NH_2BH_3$)$_3$] (**2**).

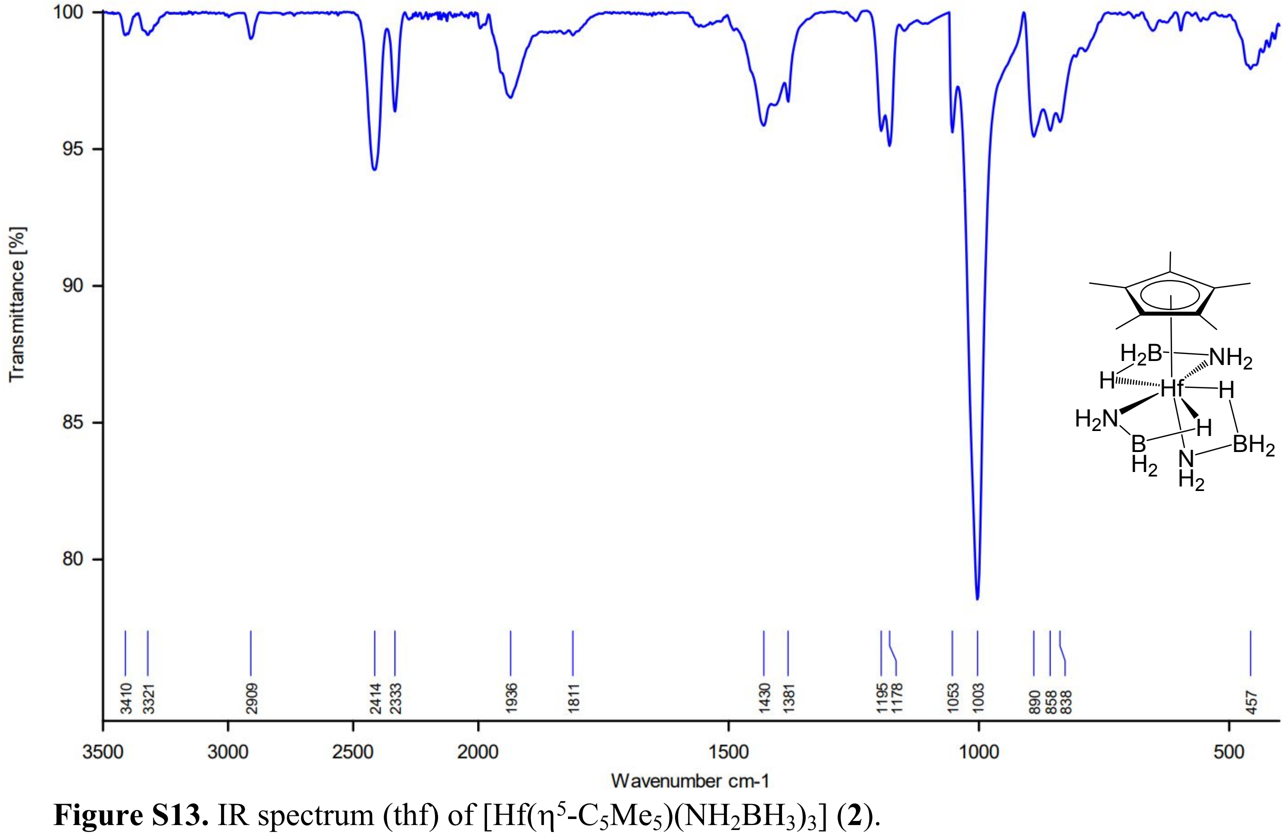


**Figure S13.** IR spectrum (thf) of [Hf($\eta^5$-$C_5Me_5$)($NH_2BH_3$)$_3$] (**2**).

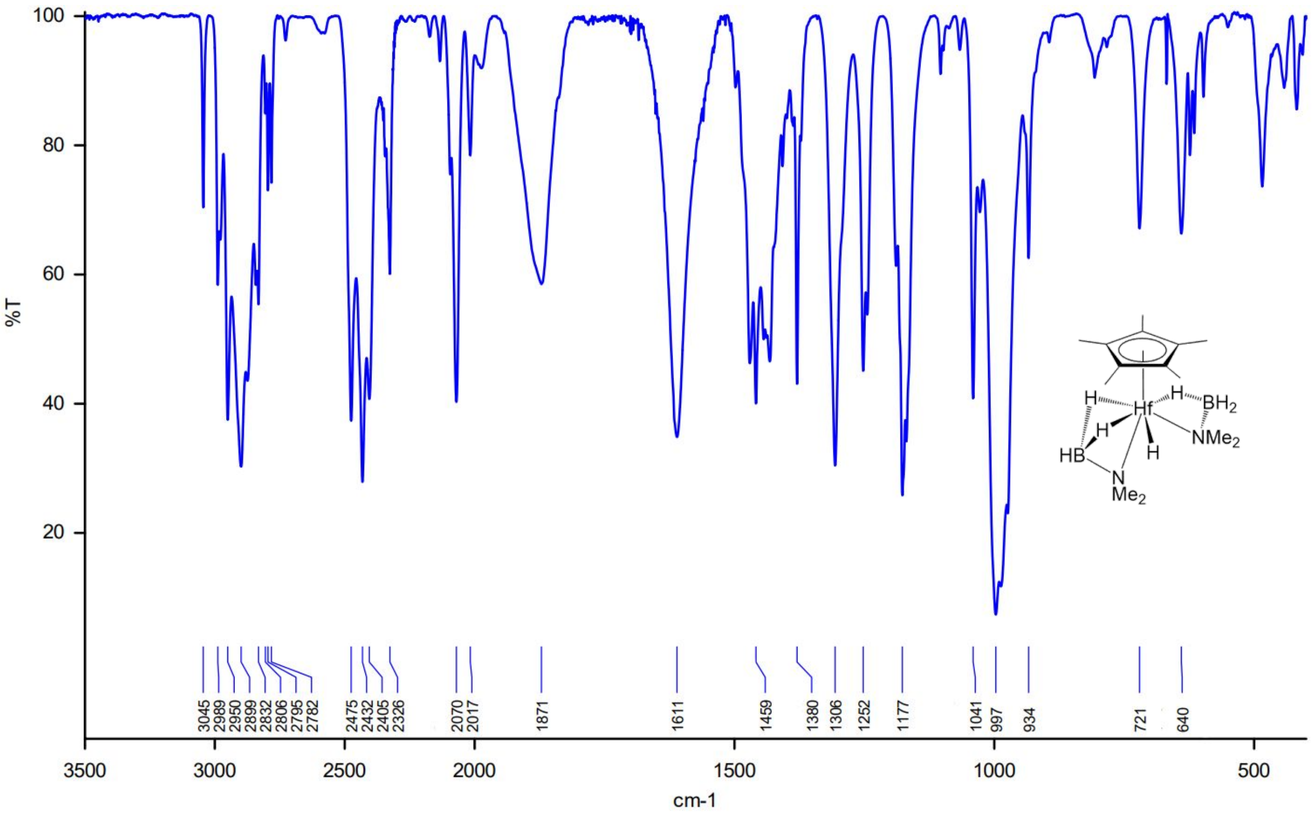


**Figure S14.** IR spectrum (KBr) of [Hf($\eta^5$-$C_5Me_5$)H($NMe_2BH_3$)$_2$] (**4**).

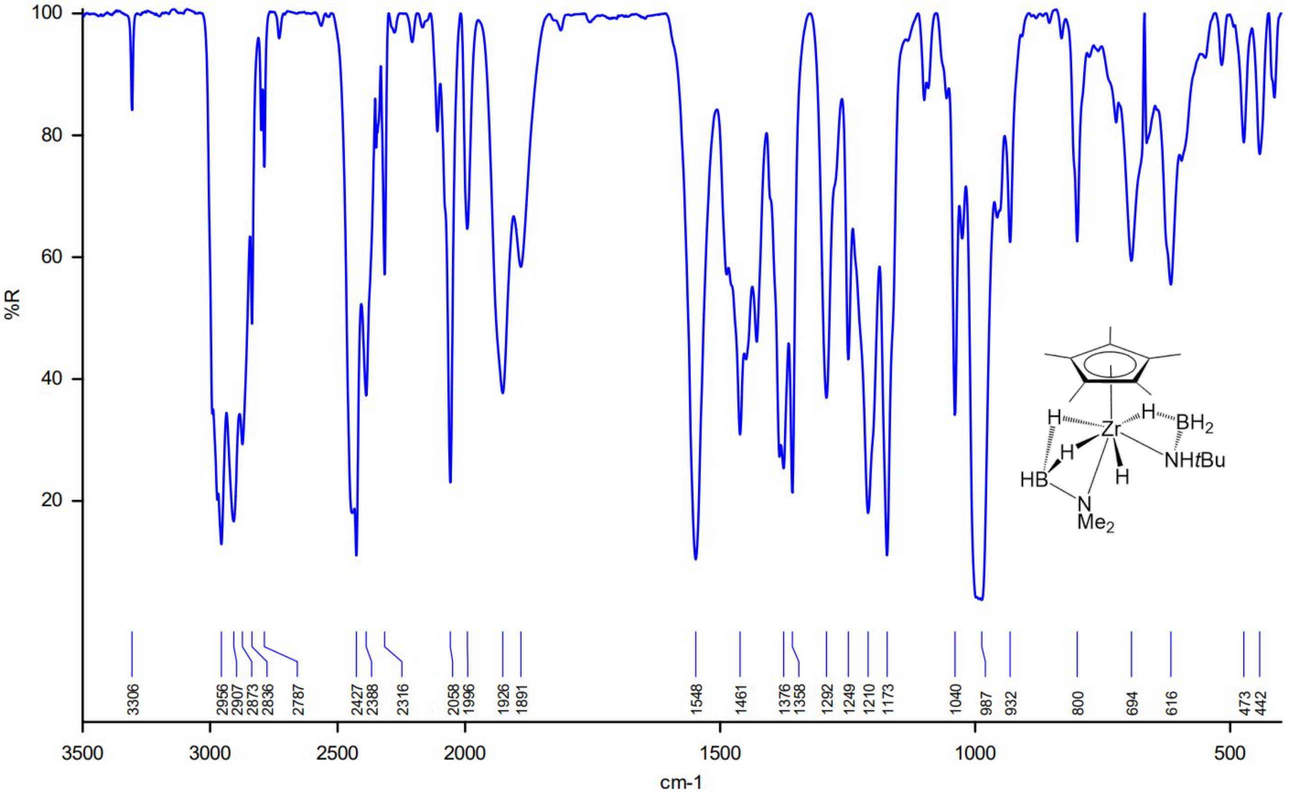


**Figure S15.** IR spectrum (KBr) of [Zr($\eta^5$-$C_5Me_5$)H($NH$*t*$BuBH_3$)($NMe_2BH_3$)] (**5**).

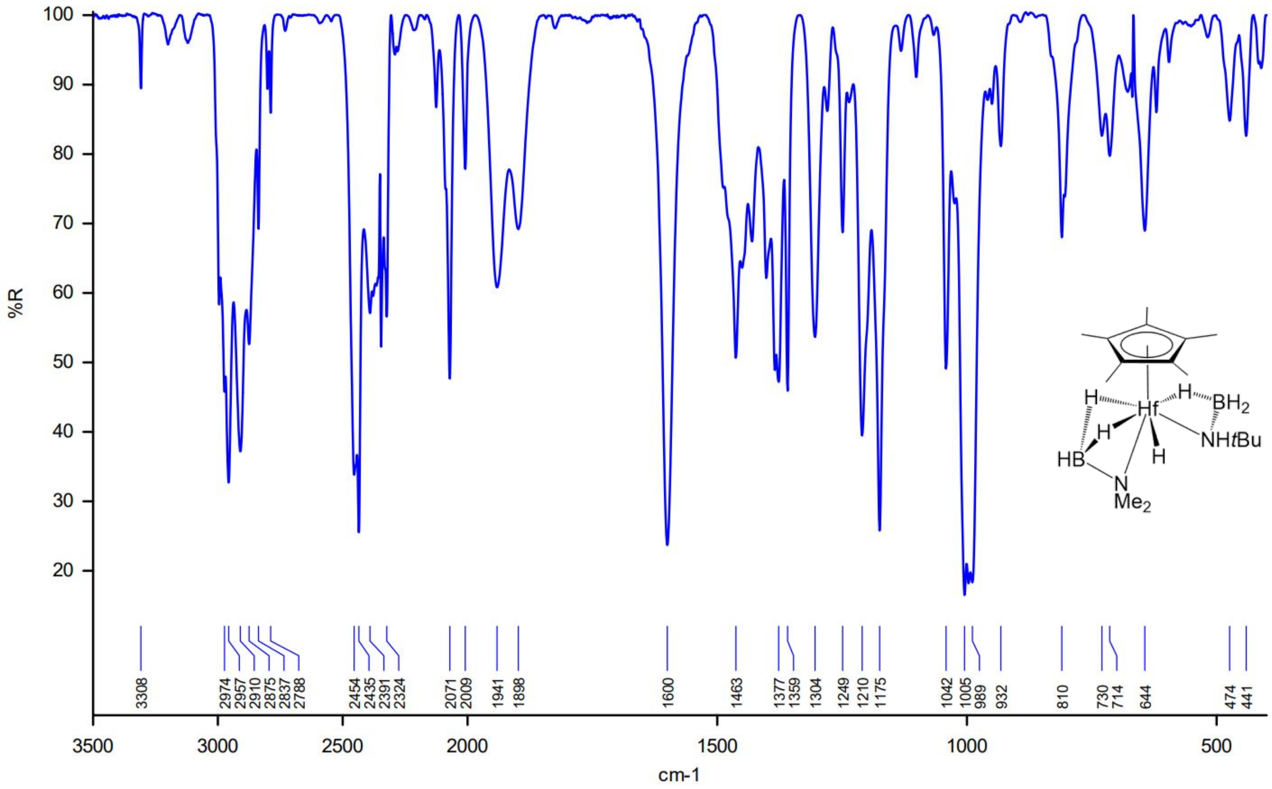


**Figure S16.** IR spectrum (KBr) of [Hf($\eta^5$-$C_5Me_5$)H(NH*t*Bu$BH_3$)($NMe_2BH_3$)] (**6**).

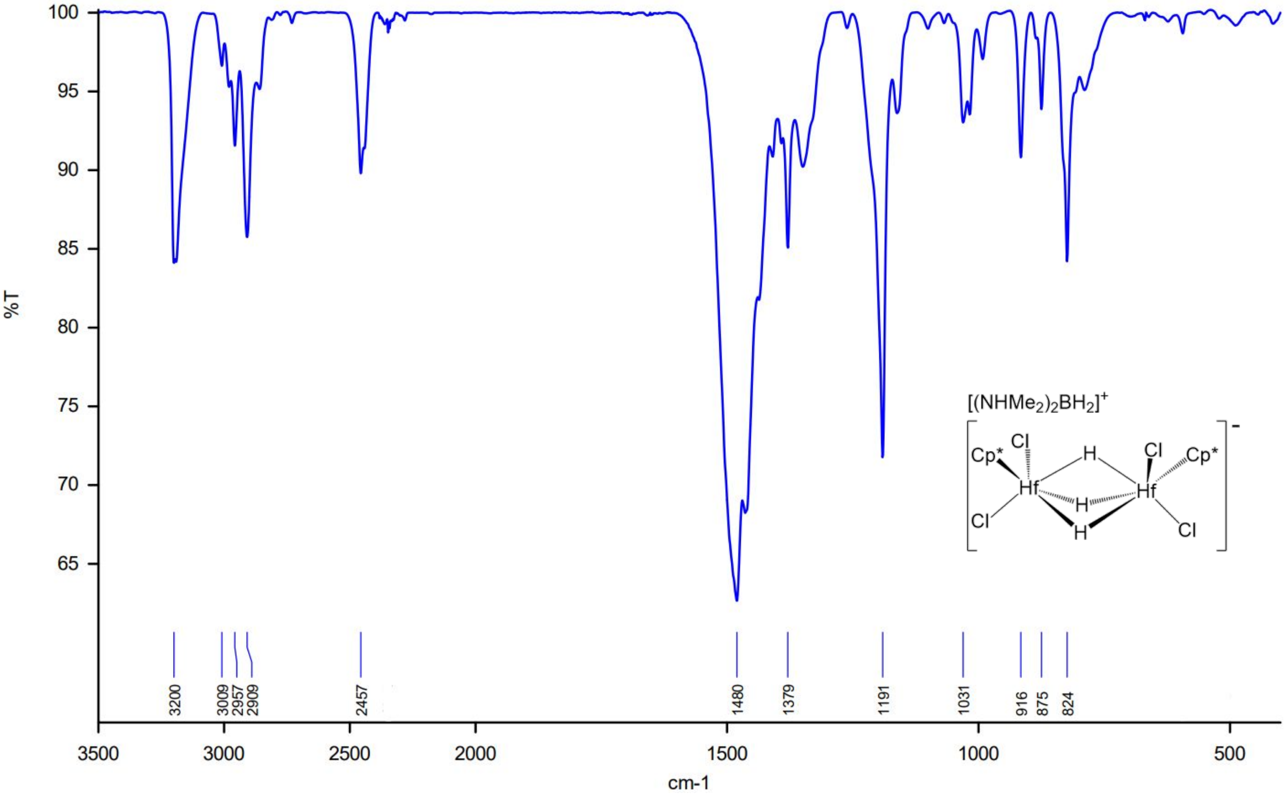


**Figure S17.** IR spectrum (KBr) of [$(NHMe_2)_2BH_2$][{Hf($\eta^5$-$C_5Me_5$)$Cl_2$}$_2$($\mu$-H)$_3$] (**8**).

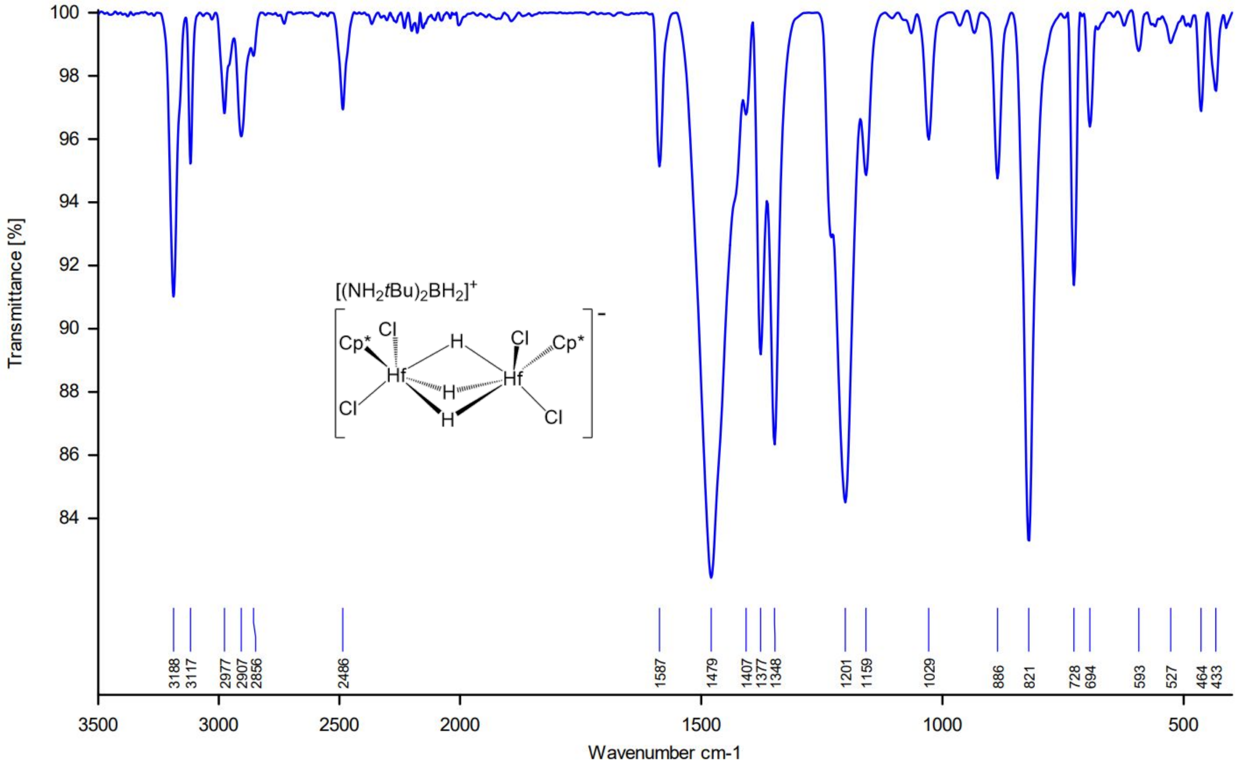


**Figure S18.** IR spectrum (KBr) of $[(NH_2t\text{Bu})_2BH_2][\{Hf(\eta^5\text{-}C_5Me_5)Cl_2\}_2(\mu\text{-}H)_3]$ (**10**).

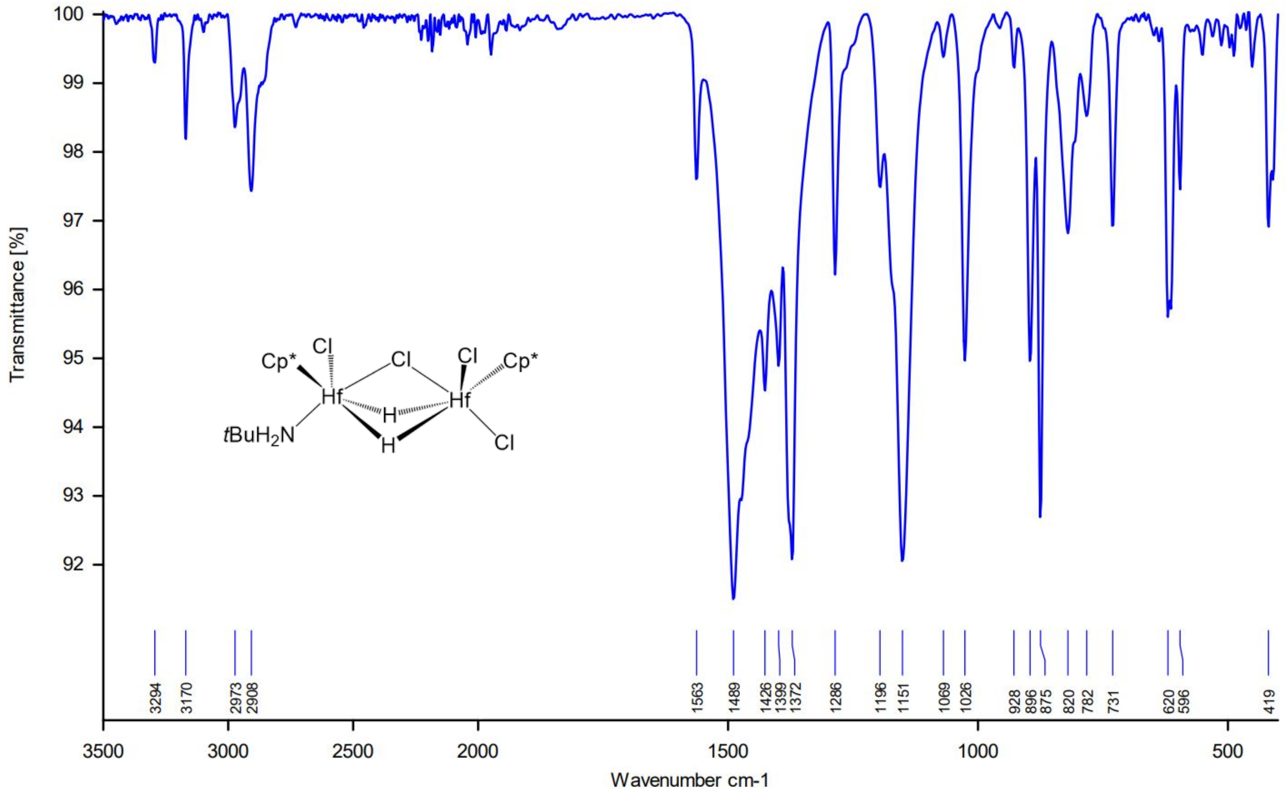


**Figure S19.** IR spectrum (ATR) of $[Cl_2(\eta^5\text{-}C_5Me_5)Hf(\mu\text{-}Cl)(\mu\text{-}H)_2Hf(\eta^5\text{-}C_5Me_5)Cl(NH_2t\text{Bu})]$ (**12**).

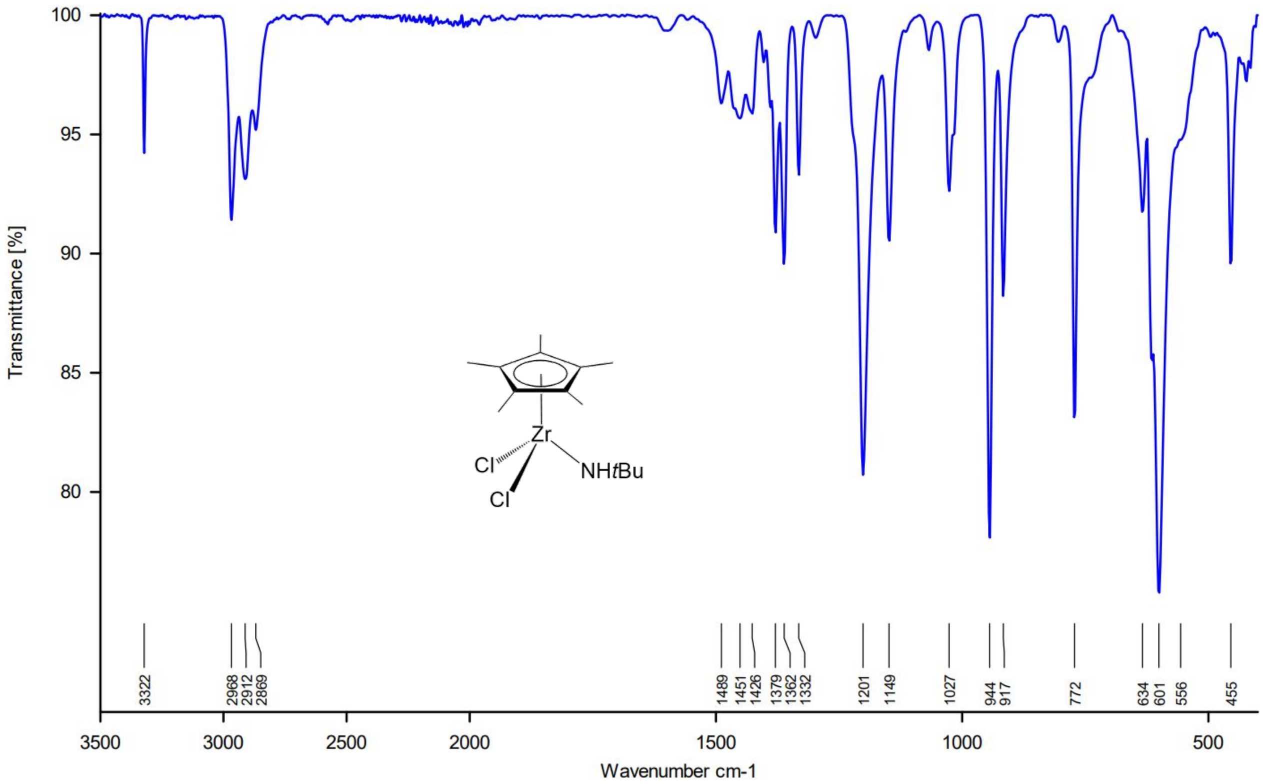


**Figure S20.** IR spectrum (ATR) of [Zr($\eta^5$-$C_5Me_5$)$Cl_2$(NH*t*Bu)] (**13**).

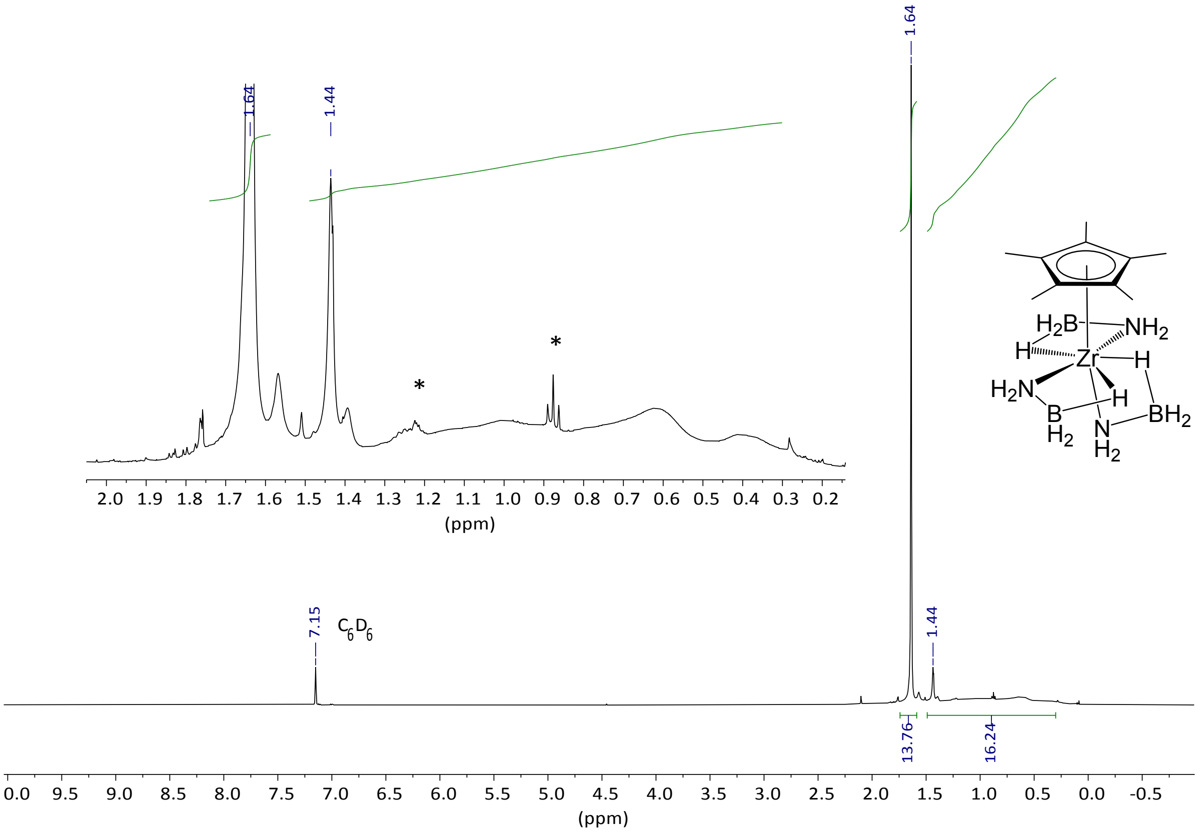


**Figure S21.** $^1$H NMR spectrum (500 MHz, $C_6D_6$, 20 ºC) of [Zr($\eta^5$-$C_5Me_5$)($NH_2BH_3$)$_3$] (**1**). **n*-Hexane

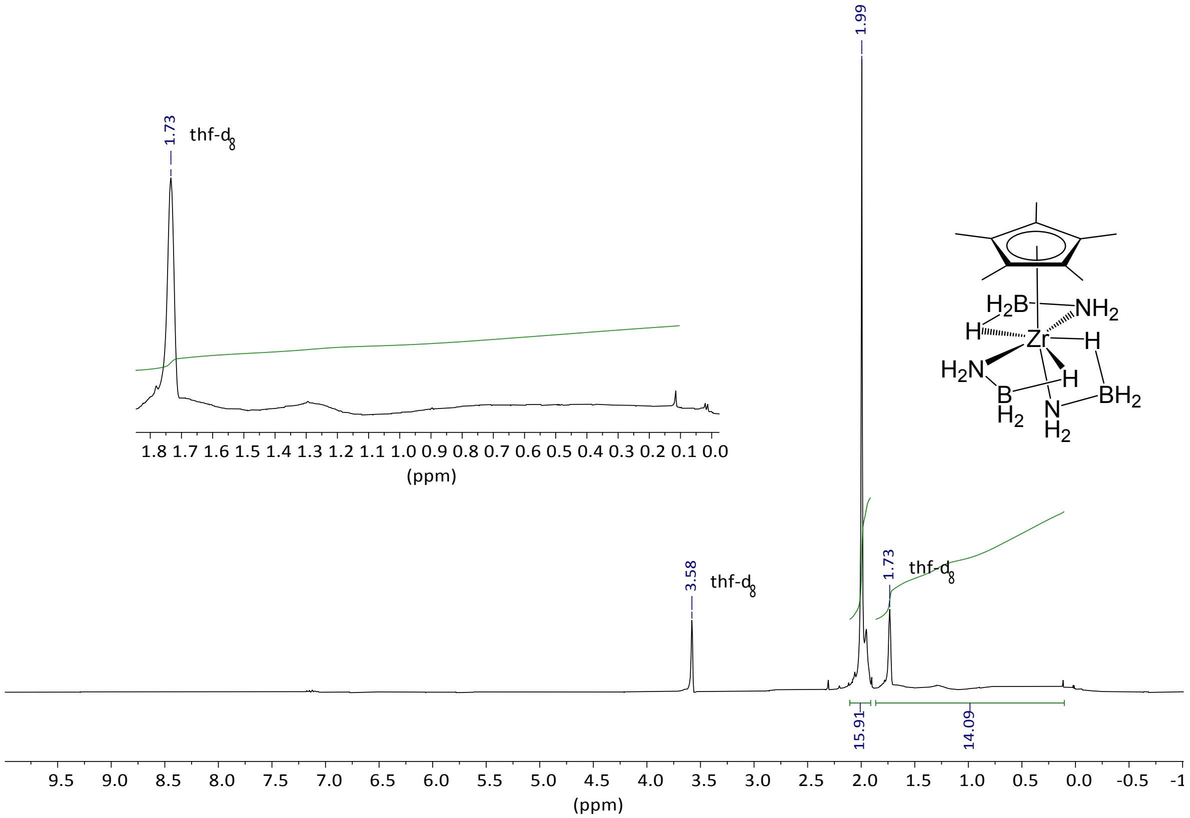


**Figure S22.** $^{1}H$ NMR spectrum (300 MHz, $C_4D_8O$, 20 ºC) of [Zr($\eta^5$-$C_5Me_5$)($NH_2BH_3$)$_3$] (**1**).

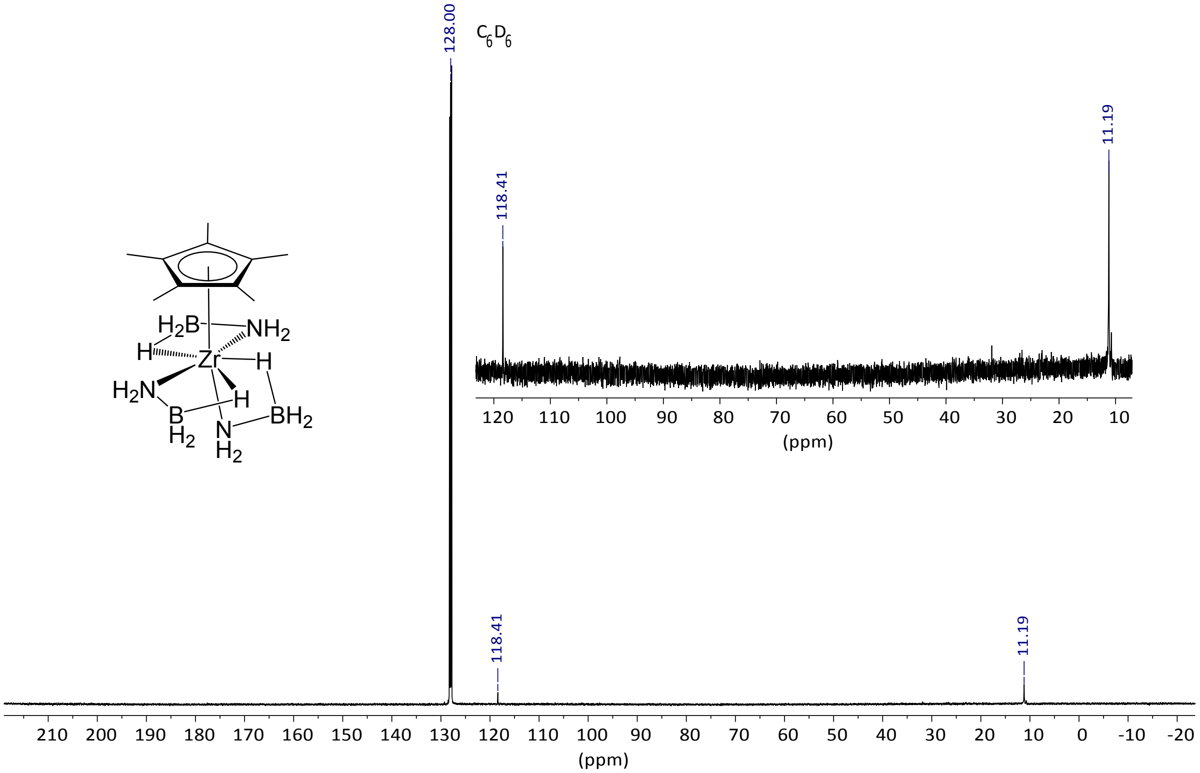


**Figure S23.** $^{13}C\{^{1}H\}$ NMR spectrum (125 MHz, $C_6D_6$, 20 ºC) of [Zr($\eta^5$-$C_5Me_5$)($NH_2BH_3$)$_3$] (**1**).

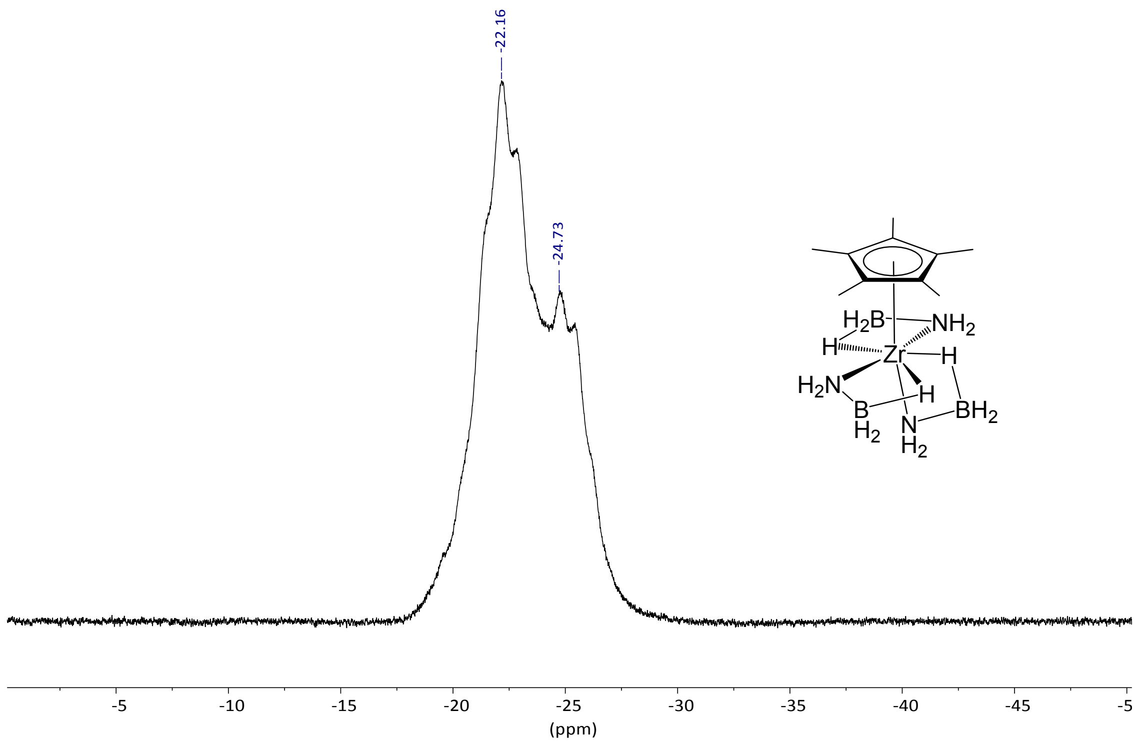


**Figure S24.** $^{11}B$ NMR spectrum (128 MHz, $C_6D_6$, 20 ºC) of [Zr($\eta^5$-$C_5Me_5$)($NH_2BH_3$)$_3$] (**1**).

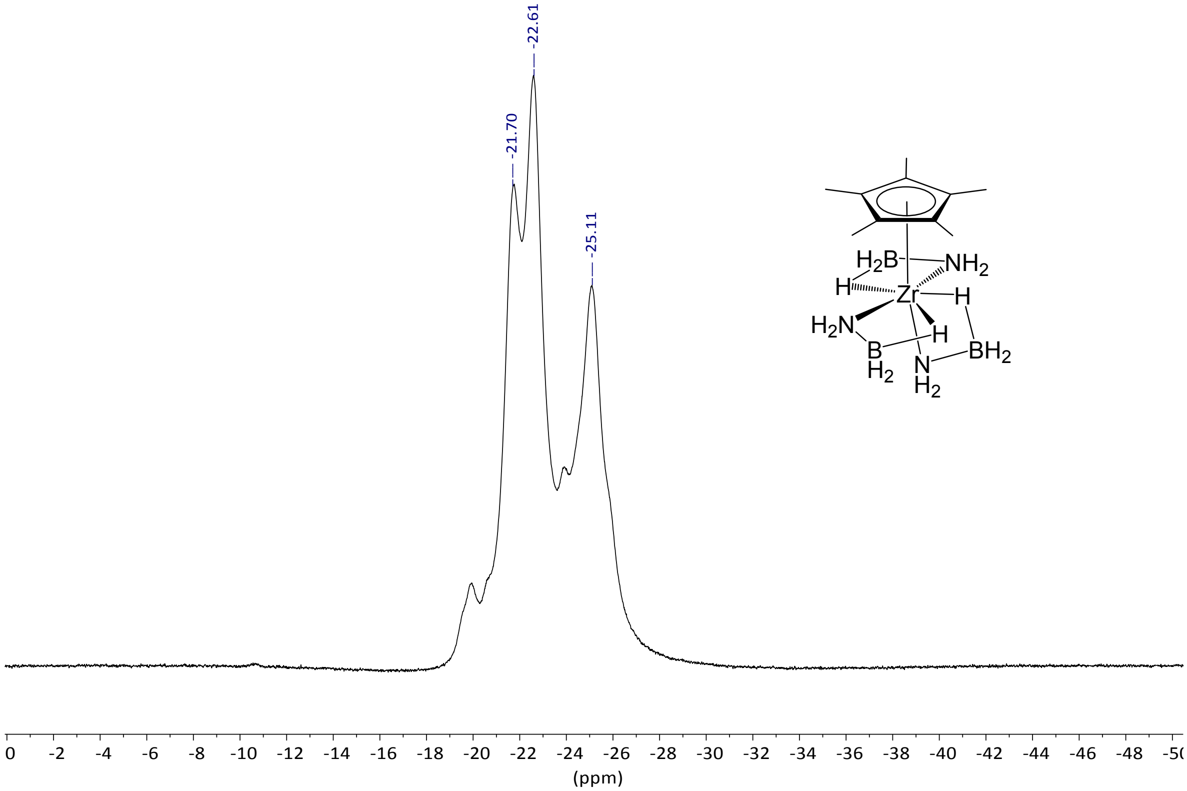


**Figure S25.** $^{11}B\{^1H\}$ NMR spectrum (128 MHz, $C_6D_6$, 20 ºC) of [Zr($\eta^5$-$C_5Me_5$)($NH_2BH_3$)$_3$] (**1**).

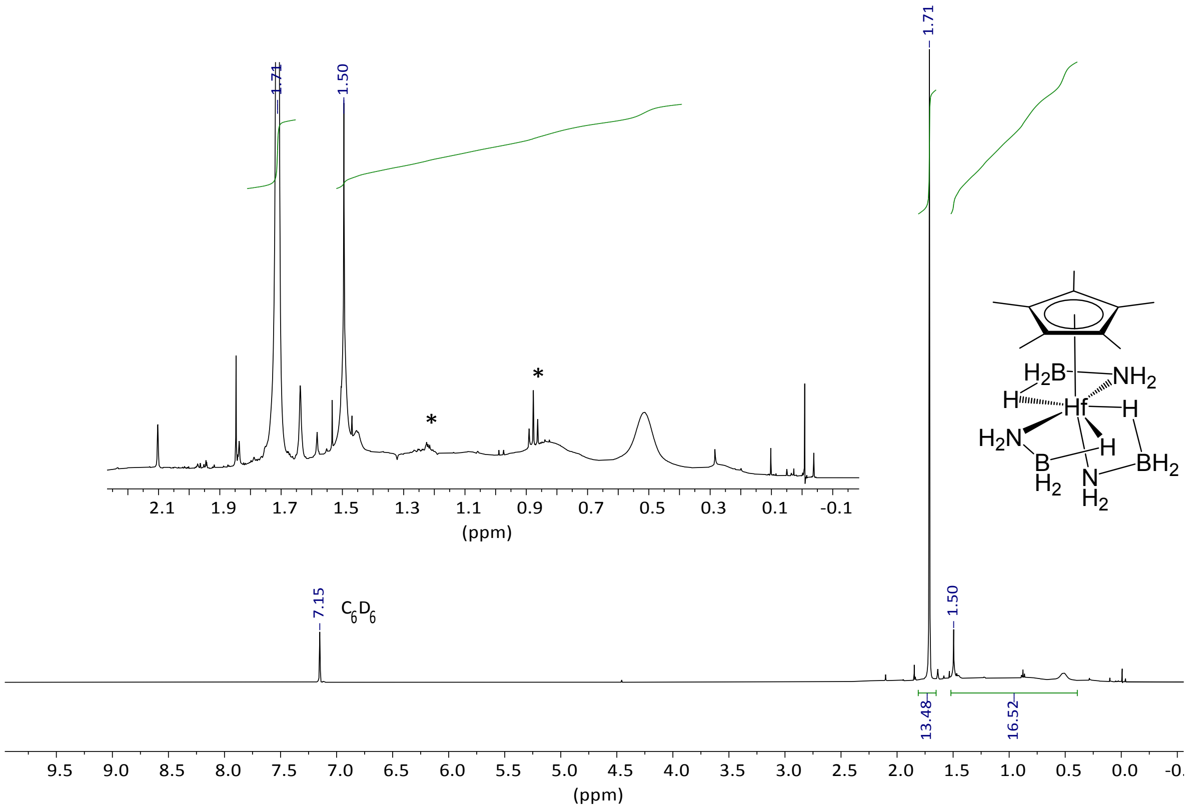


**Figure S26.** $^{1}H$ NMR spectrum (500 MHz, $C_6D_6$, 20 ºC) of $[Hf(\eta^5\text{-}C_5Me_5)(NH_2BH_3)_3]$ (**2**). **n*-Hexane

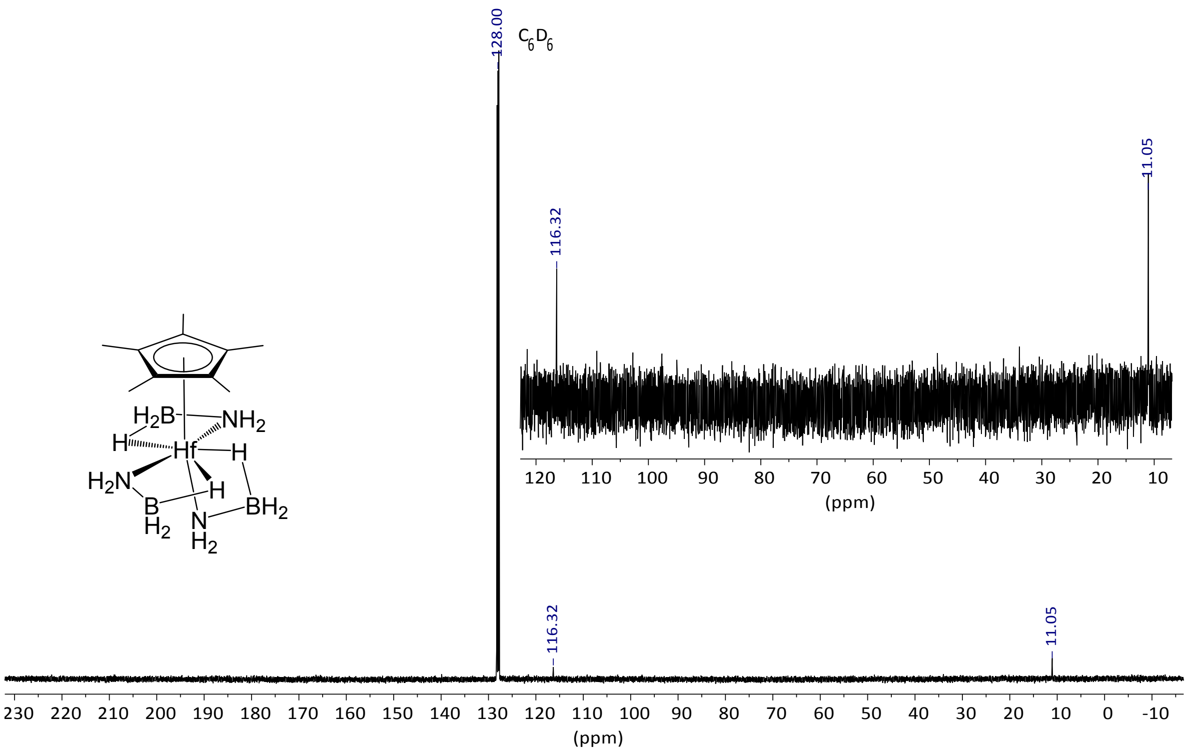


**Figure S27.** $^{13}C\{^{1}H\}$ NMR spectrum (125 MHz, $C_6D_6$, 20 ºC) of $[Hf(\eta^5\text{-}C_5Me_5)(NH_2BH_3)_3]$ (**2**).

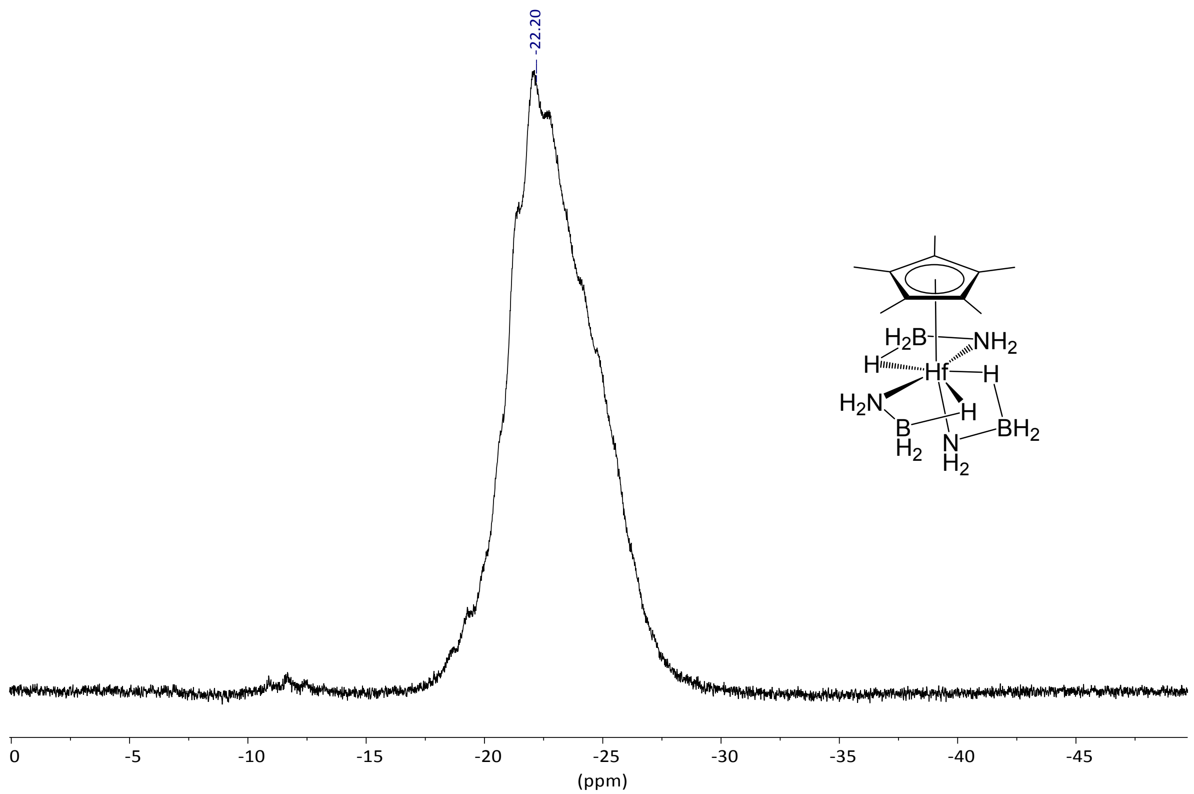


**Figure S28.** $^{11}B$ NMR spectrum (128 MHz, $C_6D_6$, 20 ºC) of [Hf($\eta^5$-$C_5Me_5$)($NH_2BH_3$)$_3$] (**2**).

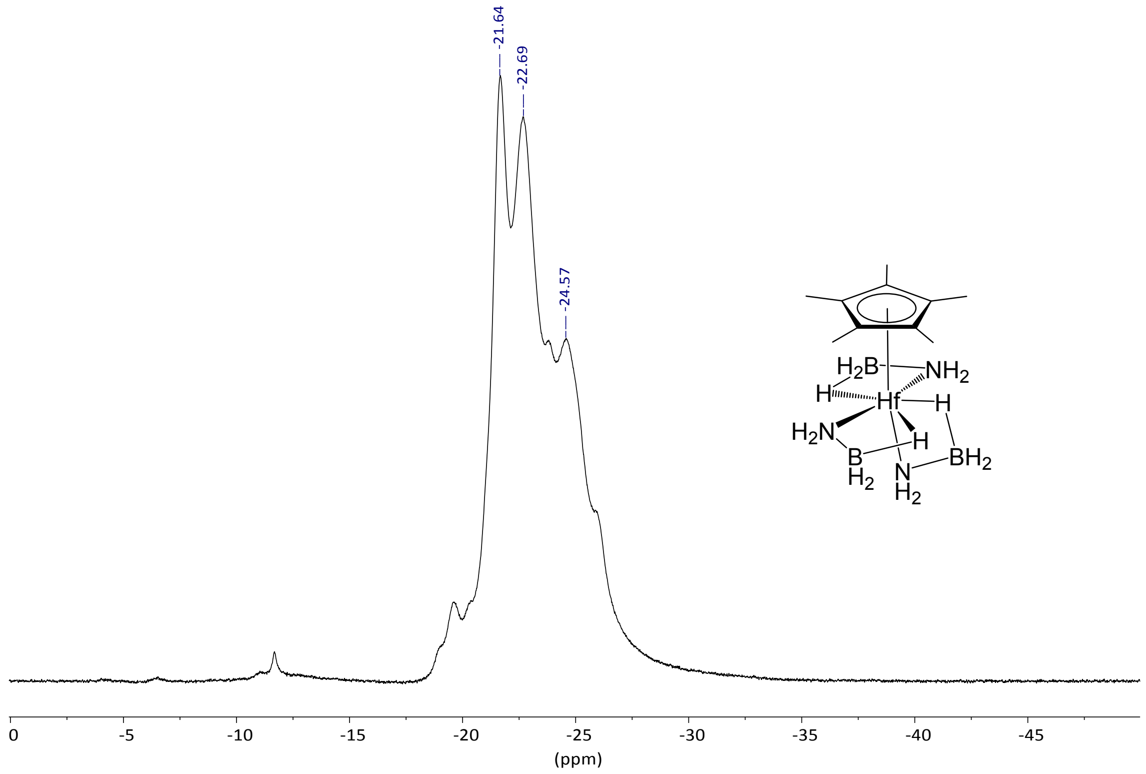


**Figure S29.** $^{11}B\{^1H\}$ NMR spectrum (128 MHz, $C_6D_6$, 20 ºC) of [Hf($\eta^5$-$C_5Me_5$)($NH_2BH_3$)$_3$] (**2**).

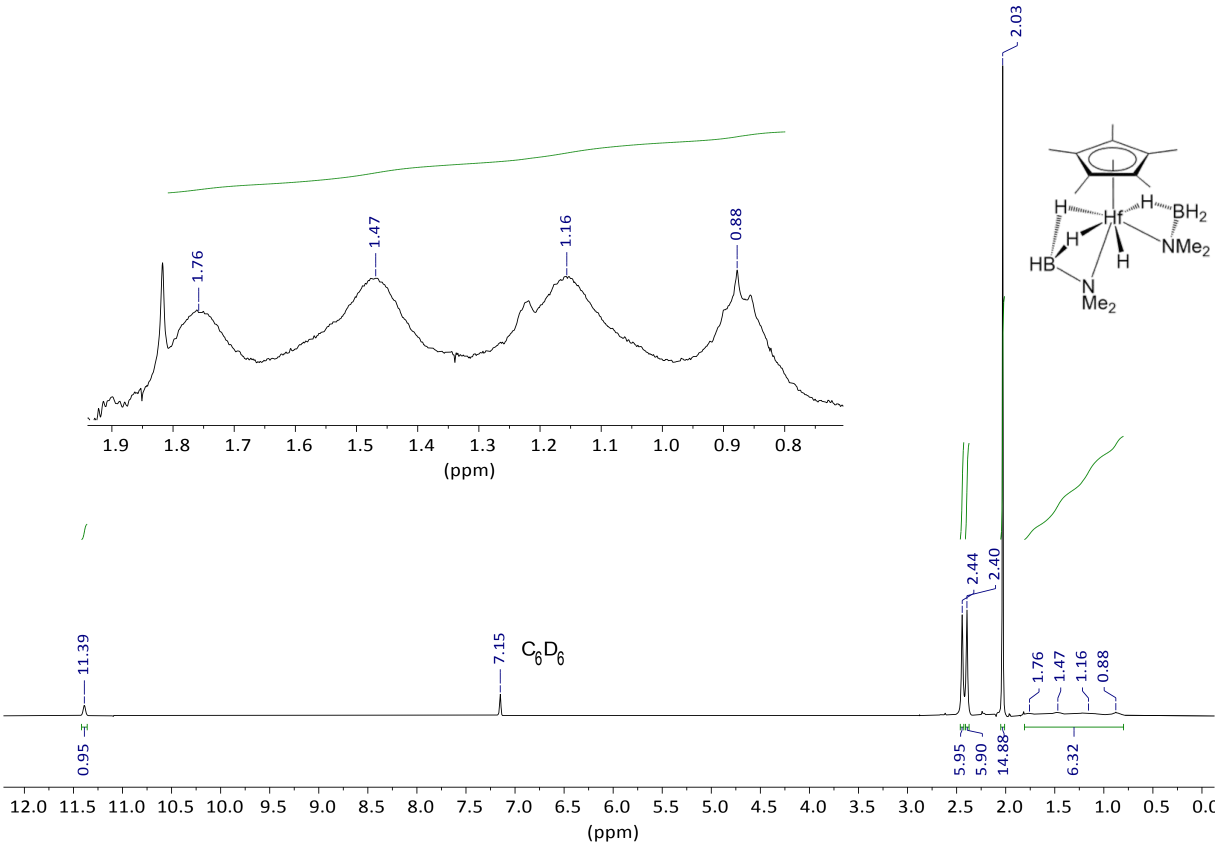


**Figure S30.** $^{1}$H NMR spectrum (300 MHz, $C_6D_6$, 20 ºC) of [Hf($\eta^5$-$C_5Me_5$)H($NMe_2BH_3$)$_2$] (**4**).

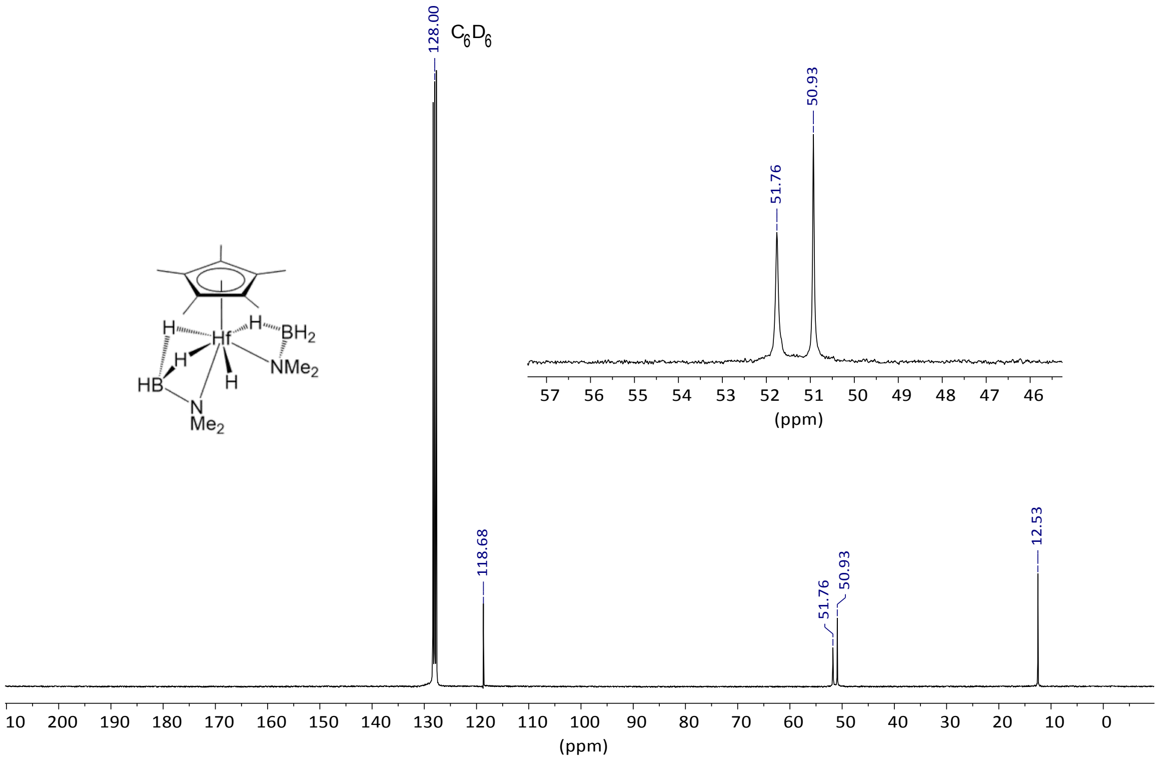


**Figure S31.** $^{13}$C{$^{1}$H} NMR spectrum (75 MHz, $C_6D_6$, 20 ºC) of [Hf($\eta^5$-$C_5Me_5$)H($NMe_2BH_3$)$_2$] (**4**).

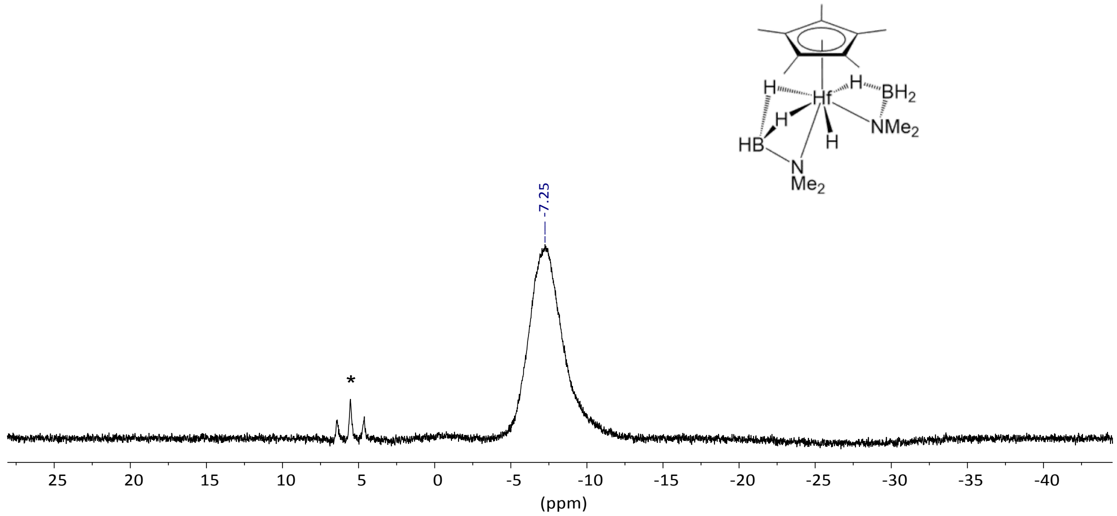


**Figure S32.** $^{11}B$ NMR spectrum (128 MHz, $C_6D_6$, 20 ºC) of [Hf($\eta^5$-$C_5Me_5$)H($NMe_2BH_3$)$_2$] (**4**). *($NMe_2BH_2$)$_2$.

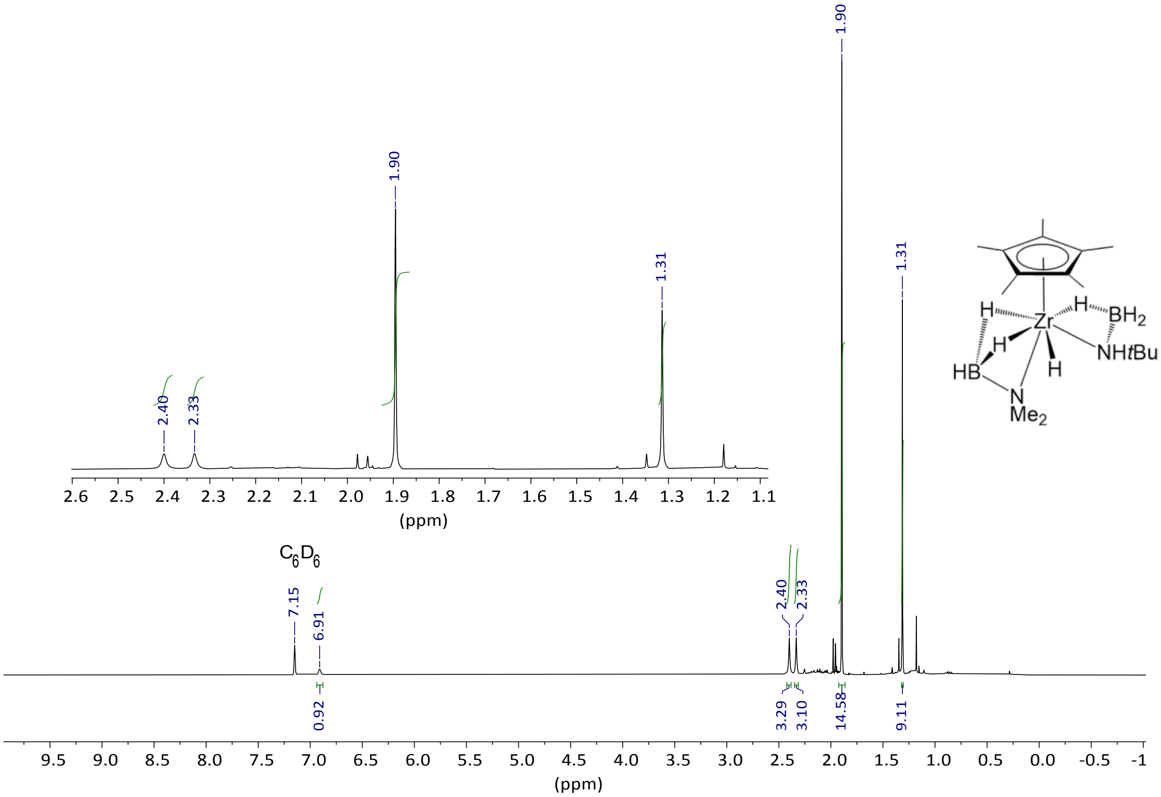


**Figure S33.** $^{1}H$ NMR spectrum (300 MHz, $C_6D_6$, 20 ºC) of [Zr($\eta^5$-$C_5Me_5$)H(NH*t*Bu$BH_3$)($NMe_2BH_3$)] (**5**).

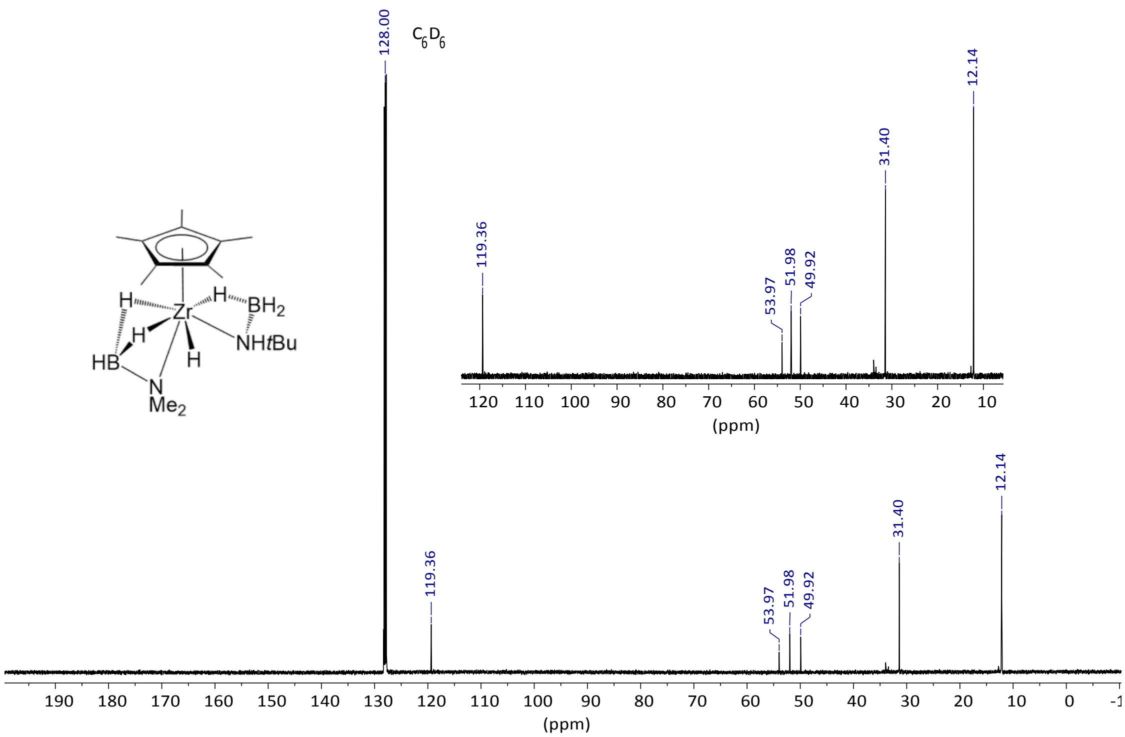


**Figure S34.** $^{13}C\{^1H\}$ NMR spectrum (125 MHz, $C_6D_6$, 20 ºC) of [Zr(η$^5$-$C_5Me_5$)H(NH*t*Bu$BH_3$)($NMe_2BH_3$)] (**5**).

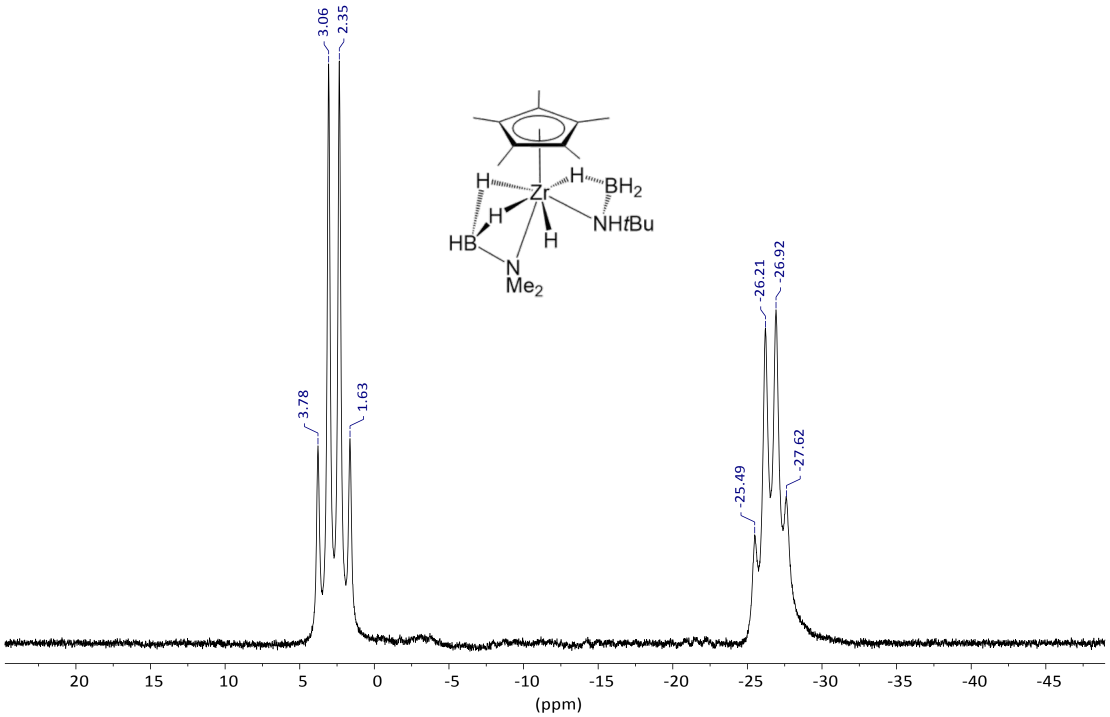


**Figure S35.** $^{11}B$ NMR spectrum (128 MHz, $C_6D_6$, 20 ºC) of [Zr(η$^5$-$C_5Me_5$)H(NH*t*Bu$BH_3$)($NMe_2BH_3$)] (**5**).

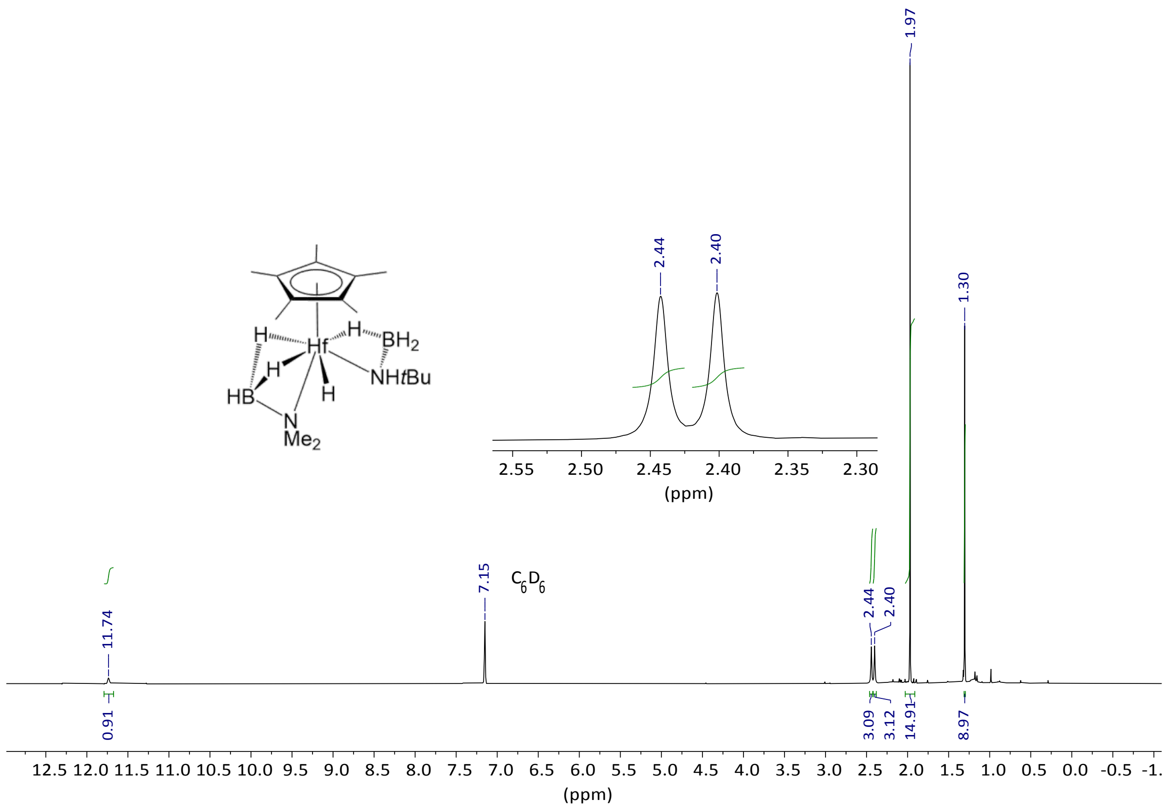

**Figure S36.** $^{1}H$ NMR spectrum (300 MHz, $C_6D_6$, 20 ºC) of [Hf($\eta^5$-$C_5Me_5$)H(NH*t*Bu$BH_3$)($NMe_2BH_3$)] (**6**).

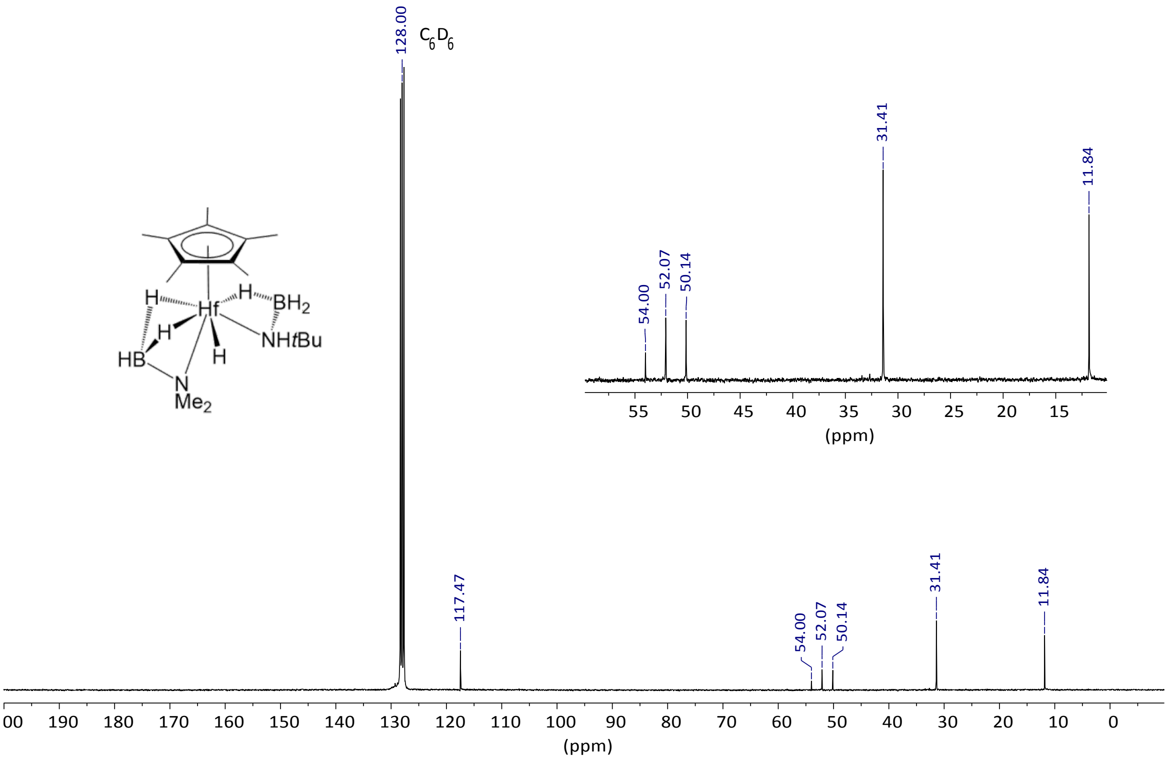

**Figure S37.** $^{13}C\{^{1}H\}$ NMR spectrum (75 MHz, $C_6D_6$, 20 ºC) of [Hf($\eta^5$-$C_5Me_5$)H(NH*t*Bu$BH_3$)($NMe_2BH_3$)] (**6**).

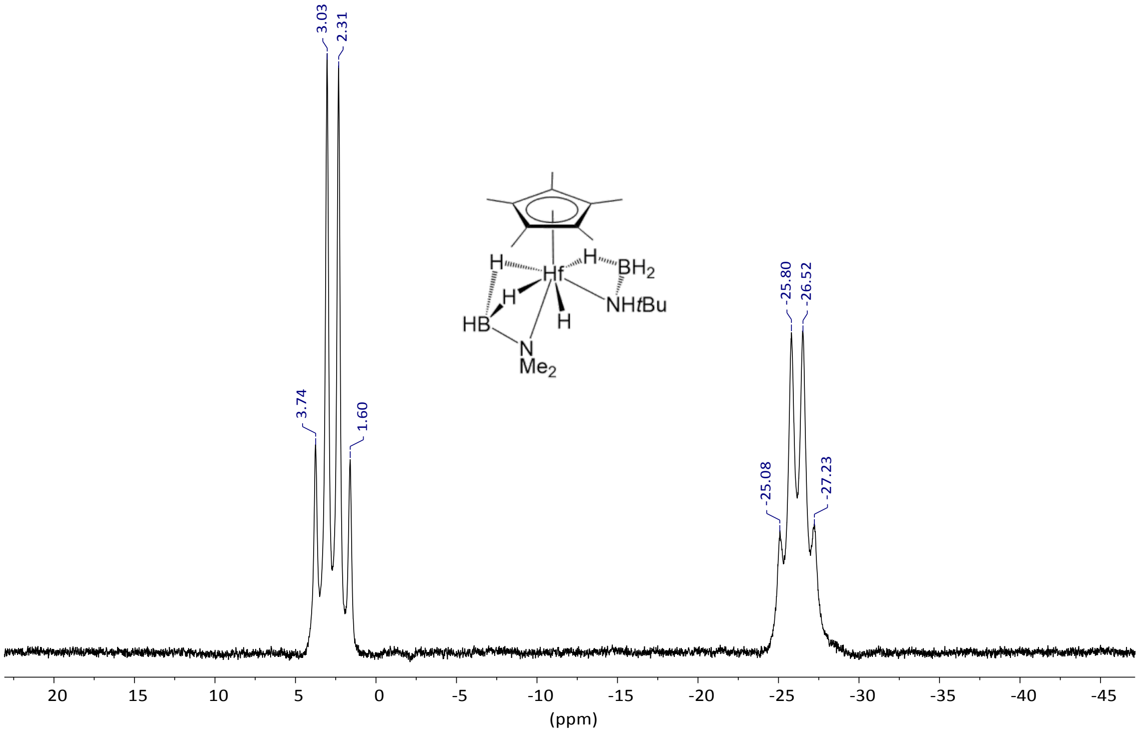


**Figure S38.** $^{11}B$ NMR spectrum (128 MHz, $C_6D_6$, 20 ºC) of [Hf($\eta^5$-$C_5Me_5$)H(NH*t*Bu$BH_3$)($NMe_2BH_3$)] (**6**).

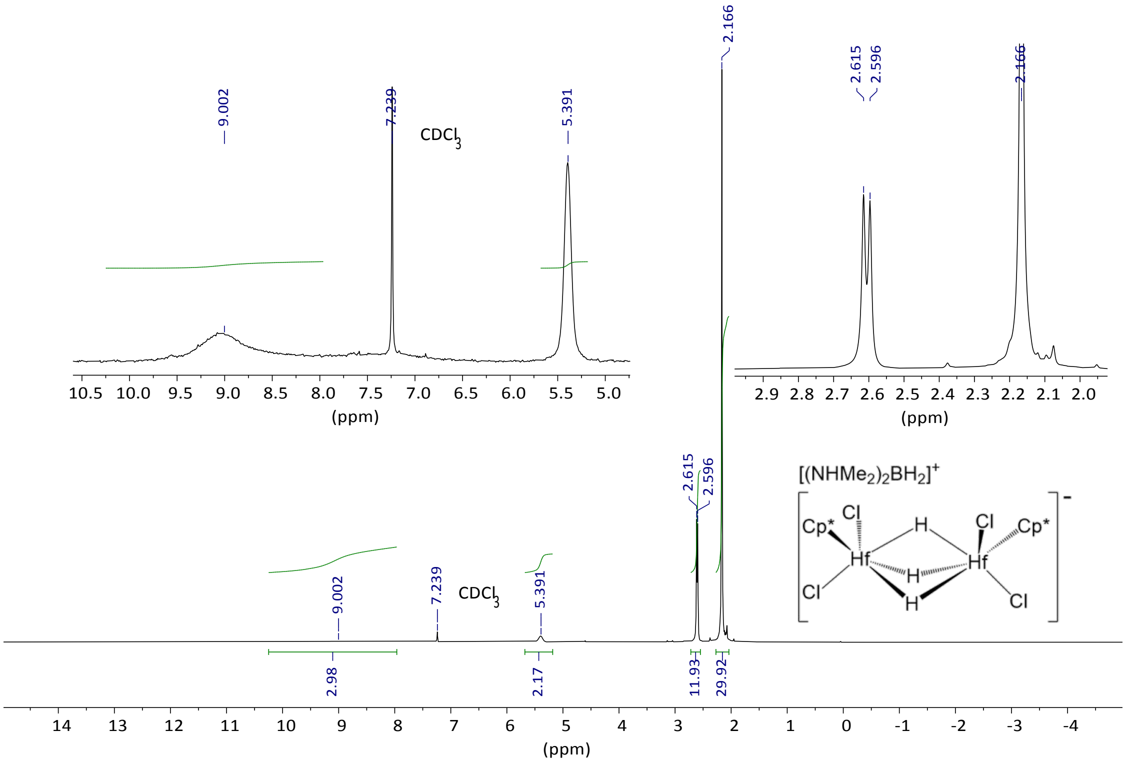


**Figure S39.** $^1H$ NMR spectrum (300 MHz, $CDCl_3$, 20 ºC) of [$(NHMe_2)_2BH_2$][{Hf($\eta^5$-$C_5Me_5$)$Cl_2$}$_2$($\mu$-H)$_3$] (**8**).

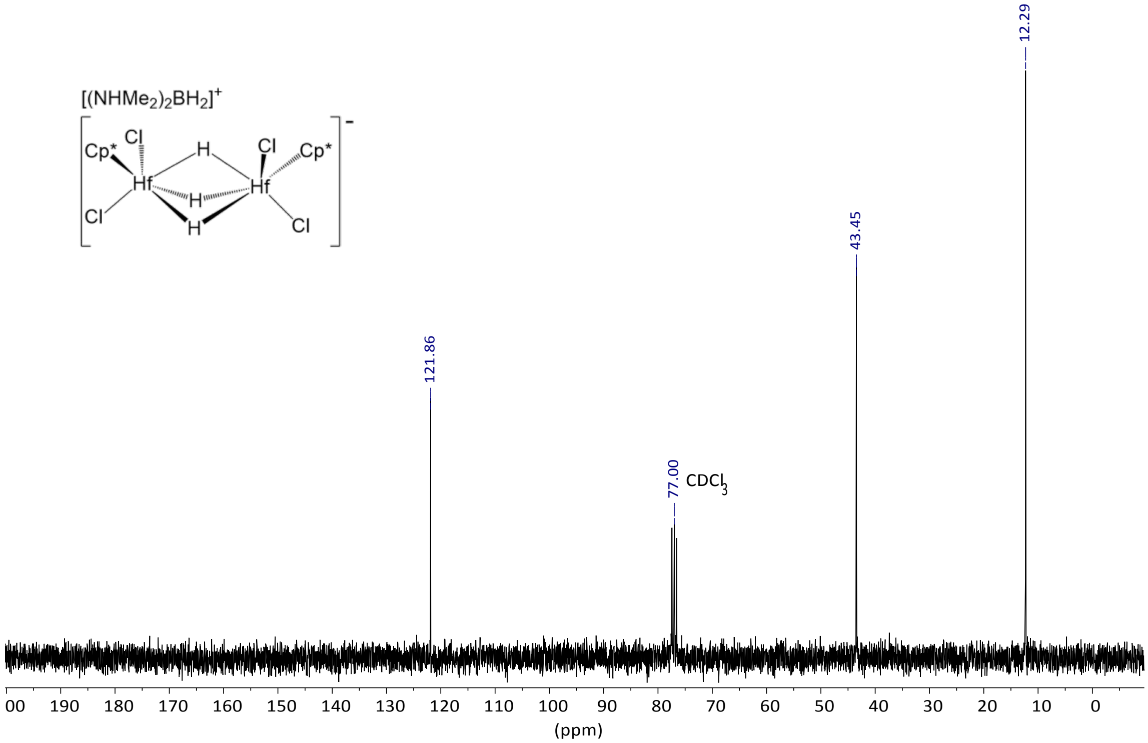


**Figure S40.** $^{13}C\{^1H\}$ NMR spectrum (75 MHz, $CDCl_3$, 20 ºC) of $[(NHMe_2)_2BH_2][\{Hf(\eta^5\text{-}C_5Me_5)Cl_2\}_2(\mu\text{-}H)_3]$ (**8**).

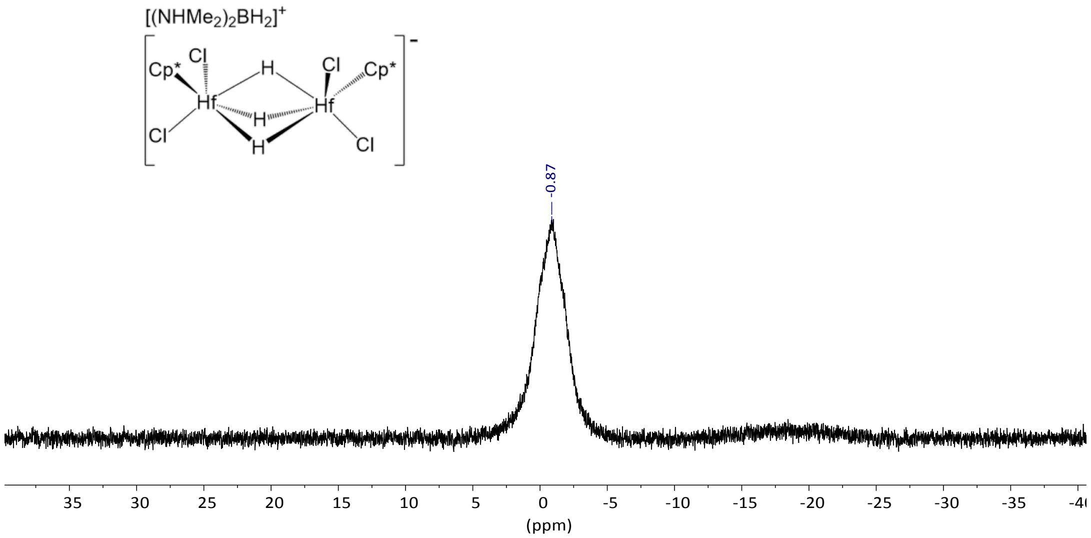


**Figure S41.** $^{11}B$ NMR spectrum (128 MHz, $CDCl_3$, 20 ºC) of $[(NHMe_2)_2BH_2][\{Hf(\eta^5\text{-}C_5Me_5)Cl_2\}_2(\mu\text{-}H)_3]$ (**8**).

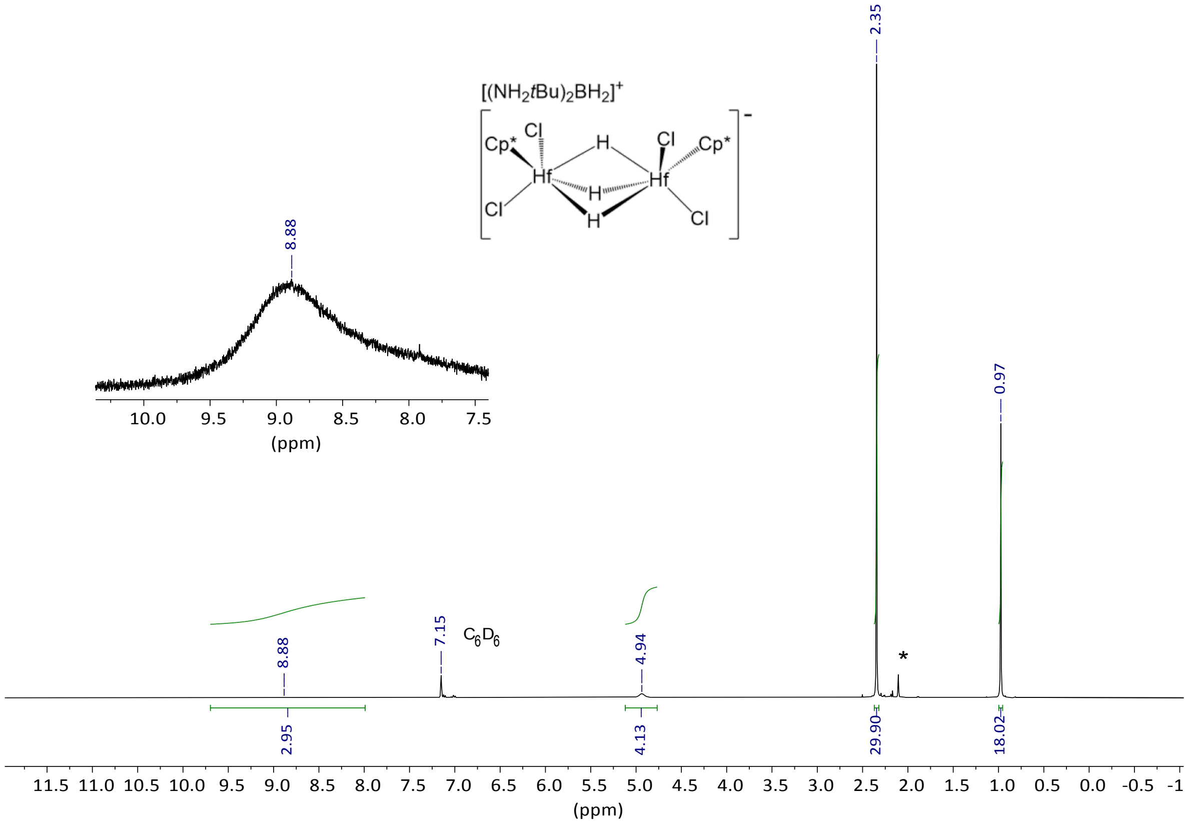


**Figure S42.** $^{1}H$ NMR spectrum (400 MHz, $C_6D_6$, 20 ºC) of $[(NH_2tBu)_2BH_2][\{Hf(\eta^5\text{-}C_5Me_5)Cl_2\}_2(\mu\text{-}H)_3]$ (**10**).
*Toluene

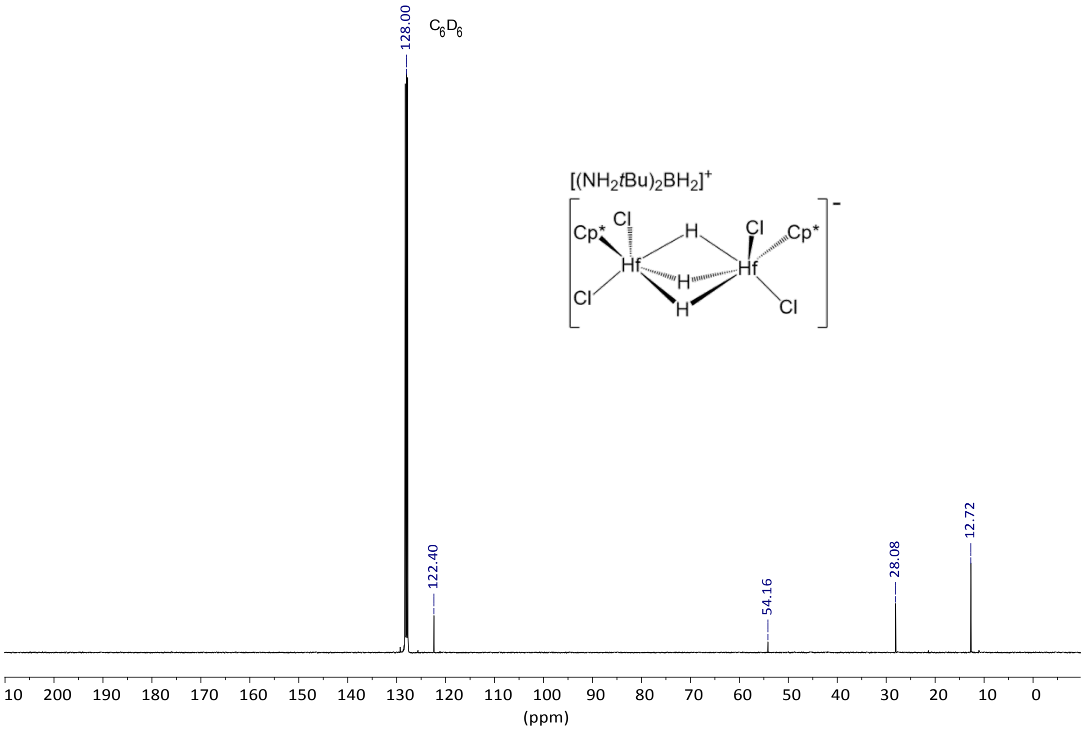


**Figure S43.** $^{13}C\{^{1}H\}$ NMR spectrum (100 MHz, $C_6D_6$, 20 ºC) of $[(NH_2tBu)_2BH_2][\{Hf(\eta^5\text{-}C_5Me_5)Cl_2\}_2(\mu\text{-}H)_3]$ (**10**).

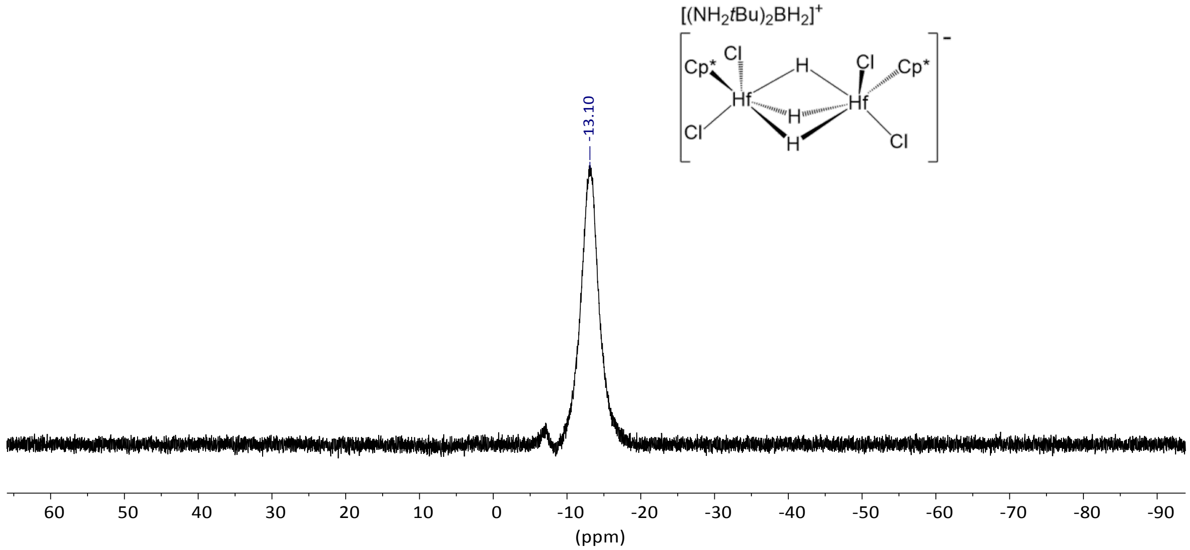


**Figure S44.** $^{11}$B NMR spectrum (128 MHz, $C_6D_6$, 20 ºC) of $[(NH_2tBu)_2BH_2][\{Hf(\eta^5\text{-}C_5Me_5)Cl_2\}_2(\mu\text{-}H)_3]$ (**10**).

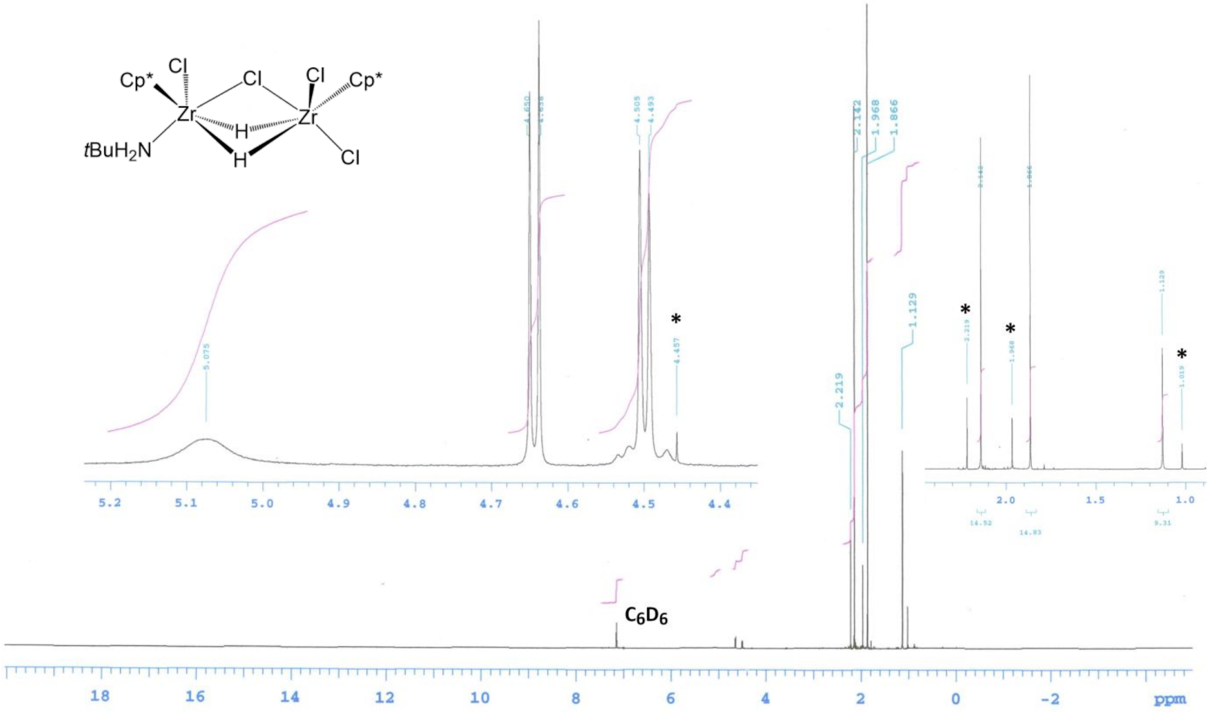


**Figure S45.** $^1$H NMR spectrum (500 MHz, $C_6D_6$, 20 ºC) of $[Cl_2(\eta^5\text{-}C_5Me_5)Zr(\mu\text{-}H)_2(\mu\text{-}Cl)Zr(\eta^5\text{-}C_5Me_5)Cl(NH_2tBu)]$ (**11**). *Unknown compound.

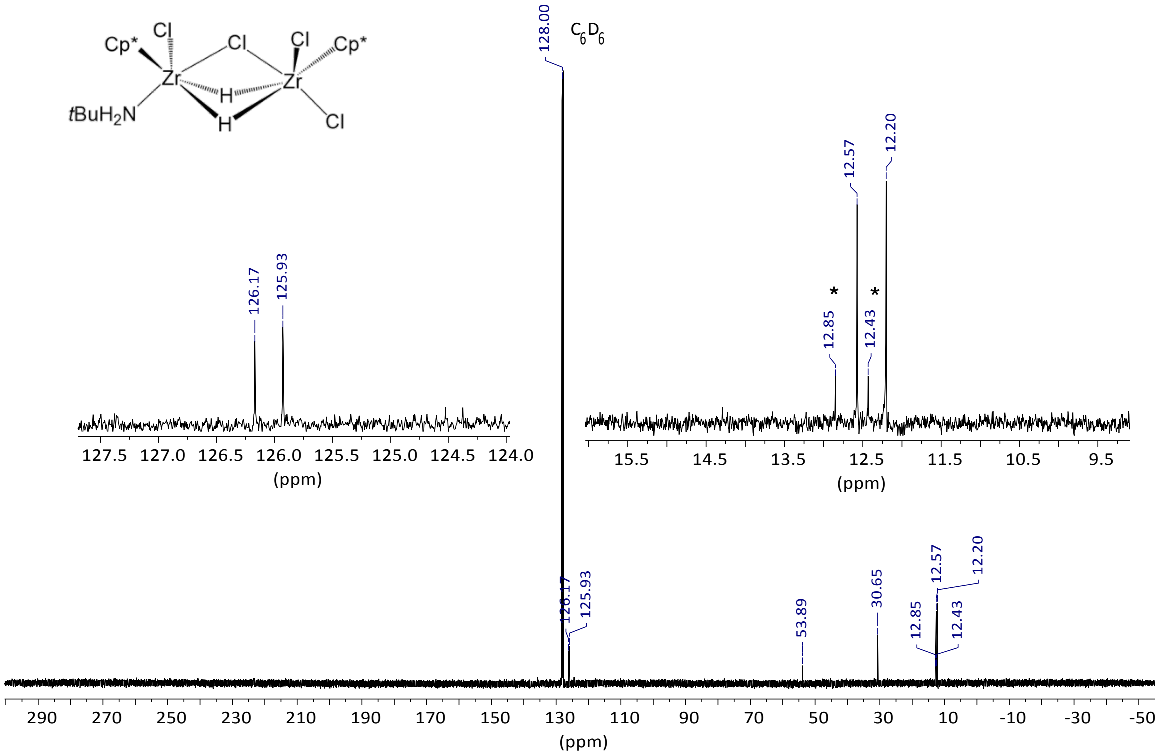


**Figure S46.** $^{13}C\{^{1}H\}$ NMR spectrum (125 MHz, $C_6D_6$, 20 ºC) of [$Cl_2(\eta^5-C_5Me_5)Zr(\mu-Cl)(\mu-H)_2Zr(\eta^5-C_5Me_5)Cl(NH_2$*t*Bu)] (**11**). *Unknown compound.

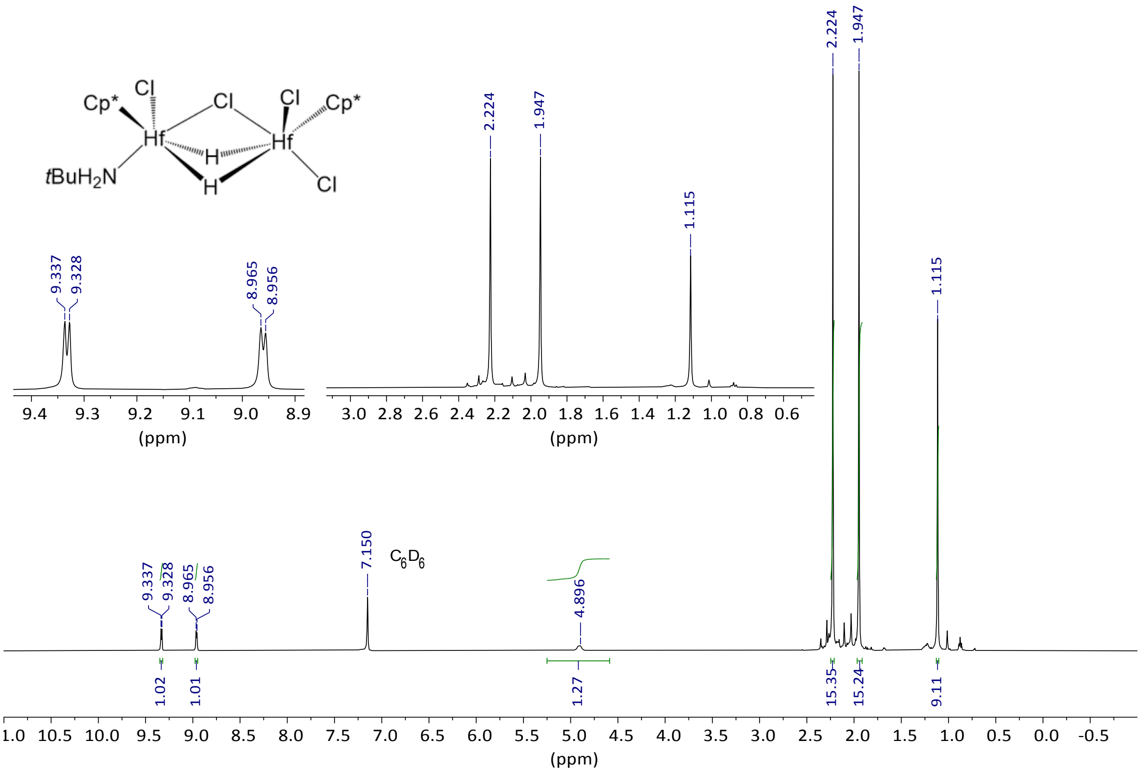


**Figure S47.** $^{1}H$ NMR spectrum (500 MHz, $C_6D_6$, 20 ºC) of [$Cl_2(\eta^5-C_5Me_5)Hf(\mu-Cl)(\mu-H)_2Hf(\eta^5-C_5Me_5)Cl(NH_2$*t*Bu)] (**12**).

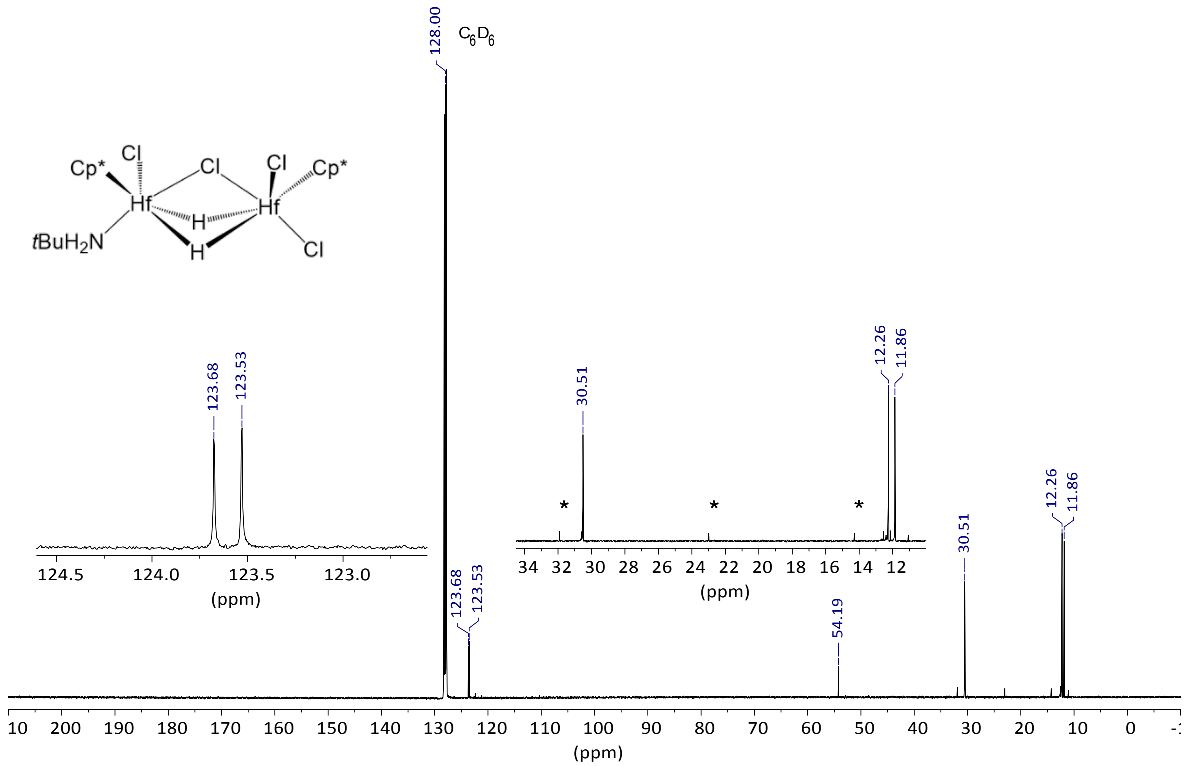

**Figure S48.** $^{13}C\{^{1}H\}$ NMR spectrum (125 MHz, $C_6D_6$, 20 ºC) of [$Cl_2$($\eta^5$-$C_5Me_5$)Hf(μ-Cl)(μ-H)$_2$Hf($\eta^5$-$C_5Me_5$)Cl($NH_2$*t*Bu)] (**12**). **n*-Hexane.

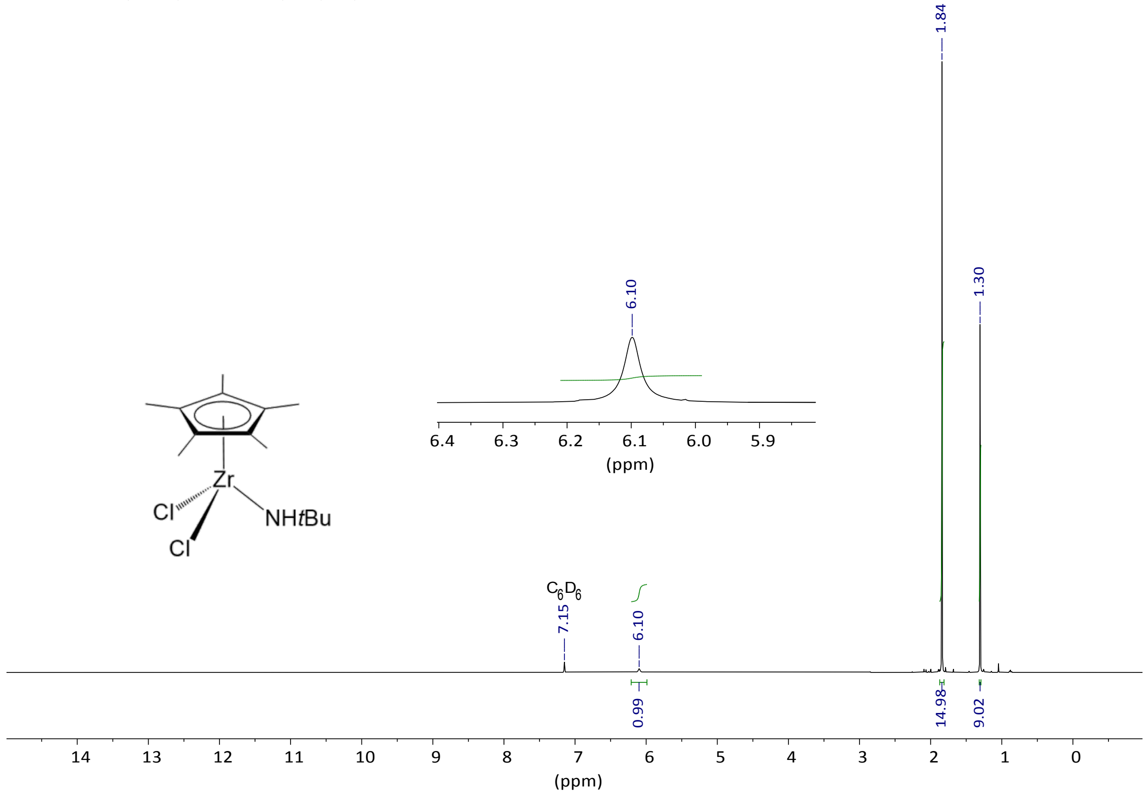

**Figure S49.** $^{1}H$ NMR spectrum (400 MHz, $C_6D_6$, 20 ºC) of [Zr($\eta^5$-$C_5Me_5$)$Cl_2$(NH*t*Bu)] (**13**).

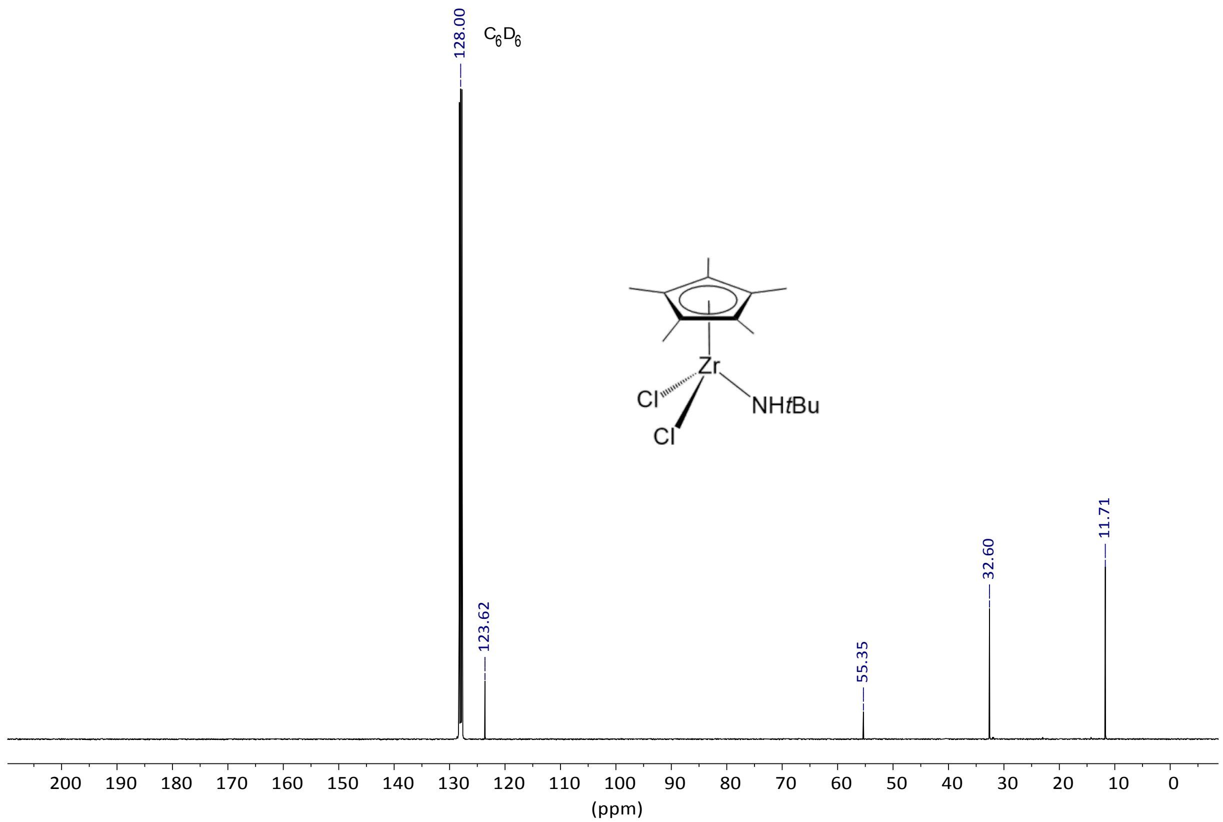


**Figure S50.** $^{13}C\{^1H\}$ NMR spectrum (100 MHz, $C_6D_6$, 20 ºC) of [Zr($\eta^5$-$C_5Me_5$)$Cl_2$(NH*t*Bu)] (**13**).
